# A Complex Comprising a Cyanine Dye Rotaxane and a Porphyrin Nanoring as a Model Light‐Harvesting System†

**DOI:** 10.1002/anie.202006644

**Published:** 2020-07-23

**Authors:** Jiratheep Pruchyathamkorn, William J. Kendrick, Andrew T. Frawley, Andrea Mattioni, Felipe Caycedo‐Soler, Susana F. Huelga, Martin B. Plenio, Harry L. Anderson

**Affiliations:** ^1^ Department of Chemistry Oxford University Chemistry Research Laboratory Oxford OX1 3TA UK; ^2^ Institute of Theoretical Physics and IQST Ulm University Albert-Einstein-Allee 11 89069 Ulm Germany

**Keywords:** Cyanine dyes, excitonic coupling, light harvesting, porphyrin, rotaxanes

## Abstract

A nanoring‐rotaxane supramolecular assembly with a Cy7 cyanine dye (hexamethylindotricarbocyanine) threaded along the axis of the nanoring was synthesized as a model for the energy transfer between the light‐harvesting complex LH1 and the reaction center in purple bacteria photosynthesis. The complex displays efficient energy transfer from the central cyanine dye to the surrounding zinc porphyrin nanoring. We present a theoretical model that reproduces the absorption spectrum of the nanoring and quantifies the excitonic coupling between the nanoring and the central dye, thereby explaining the efficient energy transfer and demonstrating similarity with structurally related natural light‐harvesting systems.

## Graphical abstract

**Colorful to the core**: Rotaxane formation was used to lock a cyanine dye along the axis of a porphyrin nanoring to form a complex that mimics the photophysical behavior of the light‐harvesting complex LH1 and the reaction center in purple photosynthetic bacteria. Energy is transferred rapidly between the axial dye and the surrounding nanoring.

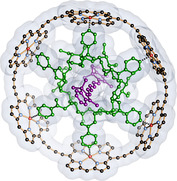

Living organisms achieve photosynthesis using a wide diversity of supramolecular chlorophyll arrays to absorb sunlight and channel energy to a reaction center (RC), where it is converted into chemical potential.^1^ The design requirements for an efficient light‐harvesting system are poorly understood, despite many investigations of natural and artificial chromophore arrays.^2^, ^3^ All species of purple bacteria use an assembly known as light‐harvesting complex 1 (LH1), which surrounds the RC. There is some variation in the structures of the LH1/RC complexes from different species of bacteria, but they typically consist of a ring of about 32 bacteriochlorophyll molecules encircling the RC.^4^ The energy‐transfer process from LH1 to the RC is intriguing because it occurs rapidly (within about 50 ps) despite being reversible and thermodynamically uphill; back transfer from the RC to LH1 is even faster, occurring within a few picoseconds.^5^ Several artificial systems have been synthesized as models of the LH1/RC supercomplex to investigate energy transfer between a ring of chromophores and a central acceptor/donor chromophore.^3^, ^6^ Here, we present the design and synthesis of a supramolecular dye complex inspired by the LH1/RC system, and examine its photophysical properties.

Porphyrin nanorings offer a rigid cyclic array of absorbing chromophores, resembling the ring of bacteriochlorophyll pigments in LH1. These nanorings are synthesized using templates of the correct geometry to bind the metal centers of each metalloporphyrin unit. A variety of oligopyridine ligands can be used as templates,^7^ including pyridine‐functionalized cyclodextrins (CDs).^8^ Oligopyridine templates bind strongly to the nanorings, with association constants (*K*_f_) in the range 10^29^–10^36^ m ^−1^.^9^ These compounds provide a unique toolkit with which to mimic light‐harvesting systems.^10^

In order to model the LH1/RC supercomplex, a chromophore must be positioned at the center of the porphyrin nanoring to mimic the RC. α‐CD is an ideal scaffold for achieving this spatial arrangement: its six primary OH groups can be functionalized with 4‐pyridyl substituents to form a template (T6*) for a six‐porphyrin nanoring (***c*** **‐P6**), while its hydrophobic cavity can be used to encapsulate guest molecules, such as the hexamethylindotricarbocyanine dye (HITC, Cy7), via rotaxane formation.^11^ This concept led us to target the structure Cy7⊂*c*‐P6⋅T6* (Figure 1 and Scheme 1): the ***c*** **‐P6** and Cy7 units mimic the LH1 and RC respectively, and the α‐CD fixes the spatial arrangement of the chromophores. Molecular mechanics calculations predicted two low‐energy conformations for Cy7⊂*c*‐P6⋅T6*, labeled as **A** and **B** in Figure 1. Both conformations have the cyanine dye aligned along the axis of the porphyrin nanoring. In conformation **A**, the center of the cyanine is near the plane of the six zinc centers, whereas in **B**, the Cy7⊂T6* unit is shifted towards one rim of the nanoring. The calculated energies of these conformations are almost the same (**A** is lower in energy by 0.7 kJ mol^−1^). We expect them both to be populated and to interconvert rapidly in solution on a ms timescale.

**Figure 1:**
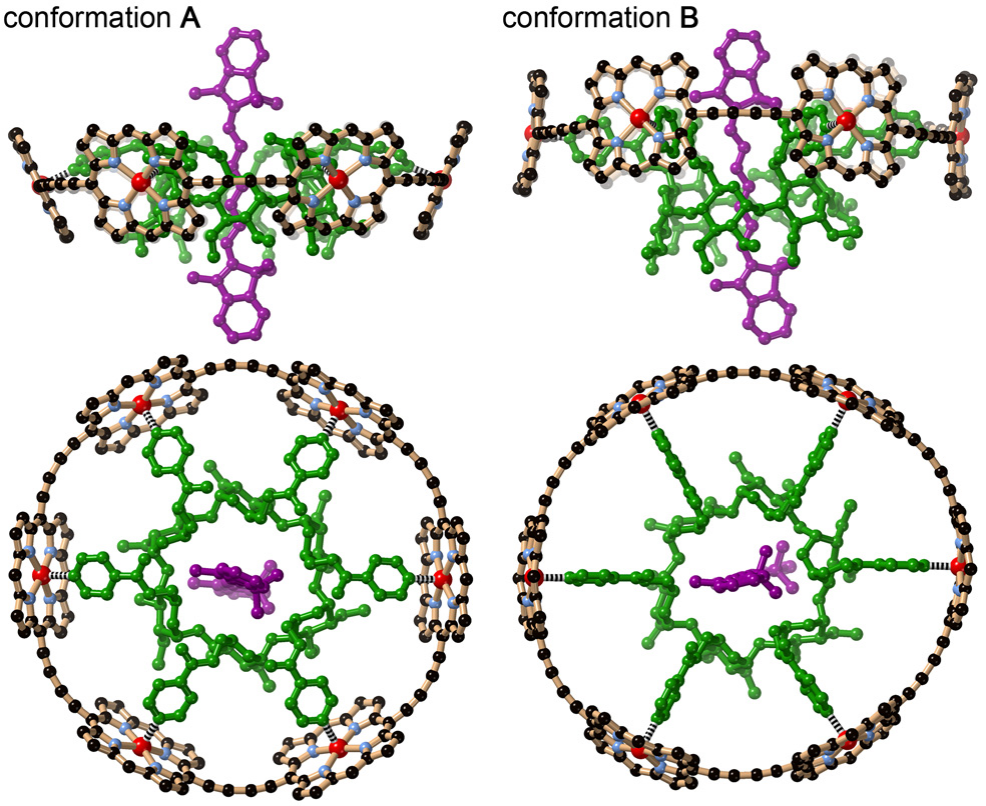
Two low‐energy conformations of Cy7⊂*c*‐P6⋅T6* from molecular mechanics calculations; two orthogonal views of each conformation (MM+ force field, *meso*‐aryl groups and hydrogen atoms are omitted for clarity, see Section S3 in the Supporting Information for details).

**Scheme 1:**
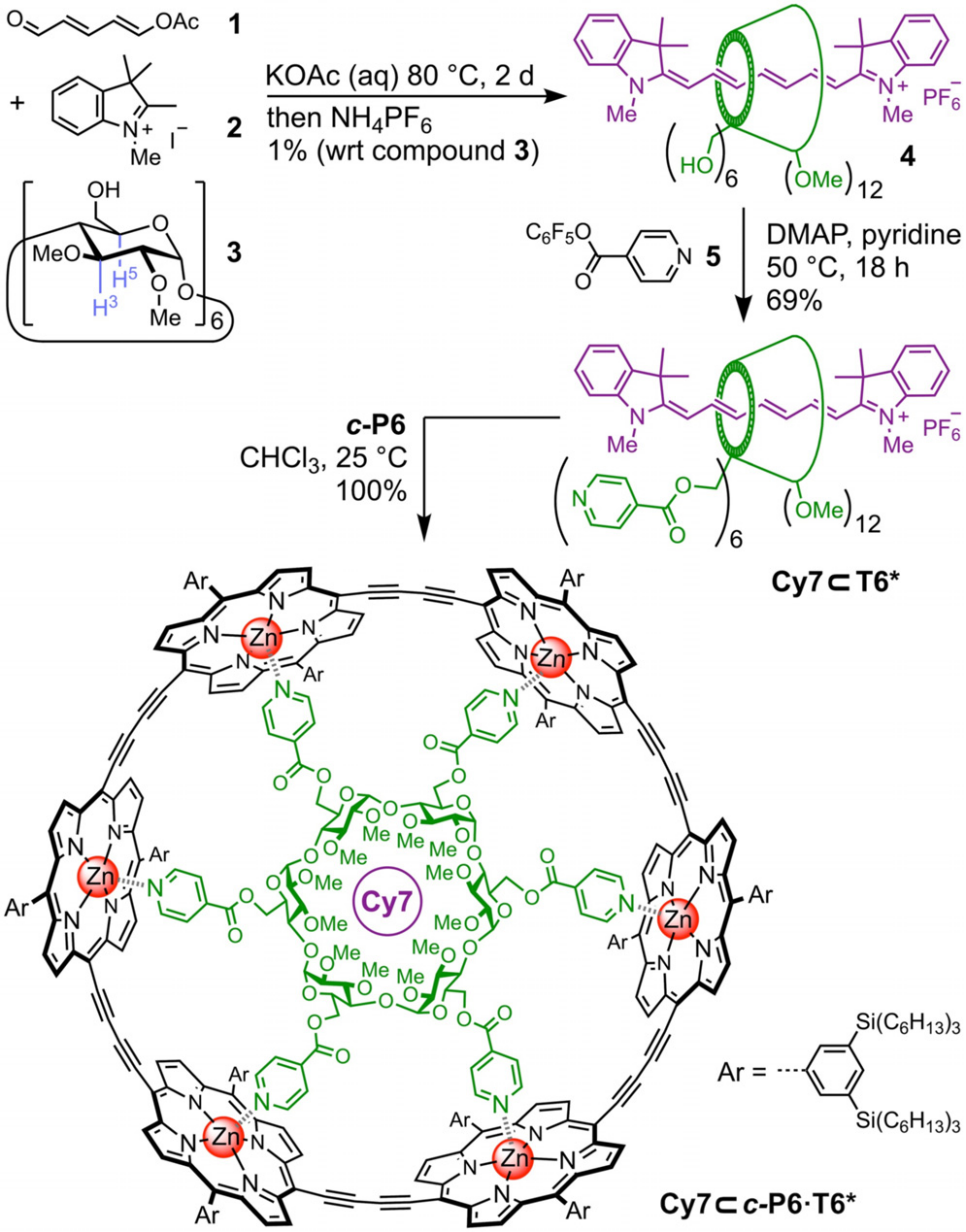
Synthesis of Cy7⊂*c*‐P6⋅T6*. Cyanine dye Cy7 in purple; functionalized CD and T6* in green.

The synthesis of Cy7⊂*c*‐P6⋅T6* was achieved as summarized in Scheme 1. The first step is the reaction of 5‐oxopenta‐1,3‐dienyl acetate (**1**), tetramethyl indolium iodide (**2**), and per‐2,3‐di‐*O*‐methyl‐α‐cyclodextrin^12^ (**3**) in aqueous potassium acetate to form rotaxane **4**. The yield of this rotaxane is low (1 %, based on **3**), but sufficient material can be prepared to complete the synthesis. Previously, the unmethylated analogue of rotaxane **4** was prepared in 13 % yield, based on **1** as the limiting reagent, using a 9‐fold excess of native α‐CD.^11^ In the synthesis of **4**, it is not practical to use an excess of **3** because this methylated cyclodextrin is less readily available than the other components **1** and **2**. Acylation of all six hydroxy groups of rotaxane **4** with isonicotinic acid pentafluorophenyl ester **5** gave Cy7⊂T6* in 69 % yield. Addition of this ligand to ***c*** **‐P6** resulted in immediate and quantitative formation of the desired complex Cy7⊂*c*‐P6⋅T6*. The three rotaxanes, **4**, Cy7⊂T6* and Cy7⊂*c*‐P6⋅T6*, were thoroughly characterized by mass spectrometry and NMR spectroscopy, and their ^1^H NMR spectra were fully assigned using COSY and NOESY techniques, as detailed in the Supporting Information. In all of these compounds, the asymmetric environment of the α‐CD makes all four methyl groups of the cyanine dye non‐equivalent, which proves that the Cy7 is inside the α‐CD, as confirmed by the observation of NOEs from protons 3 and 5 of the α‐CD to protons on the Cy7 bridge. (H^3^ and H^5^ are the two inwardly pointing protons of the cyclodextrin, marked in blue in Scheme 1.) In Cy7⊂*c*‐P6⋅T6*, the asymmetry of the α‐CD results in eight distinct porphyrin β‐pyrrole doublets, and the pattern of NOEs demonstrates the close proximity of all three components (***c*** **‐P6**, CD, and Cy7). The observation of NOEs from the α‐CD‐OMe groups to *meso*‐aryl protons of the nanoring, and to the β‐pyridyl resonances, is consistent with conformation **A**, in which the α‐CD sits tightly within the nanoring.

Electronic coupling between Cy7 and ***c*** **‐P6** would be expected to result in changes in the intensity and/or wavelength of the optical transitions of the Cy7⊂*c*‐P6⋅T6* complex, compared with the transitions of free Cy7⊂T6* and ***c*** ‐P6⋅T6*, as predicted for the bacterial LH1/RC supercomplex.^13^ The absorption spectrum of the Cy7⊂*c*‐P6⋅T6* complex is compared with the sum of the spectra of its components in Figure 2. The spectrum of the target complex is similar to the sum of the absorption spectra of its individual components. The three‐peak structure of the Q‐band is left mostly unaltered, thus suggesting that the spectral changes induced by a coherent excitonic coupling between the nanoring and the central dye may be too subtle to be detected experimentally. Nonetheless, as we can assess theoretically, this coupling is similar in magnitude to the excitonic interaction between LH1 and RC pigments^13^ (see the Supporting Information) and can influence strongly other photophysical properties of Cy7⊂*c*‐P6⋅T6*, such as energy‐transfer dynamics, as we will show later.

**Figure 2:**
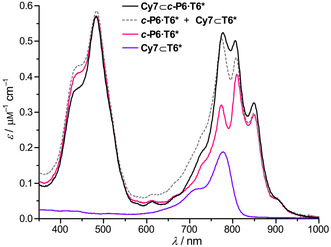
Absorption spectra of Cy7⊂*c*‐P6⋅T6* (black), ***c*** ‐P6⋅T6* (red), Cy7⊂T6* (purple), and the addition of absorption spectra of ***c*** ‐P6⋅T6*+Cy7⊂T6* (gray dashed line), in CH_2_Cl_2_ at 25 °C.

The fluorescence behavior of Cy7⊂*c*‐P6⋅T6* was examined to test how well it mimics the LH1/RC complexes. In Figure 3, the fluorescence spectra of Cy7⊂*c*‐P6⋅T6* and Cy7⊂T6* are compared. Both samples were excited at 725 nm in CH_2_Cl_2_, and the intensities of the spectra are scaled so that their areas are proportional to the fluorescence quantum yields multiplied by the molar absorption coefficient at 725 nm, to give the normalized brightness. The cyanine dye rotaxane Cy7⊂T6* exhibits a bright emission band at 798 nm (quantum yield: *Φ*_f_=36 %). In contrast, Cy7⊂*c*‐P6⋅T6* is weakly fluorescent (*Φ*_f_=0.6 %; *λ*_max_=910 nm), and its brightness at 798 nm is 0.0051 (relative to 1.0 for Cy7⊂T6*, both excited at 725 nm). Thus the presence of the surrounding nanoring quenches more than 99 % of the emission from the cyanine core of Cy7⊂*c*‐P6⋅T6*. The emission spectra and quantum yields of Cy7⊂*c*‐P6⋅T6* and ***c*** ‐P6⋅T6* are essentially the same (see Figure S37 in the Supporting Information), which implies that there is efficient energy transfer from Cy7 to ***c*** **‐P6** in Cy7⊂*c*‐P6⋅T6*. The fluorescence lifetime of Cy7⊂T6* is 0.97 ns (see the Supporting Information), so the reduction in brightness by a factor of >99 % indicates that energy transfer occurs on a time scale shorter than 10 ps. The excitation spectrum of Cy7⊂*c*‐P6⋅T6* (measured at the emission maximum at 910 nm; Figure S36) matches the absorption spectrum, thus indicating that energy absorbed at all wavelengths is transferred and emitted with the same efficiency. The fact that the surrounding ring fluoresces, emulates analogous observations for the LH1/RC supramolecular complex.^14^ Although the Cy7 dye and the ***c*** **‐P6** ring absorb light almost independently, as discussed above (Figure 2), the energy absorbed by the chromophores in both structures is transferred to the lowest energy excited state efficiently before fluorescence occurs. If the energy transfer were not efficient, emission would take place from all the transitions that absorb light and with a significant contribution from the Cy7 dye, since it has a larger fluorescence quantum yield than the ring (Table S5). In contrast, we verify that emission occurs from this lowest‐energy state regardless of the excitation wavelength (Figure S36), in line with efficient exciton‐transfer dynamics enabling fluorescence after thermal equilibration to the lowest‐energy state of the full dye‐ring complex. In order to clarify the mechanism that leads to the efficient energy transfer that we observe experimentally (Figure 3), we propose a microscopic model based on Davydov–Frenkel theory of molecular excitons^15^ which describes the electronic transitions of the Q‐band in terms of coupled transition dipoles associated to individual porphyrins (for more details, see Section S7 in the Supporting Information). This model reproduces the main structure (optical transitions and line shapes) in the absorption spectrum of the nanoring (Figure 4 a, for conformer **A**, Section S7 in the Supporting Information for more details). Moreover, it predicts a small but persistent dipole redistribution from the 810 nm band of the nanoring to the central dye due to a small coherent dye‐nanoring coupling (ca. 25 cm^−1^, Figure 4 b), consistent with predictions for the LH1/RC supercomplex.^13^ Although the coupling is too small to be observed directly in the experimental absorption spectra of Figure 2, it leads to very efficient energy transfer from the dye to the nanoring, typically within 3 ps (Figure 4 c). This value perfectly matches our estimates based on fluorescence measurements, thus further validating our theoretical model. Analogous simulations for conformer **B** led to only slight changes in the nanoring‐dye couplings and transfer rates. As a result, energy transfer takes place on the same timescale as for conformer **A**, yielding an average rate of 1/(4.4 ps). Since this value is still much faster than fluorescence from the dye, the interpretation of the underlying photophysics remains unaltered.

**Figure 3:**
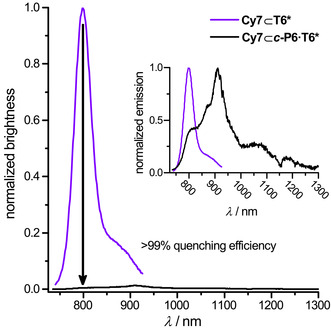
Normalized brightness of fluorescence spectra for Cy7⊂T6* and Cy7⊂*c*‐P6⋅T6* excited at 725 nm. Normalized brightness is calculated by scaling the areas of the emission spectra by their quantum yields and to their molar absorption coefficients at 725 nm. The ratio of intensities at 798 nm is 0.0051. Inset shows the normalized emission spectra. Spectra recorded in CH_2_Cl_2_ at 25 °C.

**Figure 4:**
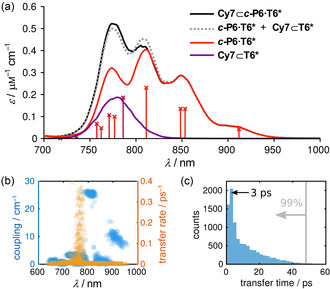
Theoretical description of absorption spectra and transfer dynamics using an excitonic model system (see the Supporting Information for details). a) Theoretical absorption spectra of Cy7⊂*c*‐P6⋅T6* (black), ***c*** ‐P6⋅T6* (red), Cy7⊂T6* (purple), and the addition of theoretical absorption spectra of ***c*** ‐P6⋅T6*+Cy7⊂T6* (gray dashed line). The vertical lines represent the stick spectrum of Cy7⊂*c*‐P6⋅T6*. b) Coupling (blue circles) and transfer rate (orange triangles) between the dye and bands of the nanoring at different wavelengths. c) Distribution of energy‐transfer times from Cy7 to the nanoring, calculated within second‐order perturbation theory in the dye‐nanoring coupling. The energy transfer takes less than 50 ps with 0.99 probability.

The Cy7⊂*c*‐P6⋅T6* complex is a much simpler molecular system than the LH1/RC supercomplex, but it provides a model to study and engineer the underlying mechanism behind the energy transfer in complex natural light‐harvesting systems. This work confirms that a special arrangement of dyes, similar to the LH1/RC complexes, with one dye at the center of a ring of other dyes, provides highly efficient energy transfer between the peripheral and central chromophores.

## Conflict of interest

The authors declare no conflict of interest.

## Supporting information

As a service to our authors and readers, this journal provides supporting information supplied by the authors. Such materials are peer reviewed and may be re‐organized for online delivery, but are not copy‐edited or typeset. Technical support issues arising from supporting information (other than missing files) should be addressed to the authors.

Supplementary
