# Supplementary material for: A Complex Comprising a Cyanine Dye Rotaxane and a Porphyrin Nanoring as a Model Light‐Harvesting System
Source: Angew Chem Int Ed Engl. 2020 Jul 23;59(38):16455–8. doi: 10.1002/anie.202006644 (PMC7540489; doi:10.1002/anie.202006644)
Supplement: Supplementary file 1 — Supplementary [file ANIE-59-16455-s001.pdf]

Angewandte Chemie
Eine Zeitschrift der Gesellschaft Deutscher Chemiker

Supporting Information

A Complex Comprising a Cyanine Dye Rotaxane and a Porphyrin Nanoring as a Model Light-Harvesting System**

Jiratheep Pruchyathamkorn, William J. Kendrick, Andrew T. Frawley, Andrea Mattioni, Felipe Caycedo-Soler, Susana F. Huelga, Martin B. Plenio, and Harry L. Anderson**

anie_202006644_sm_miscellaneous_information.pdf

Table of Contents .... page

1	General Experimental Procedures	S2
2	Synthetic Procedures	S4
3	Molecular Mechanics Calculations	S7
4	Characterization of New Compounds	S8
5	Spectra of New Compounds	S21
6	Photophysical Properties	S30
7	Theoretical Model	S34
8	References	S43

1 General Experimental Procedures

All reagents were purchased from commercial sources and used as received. Normal phase flash column chromatography was carried out using SiO_2 (60 Å pore size, 40–63 μm particle size, Aldrich, UK) as the stationary phase. Reverse phase flash column chromatography was carried out on a Biotage Isolera One automated flash system using a Biotage SNAP Ultra C18 column cartridge (particle size 25 μm).

NMR spectra were acquired on a Bruker AVB400, AVH400, AVG400, AVB500 or AV700 instrument at 298 K. NMR chemical shifts were reported in ppm relative to $SiMe_4$ (δ = 0) and were referenced internally with respect to residual solvent protons using the values reported by Fulmer et al.[1] Coupling constants are reported in Hz.

Electrospray mass spectrometry was carried out on a Waters Micromass LCT Premier XE spectrometer using 90:10 MeOH:H_2O (+0.1% formic acid) as the mobile phase. MALDI-TOF mass spectra were carried out by Waters MALDI Micro MX spectrometer using dithranol (1,8-dihydroxy-9,10-dihydroanthracen-9-one) as a matrix.

Molecular mechanics modeling was carried out with the MM+ force field in Hyperchem™ 8.0.10 (Hypercube Inc.) package.

Reverse phase HPLC was performed at 298 K using an Agilent 1100 Series system comprising an autosampler (G1313A), a vacuum degassing unit (G1379A), a quaternary pump (G1311A), a column oven (G1316A), a diode array detector (G1315B), and a fraction collector (G1364C). The instrument was operated using ChemStation software. For analytical HPLC an Agilent Eclipse XDB-C18 or XDB-C8 column (4.6 × 150 mm, 5 μm particle size) was used (flow rate = 1.0 mL/min). For semi-preparative HPLC an Agilent Eclipse XDB-C8 column (9.4 × 250 mm, 5 μm) was used (flow rate = 4.18 mL/min). Unless otherwise stated, a solvent system of H_2O/MeOH (with 0.1% trifluoroacetic acid) was used with a gradient shown in Table S1.

time / min	% H_2O	% MeOH
0	30	70
25	0	100
27	0	100
30	30	70

Table S1 Solvent gradient used for HPLC experiments in this work (percentages refer to volume/volume).

Optical spectroscopic measurements were conducted in HPLC grade solvents using quartz cuvettes (10 mm path length, Starna Scientific Ltd, UK). UV-vis-NIR absorption spectra were acquired on a Perkin Elmer Lambda 20 spectrometer at 298 K, with temperature control by a PTP-1 Peltier unit from Perkin Elmer. Fluorescence spectra were acquired at 298 K using an Edinburgh Instruments FS5 spectrofluorometer operating Fluoracle® software, and equipped with a xenon arc lamp (providing 230–1000 nm excitation range), a thermostatic sample holder (SC-20) and both an R13456 PMT detector (200–950 nm spectral coverage, Hamamatsu) and an InGaAs analogue NIR detector (850–1650 nm spectral coverage). Quantum yields (ϕ) were measured using a linear porphyrin hexamer as a reference (***l*-P6**, Φ_r = 28.0%; see **Section 6**).[2] Fluorescence lifetimes were measured in time-correlated single photon counting (TCSPC) mode using a picosecond pulsed diode laser (EPL-475) as the excitation source. Exponential reconvolution fits incorporating the measured instrument response function were used to extract fluorescence lifetimes.

Synthetic procedures for novel compounds are reported in **Section 2**, and the full spectra of novel compounds are provided in **Section 5**.

2 Synthetic Procedures

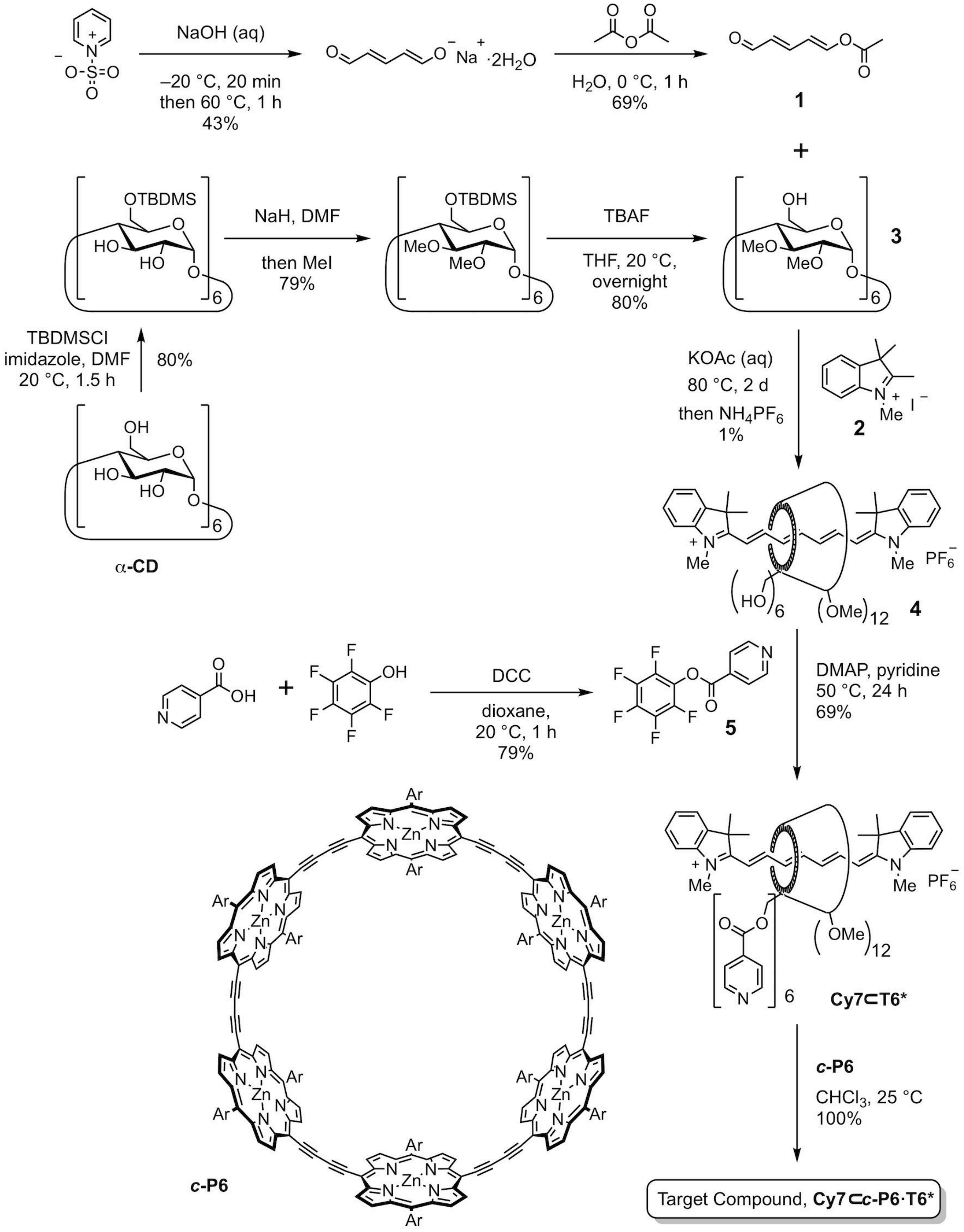

Scheme S1 Synthesis of the target compound **Cy7⊂*c*-P6·T6***.

Glutaconaldehyde acetate (**1**), per-2,3-di-O-methyl-α-cyclodextrin (**3**), isonicotinic acid pentafluorophenyl ester (**5**) and porphyrin nanoring (***c*-P6**) were synthesized using published procedures.[3-7]

Per-2,3-di-*O*-methyl-α-cyclodextrin encapsulated 1,1',3,3,3',3'-hexamethylindotricarbocyanine hexafluorophosphate (4)

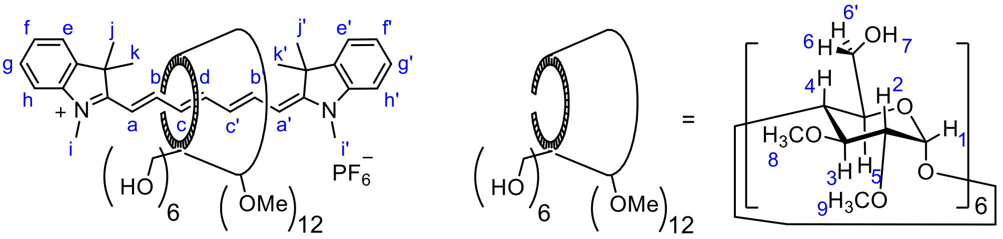

Per-2,3-di-*O*-methyl-α-cyclodextrin (**3**, 5.00 g, 4.38 mmol), tetramethyl indolium iodide (**2**, 5.30 g, 17.6 mmol) and acetylated glutaconaldehyde (**1**, 1.23 g, 8.80 mmol) were suspended in O_2-free potassium acetate solution (aq. 1.0 M, 50 mL) and stirred at 80 °C under N_2 for 48 h. After cooling, water (50 mL) was added. The resulting solution was extracted with dichloromethane (100 mL × 3) until the organic layer became colorless. The aqueous phase was purified by ion-exchange chromatography on a Sephadex C-25 column (8 cm × 20 cm), eluting with water and collecting the blue-green fraction. (The column was regenerated by a washing cycle of 1 M ammonium bicarbonate and water.) The solution was then concentrated and ammonium hexafluorophosphate (60 g) was added. The blue solution was evaporated to dryness under reduced pressure and purified further to remove unreacted per-2,3-di-*O*-methyl-α-cyclodextrin **3** by column chromatography (CH_2Cl_2/MeOH, 10/1, v/v). Blue fractions were combined and concentrated to obtain the product (68 mg, 1.0%) as a blue solid.

UV-Vis (MeOH) λ_{max} / nm (ε / M^{-1} cm^{-1}): 768 (1.80 × 10^5).

^{1}H NMR (500 MHz, d_6-DMSO): δ = 7.78 (t, J = 12.6 Hz, 1H, H_b), 7.65 (d, J = 7.9 Hz, 1H, H_e), 7.58 (d, J = 8.1 Hz, 1H, H_h), 7.50 (t, J = 7.8 Hz, 1H, $H_{b'}$), 7.48 (m, 1H, H_g), 7.40 (d, J = 8.0 Hz, 1H, $H_{e'}$), 7.38 (t, J = 7.4 Hz, 1H, H_f), 7.32 (t, J = 7.7 Hz, 1H, $H_{g'}$), 7.22 (d, J = 8.0 Hz, 1H, $H_{h'}$), 7.09 (t, J = 7.2 Hz, 1H, $H_{f'}$), 6.99 (t, J = 12.6 Hz, 1H, H_d), 6.77 (t, J = 12.5 Hz, 1H, H_c), 6.68 (t, J = 12.7 Hz, 1H, $H_{c'}$), 6.58 (d, J = 13.9 Hz, 1H, H_a), 6.04 (d, J = 12.7 Hz, 1H, $H_{a'}$), 5.05 (d, J = 3.6 Hz, 6H, H_1), 4.58 (t, J = 5.6 Hz, 6H, H_7), 3.85 (m, 6H, H_6), 3.73 (s, 3H, H_i), 3.67 (m, 6H, $H_{6'}$), 3.66 (m, 6H, H_5), 3.57 (s, 3H, $H_{i'}$), 3.52 (m, 6H, H_4), 3.42 (s, 18H, H_8), 3.37 (m, 6H, H_3), 3.37 (s, 18H, H_9), 3.02 (dd, J = 9.9 Hz, 3.4 Hz, 6H, H_2), 1.75 (s, 3H, $H_{j'}$), 1.69 (s, 3H, $H_{k'}$), 1.66 (s, 3H, H_j), 1.62 (s, 3H, H_k).

^{13}C NMR (500 MHz, d_6-DMSO): δ = 175.8, 167.1, 151.2, 148.9, 143.7, 143.0, 142.1, 139.7, 128.4, 128.2, 127.8, 126.6, 126.3, 125.6, 123.2, 122.2, 121.8, 112.2, 109.6, 108.3, 101.7, 98.6, 81.8, 81.5, 80.8, 71.9, 60.2, 60.1, 57.0, 50.3, 48.1, 32.0, 30.6, 29.2, 29.0, 26.6, 26.1.

MS: m/z 1550.6 ($[M]^+$, $C_{77}H_{117}N_2O_{30}$ requires 1550.7).

Per-2,3-di-*O*-methyl-6-*O*-(*para*-pyridinyl)carboxyl-α-cyclodextrin encapsulated 1,1',3,3,3',3'-hexamethylindotricarbocyanine hexafluorophosphate (Cy7⊂T6*)

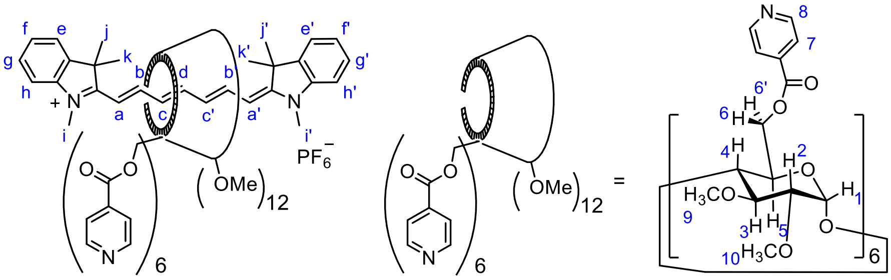

Compound **4** (18.0 mg, 0.0106 mmol), 4-dimethylaminopyridine (DMAP, 92.0 mg, 0.742 mmol) and isonicotinic acid pentafluorophenyl ester (**5**, 305 mg, 1.06 mmol) were dried under vacuum for 2 h. Anhydrous pyridine (5 mL) was added, and the mixture was stirred at 50 °C for 18 h under N_2 atmosphere, at which point MS analysis indicated the completion of the reaction. The reaction mixture was cooled and the solvent was evaporated under vacuum. The residue was dissolved in chloroform (25 mL) and washed with water (3 × 25 mL). The solution was evaporated and dried under reduced pressure. Reverse phase column chromatography (Biotage SNAP Ultra C18 12 g, MeOH (0.1% TFA)/water (0.1% TFA), 7/3, *v*/*v*) of the residue gave the product as blue solid (17 mg, 69%), R_f = 0.15 (reverse phase TLC).

UV-Vis (MeOH) λ_{max} / nm (ε / M^{-1} cm^{-1}): 779 (1.53 × 10^5).

^{1}H NMR (400 MHz, d_6-DMSO): δ = 8.71 (d, *J* = 5.2 Hz, 12H, H_8), 7.77 (d, *J* = 6.1 Hz, 12H, H_7), 7.72 – 7.61 (m, 4H, $H_{b,\ b',\ e,\ h}$), 7.58 (d, *J* = 7.6 Hz, 1H, $H_{e'}$), 7.55 (d, *J* = 7.4 Hz, 1H, $H_{f'}$), 7.49 – 7.35 (m, 3H, $H_{f,\ g,\ h'}$), 7.21 (t, *J* = 7.0 Hz, 1H, $H_{g'}$), 6.97 (t, *J* = 12.9 Hz, 1H, H_d), 6.78 (t, *J* = 12.6 Hz, 1H, $H_{c'}$), 6.40 (t, *J* = 12.6 Hz, 1H, H_c), 6.35 (d, *J* = 12.9 Hz, 1H, H_a), 6.23 (d, *J* = 12.9 Hz, 1H, $H_{a'}$), 5.23 (d, *J* = 3.5 Hz, 6H, H_1), 4.80 – 4.60 (m, 12H, $H_{6,\ 6'}$), 4.04 (d, *J* = 9.7 Hz, 6H, H_5), 3.91 (t, *J* = 9.0 Hz, 6H, H_4), 3.79 (s, 3H, H_i), 3.70 (s, 3H, $H_{i'}$), 3.49 (s, 18H, H_9), 3.46 (m, 6H, H_3), 3.41 (s, 18H, H_{10}), 3.23 (dd, *J* = 9.9, 3.3 Hz, 6H, H_2), 1.86 (s, 3H, $H_{j'}$), 1.80 (s, 3H, $H_{k'}$), 1.69 (s, 3H, H_j), 1.67 (s, 3H, H_k).

ESI MS: *m*/*z* 2183.5 ($[M]^+$, $C_{113}H_{135}N_8O_{36}$ requires 2181.3).

The Target Compound Cy7$\subset$*c*-P6·T6*

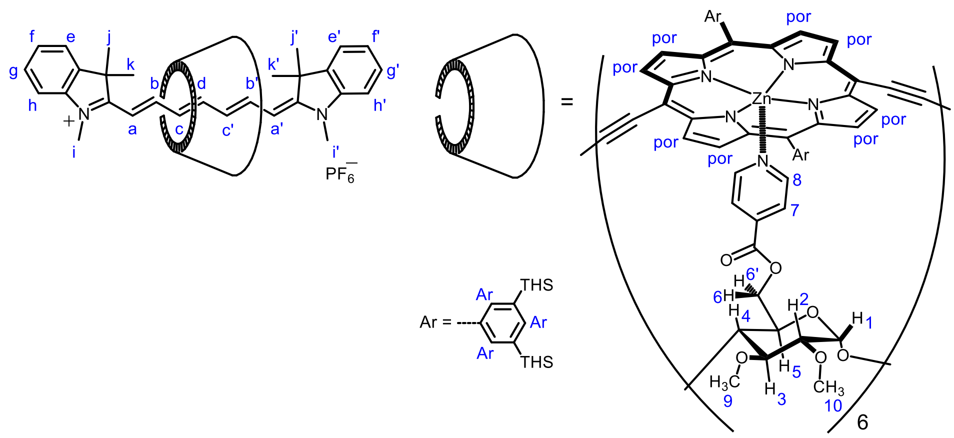

Cy7$\subset$T6* (6.0 mg, 2.6 µmol) and ***c*-P6** (6.0 mg, 0.56 µmol) were mixed in $CHCl_3$ (1 mL) and the solution was passed through a short size-exclusion column on a Bio-Beads S-X3 support using toluene as an eluent. A dark-brown band was collected and dried to give the product as a dark-brown solid (7.3 mg, 100%).

UV-Vis (CH_2Cl_2) λ_{max} / nm (ε / M^{-1} cm^{-1}): 482 (5.71×10^5), 777 (5.25×10^5), 805 (5.01×10^5), 849 (3.26×10^5).

^{1}H NMR (700 MHz, CD_2Cl_2): δ = 9.65 (d, *J* = 4.2 Hz, 6H, H_{por}), 9.61 (d, *J* = 4.2 Hz, 6H, H_{por}), 9.56 (d, *J* = 4.1 Hz, 6H, H_{por}), 9.50 (d, *J* = 4.2 Hz, 6H, H_{por}), 8.86 (d, *J* = 4.3 Hz, 6H, H_{por}), 8.82 (d, *J* = 4.2 Hz, 6H, H_{por}), 8.78 (d, *J* = 4.1 Hz, 6H, H_{por}), 8.73 (d, *J* = 4.2 Hz, 6H, H_{por}), 8.52 (s, 6H, H_{Ar}), 8.37 (s, 6H, H_{Ar}), 8.05 (s, 6H, H_{Ar}), 8.00 (s, 6H, H_{Ar}), 7.97 (s, 6H, H_{Ar}), 7.90 (s, 6H, H_{Ar}), 7.36 (m, 1H, $H_{g'}$), 7.19 (m, 1H, $H_{f'}$), 7.11 (d, *J* = 6.1 Hz, 1H, $H_{e'}$), 7.01 (d, *J* = 7.5 Hz, 1H, $H_{h'}$), 6.97 (m, 1H, $H_{b'}$), 6.93 (m, 1H, H_g), 6.79 (m, 1H, H_f), 6.62 (d, *J* = 6.3 Hz, 1H, H_e), 6.42 (d, *J* = 7.6 Hz, 1H, H_h), 6.29 (t, *J* = 13.0 Hz, 1H, H_b), 5.94 (t, *J* = 13.6 Hz, 1H, H_d), 5.78 (t, *J* = 12.2 Hz, 1H, $H_{c'}$), 5.56 (m, 1H, $H_{a'}$), 5.54 (d, *J* = 7.3 Hz, 12H, H_7), 5.04 (d, *J* = 13.6 Hz, 1H, H_a), 4.98 (t, *J* = 11.1 Hz, 1H, H_c), 3.56 (d, *J* = 7.3 Hz, 6H, H_1), 3.25 (d, *J* = 8.3 Hz, 6H, $H_{6'}$), 3.18 (s, 3H, $H_{i'}$), 3.04 (s, 18H, H_9), 2.95 (d, *J* = 10.5 Hz, 6H, H_6), 2.88 (s, 18H, H_{10}), 2.78 (m, 6H, H_5), 2.71 (s, 3H, H_i), 2.60 (d, *J* = 7.1 Hz, 12H, H_8), 2.53 (t, *J* = 9.1 Hz, 6H, H_3), 2.48 (d, *J* = 9.3 Hz, 6H, H_4), 2.20 (dd, *J* = 9.9, 3.9 Hz, 6H, H_2), 1.61 – 1.16 (m, 576H, H_{THS}), 1.32 (s, 3H, $H_{j'}$), 1.29 (s, 3H, $H_{k'}$), 1.05 – 0.80 (m, 360H, H_{THS}), 0.70 (s, 3H, H_j), 0.67 (s, 3H, H_k).

MALDI-TOF MS: *m/z* 12,391.2 ($[M]^+$, $C_{761}H_{1155}N_{32}O_{36}Si_{24}Zn_6$ requires 12,394.9).

3 Molecular Mechanics Calculations

Molecular mechanics simulations (Hyperchem™ software, MM+ force field) were carried out to predict the structures of the target compound **Cy7$\subset$*c*-P6·T6***. Two-low energy geometries, conformations **A** and **B**, were found, as discussed in the main text (Fig. 1). The energies of the two conformers calculated after geometry optimization very similar (389.98 kJ mol^{-1} for conformation **A** and 390.66 kJ mol^{-1} for conformation **B**).

4 Characterization of New Compounds

Rotaxane 4

The NMR and MS characterization of **4** are discussed in detail here; see **Section 5** for the full set of spectra.

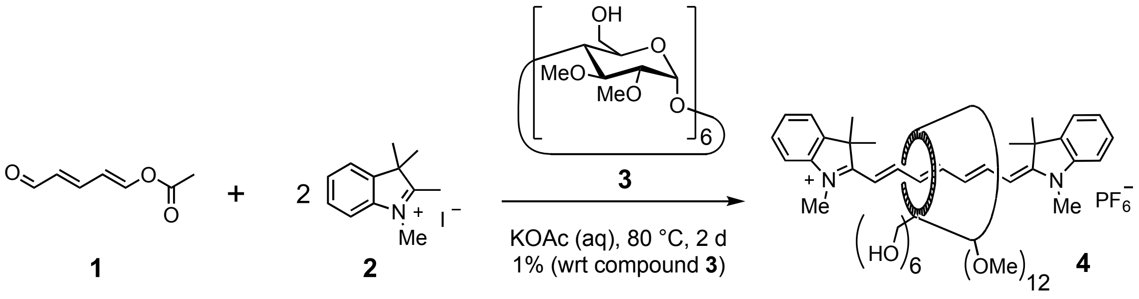

Scheme S2 Synthesis of rotaxane **4**.

Rotaxane formation was clearly demonstrated by the ^{1}H-NMR spectra of compound **4**. As compound **3** is chiral, the cyanine dye thread becomes desymmetrized upon encapsulation. Unlike in the **Cy7** free dye, all proton NMR signals on the thread of the rotaxane **4** have different chemical shifts. The protons inside the cyclodextrin cavity ($H_{3,5}$) show many NOE correlations to the **Cy7** bridge, whereas the outside protons ($H_{1,2,4}$) do not (**Fig. S4**). The NOE correlations between $H_{i,j,k}$ and $H_{6,6',7}$, confirm that the narrow primary side of compound **3** is at one end of the backbone, and the correlations between $H_{i',j',k',a'}$ and $H_{8,9}$ confirm that the wider rim is facing the other end. The orientation of the cyclodextrin on the thread of the rotaxane is shown in **Fig. S1**. HSQC and HMBC spectra were used to assign carbon chemical shifts for each carbon atom of the rotaxane **4** (see **Section 3** for the assignment).

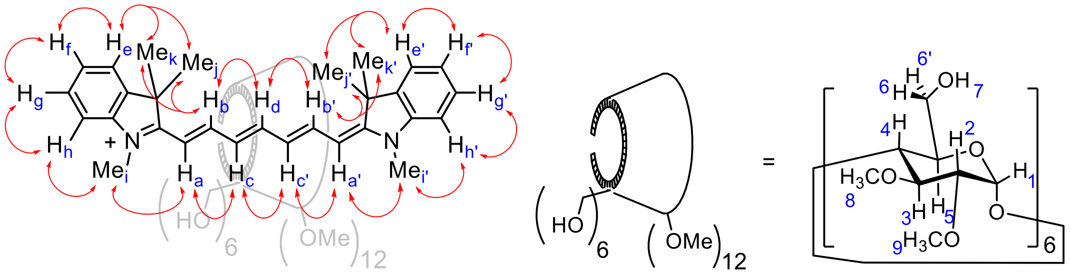

Fig. S1 Structure of the rotaxane **4** with NOE correlations between **Cy7** protons (red arrows).

COSY

H	1	2	3	4	5	6, 6′	7	8	9	a	b	c	d	a'	b'	c'	i	j, k	i'	j', k′
1	—	green	gray	gray	gray	gray	gray	gray	gray	gray	gray	gray	gray	gray	gray	gray	gray	gray	gray	gray
2	green	—	green	gray	gray	gray	gray	gray	gray	gray	gray	gray	gray	gray	gray	gray	gray	gray	gray	gray
3	gray	gray	—	green	gray	gray	gray	gray	gray	gray	gray	gray	gray	gray	gray	gray	gray	gray	gray	gray
4	green	green	gray	—	green	gray	gray	gray	gray	gray	gray	gray	gray	gray	gray	gray	gray	gray	gray	gray
5	gray	gray	green	gray	—	green	gray	gray	gray	gray	gray	gray	gray	gray	gray	gray	gray	gray	gray	gray
6, 6′	yellow	yellow	gray	green	green	—	green	gray	gray	gray	gray	gray	gray	gray	gray	gray	gray	gray	gray	gray
7	yellow	yellow	gray	green	yellow	green	—	gray	gray	gray	gray	gray	gray	gray	gray	gray	gray	gray	gray	gray
8	yellow	green	gray	green	gray	green	gray	—	gray	gray	gray	gray	gray	gray	gray	gray	gray	gray	gray	gray
9	green	green	gray	yellow	gray	yellow	gray	green	—	gray	gray	gray	gray	gray	gray	gray	gray	gray	gray	gray
a	gray	gray	gray	gray	green	gray	gray	gray	gray	—	green	gray	gray	gray	gray	gray	gray	gray	gray	gray
b	gray	gray	yellow	gray	green	yellow	gray	gray	gray	gray	—	green	gray	gray	gray	gray	gray	gray	gray	gray
c	gray	gray	yellow	gray	green	yellow	gray	gray	gray	green	gray	—	green	gray	gray	gray	gray	gray	gray	gray
d	gray	gray	yellow	gray	green	yellow	gray	gray	gray	gray	green	gray	—	gray	gray	green	gray	gray	gray	gray
a'	gray	gray	gray	gray	yellow	gray	gray	green	green	gray	gray	gray	gray	—	green	gray	gray	gray	gray	gray
b'	gray	gray	yellow	gray	green	gray	gray	yellow	yellow	gray	gray	gray	green	gray	—	green	gray	gray	gray	gray
c'	gray	gray	yellow	gray	green	yellow	gray	gray	gray	gray	gray	green	gray	green	gray	—	gray	gray	gray	gray
i	gray	gray	gray	gray	gray	yellow	yellow	gray	gray	green	gray	yellow	gray	gray	gray	gray	—	gray	gray	gray
j, k	gray	gray	gray	gray	gray	yellow	yellow	gray	gray	gray	green	gray	gray	gray	gray	gray	gray	—	gray	gray
i'	gray	gray	gray	gray	gray	gray	gray	green	yellow	gray	gray	gray	gray	green	gray	gray	gray	gray	—	gray
j', k′	gray	gray	gray	gray	gray	gray	gray	green	green	gray	gray	gray	gray	gray	green	gray	gray	gray	gray	—

NOESY

Table S2 Summary of the COSY and NOESY correlations (d_6-DMSO, 500 MHz) between protons of the rotaxane **4** (green = strong, yellow = weak, and gray = no correlation). The cyclodextrin protons are labeled in numbers, and the **Cy7** dye protons are labeled alphabetically according to **Fig. S1**. The aromatic protons of the dye were ignored. Full COSY and NOESY spectra can be found in the **Section 5**.

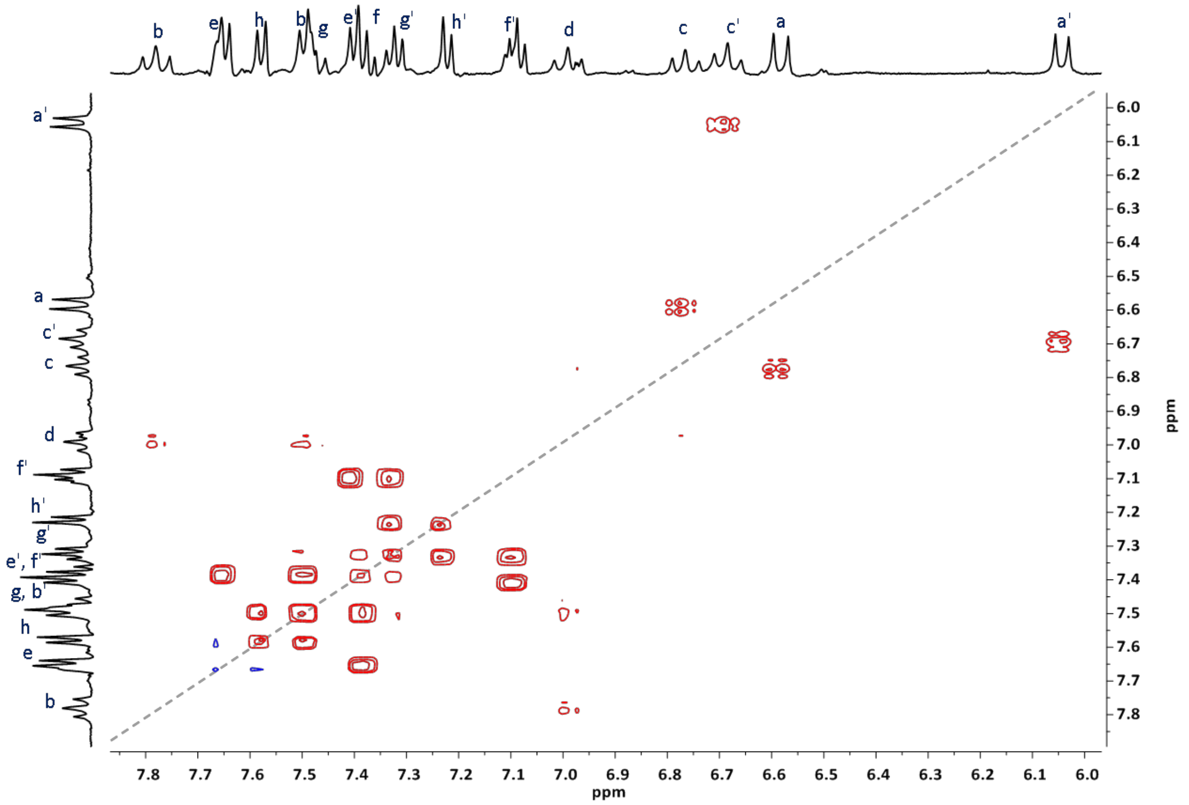

Fig. S2 Region of the 2D $^1H/^1H$ NOESY spectrum (d_6-DMSO, 500 MHz) of the rotaxane **4** showing the correlations between the dye protons. The diagonal peaks have been suppressed for simplification.

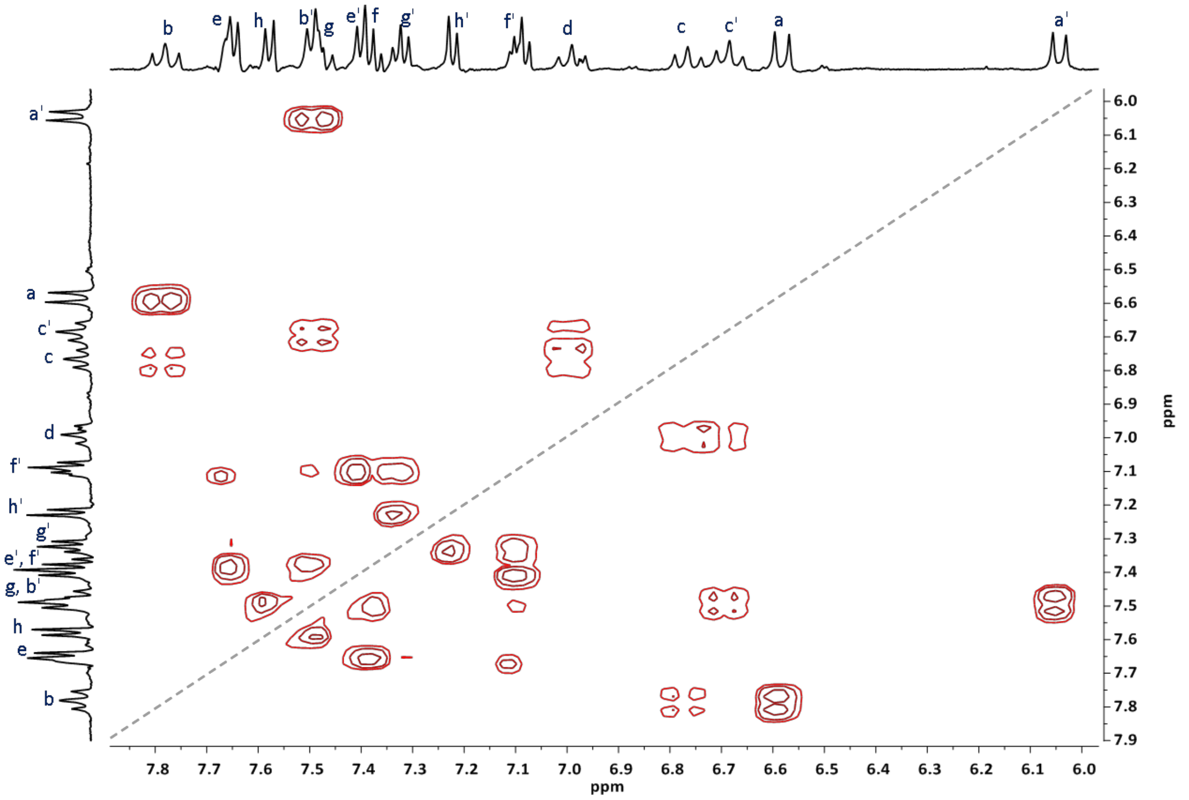

Fig. S3 Region of the 2D $^1H/^1H$ COSY spectrum (d_6-DMSO, 500 MHz) of the rotaxane **4** showing the correlations between the dye protons. The diagonal peaks have been suppressed for simplification.

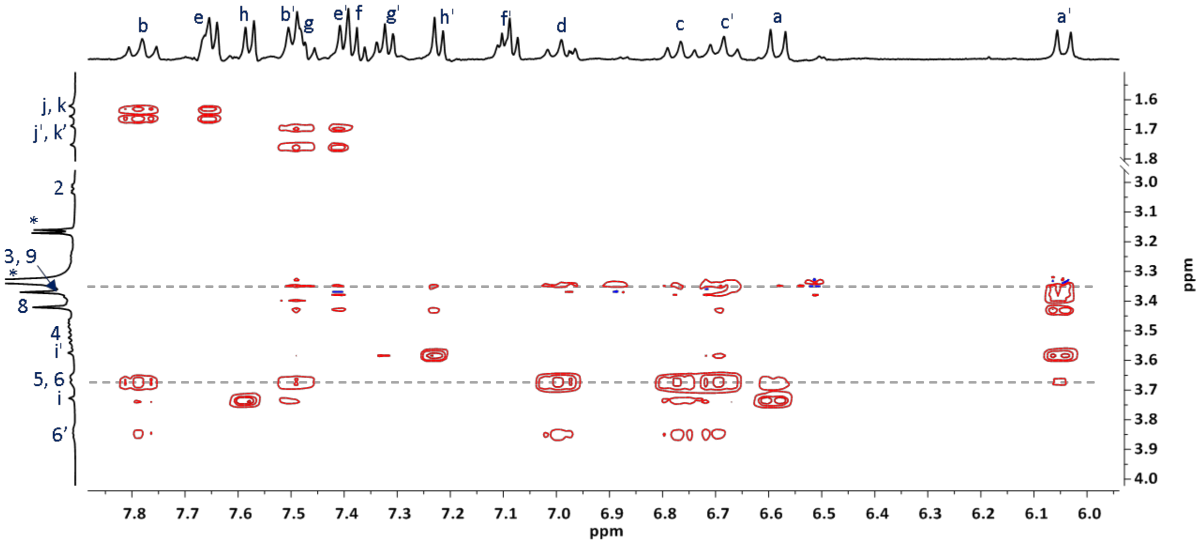

Fig. S4 Region of the 2D $^1H/^1H$ NOESY spectrum (d_6-DMSO, 500 MHz) of the rotaxane **4** showing the correlations between the dye backbone and the functionalized cyclodextrin. The correlations between the protons inside the cyclodextrin cavity $H_{3,5}$ and the dye protons are marked by gray dashed lines. The spectrum contains the solvent peak and impurities (water and MeOH) labeled with "*".

Mass spectrometry confirmed formation of the rotaxane; the expected molecular ion is observed (m/z 1550.5 for rotaxane **4**, **Section 5**). UV-vis-NIR absorption spectra (**Fig. S5c**) indicate a red shift upon the rotaxane formation (λ_{max} = 768 nm for the rotaxane **4**) compared to the **Cy7** free dye (λ_{max} = 740 nm), as reported previously for **Cy7⊂α-CD**.[9]

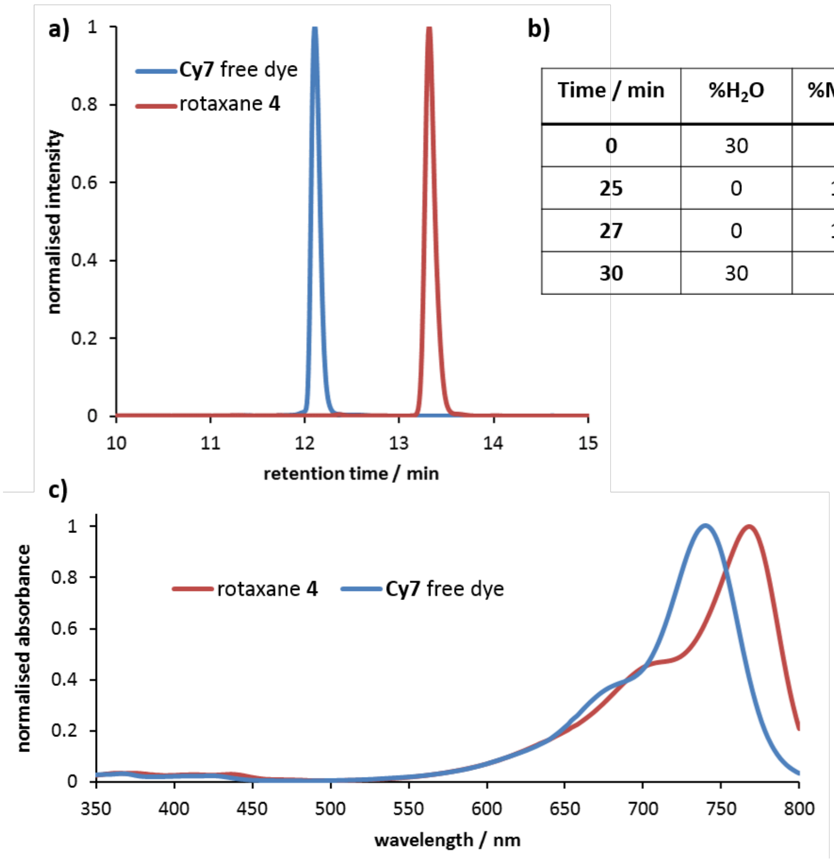

Time / min	%H_2O	%MeOH
**0**	30	70
**25**	0	100
**27**	0	100
**30**	30	70

Fig. S5 a) HPLC traces for the rotaxane **4** (blue) and **Cy7** free dye using semi-preparative HPLC (Agilent Eclipse XDB (9.4 × 250 mm, 5 μm), flow rate of 4.18 mL/min), **b**) gradient elution of the HPLC solvent system of H_2O / CH_3OH (with 0.1% trifluoroacetic acid), and **c**) absorption spectra of the rotaxane **4** and **Cy7** free dye measured during the HPLC analysis.

Rotaxane Template Cy7⊂T6*

The NMR and MS characterization of **Cy7⊂T6*** are discussed in detail here; see **Section 5** for the full set of spectra.

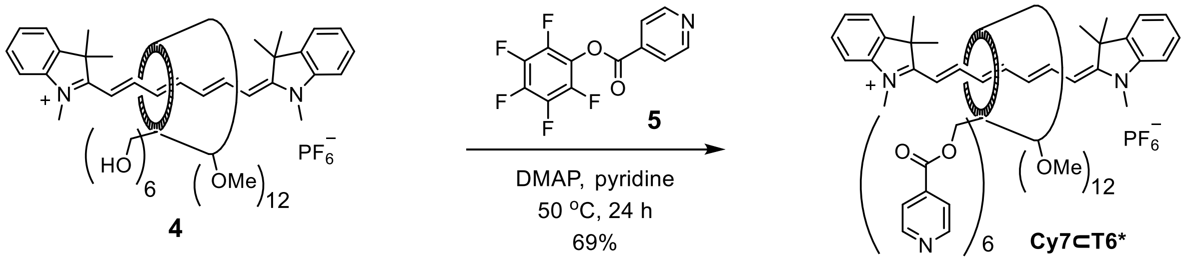

Scheme S3 Synthesis of **Cy7⊂T6***.

NMR spectra of **Cy7⊂T6*** were acquired in d_6-DMSO. All proton chemical shifts were fully assigned by interpretation of COSY and NOESY spectra.

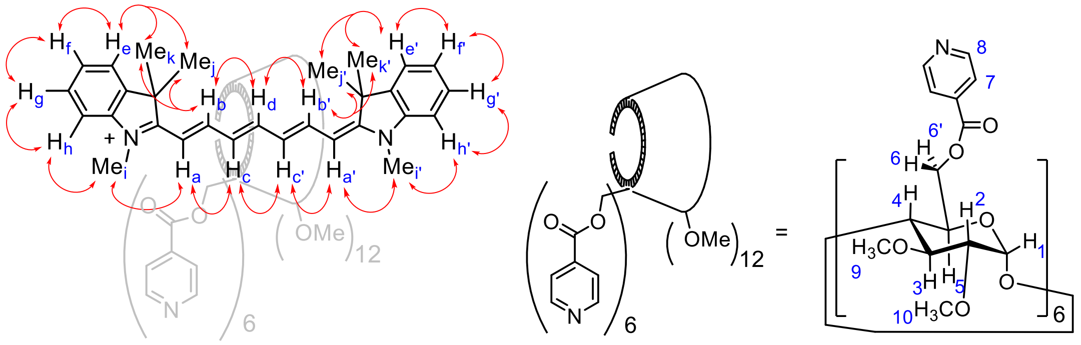

Fig. S6 Simplified structure of **Cy7⊂T6*** with NOE correlations between **Cy7** protons (red arrows).

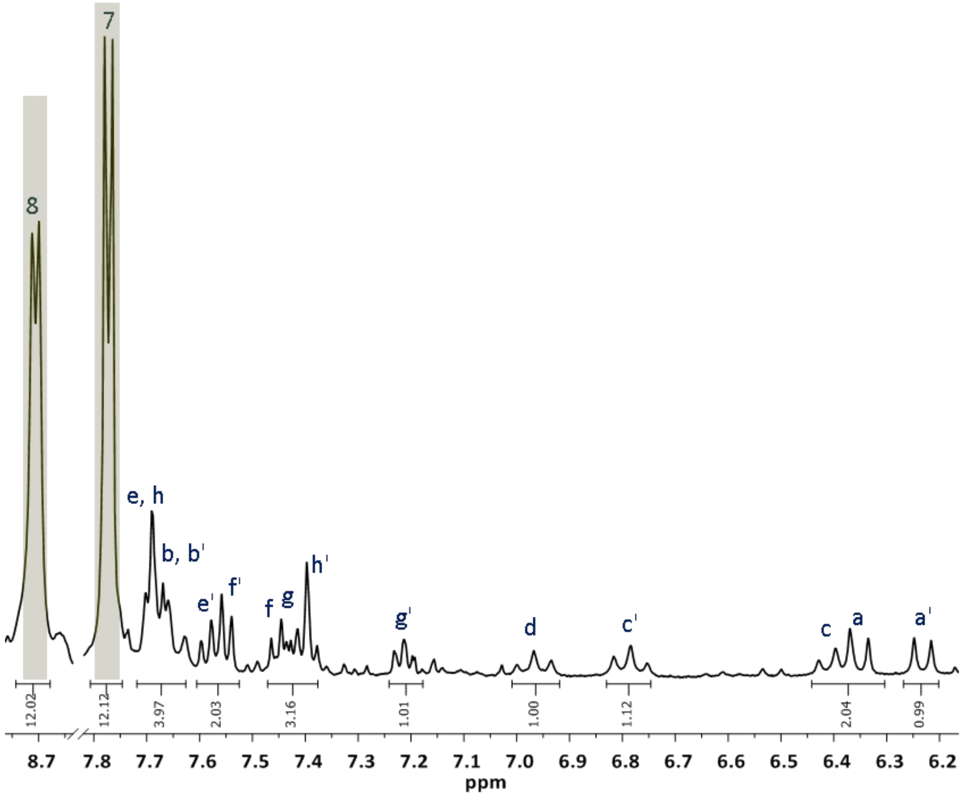

Fig. S7 Section of the ^{1}H-NMR spectrum (d_6-DMSO, 400 MHz) of **Cy7⊂T6*** showing the aromatic region. All protons of **Cy7⊂T6***are assigned in blue with the labels corresponded to **Fig. S6**. The pyridyl proton signals ($H_{7,8}$) on **T6*** are highlighted in yellow.

The pyridyl proton signals (H_7 and H_8) clearly demonstrate the formation of **Cy7$\subset$T6*** (**Fig. S7**). Each peak corresponds to 12 protons, and therefore the intensities of these peaks are high when compared to other **Cy7** signals within the same region.

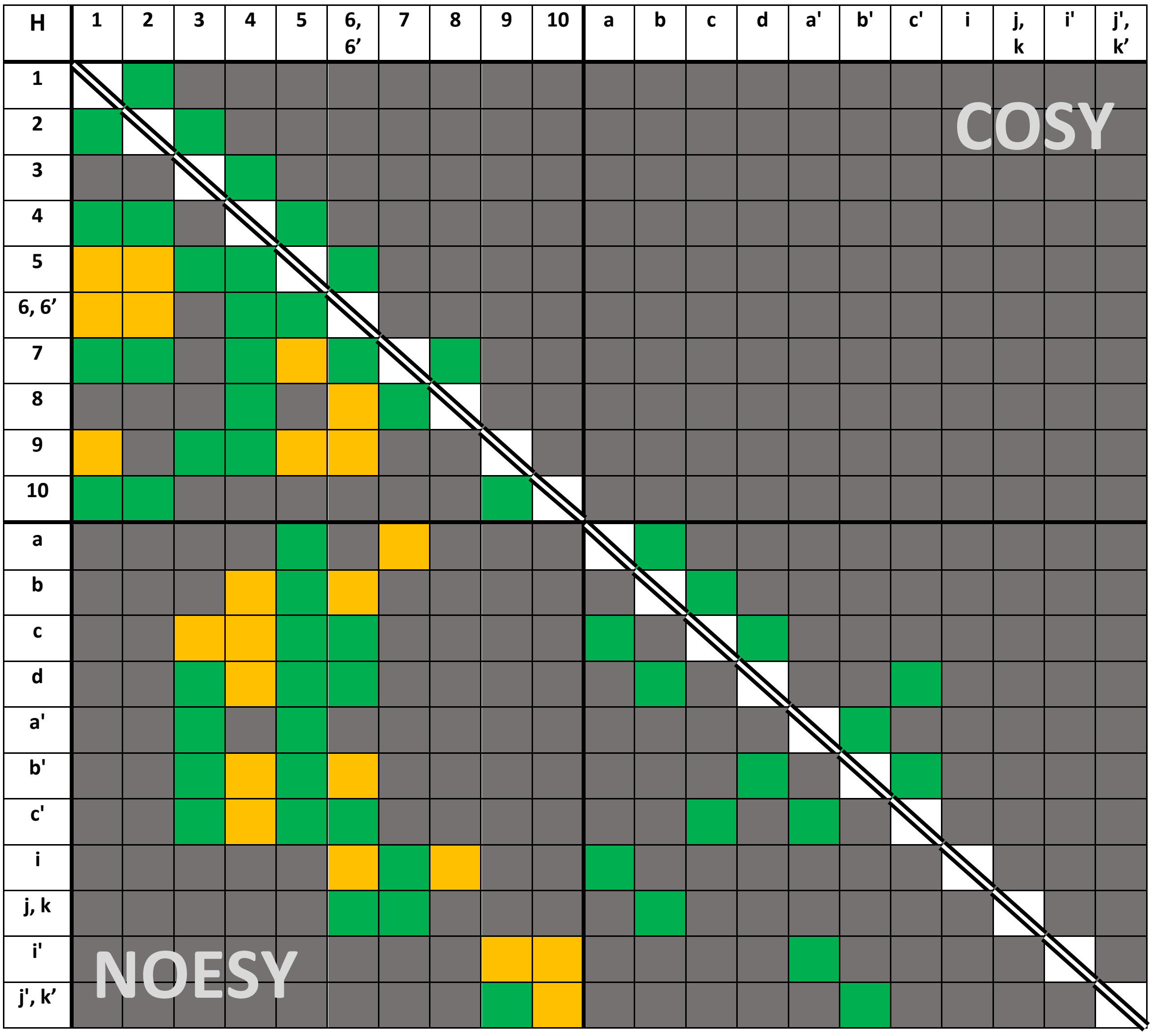

Table S3 Summary of the COSY and NOESY correlations (d_6-DMSO, 400 MHz) between protons of **Cy7$\subset$T6*** (green = strong, yellow = weak, and gray = no correlation). The **T6*** protons are labeled in numbers, and the **Cy7** dye protons are labeled alphabetically according to **Fig. S6**. The aromatic protons of the dye were ignored. Full COSY and NOESY spectra can be found in **Section 5**.

Due to the chirality of the cyclodextrin species (**T6***), all proton NMR signals on the thread of **Cy7$\subset$T6*** have different chemical shifts. The COSY and NOESY correlations are summarized (**Table S3**).

NOE correlations between the dye backbone and the functionalized cyclodextrin (**T6***) confirm the rotaxane structure (**Fig. S8**). The correlations between $H_{i,j,k}$ and $H_{6,6',7}$, show that the narrow rim of **T6*** is at one end of the backbone, and the correlations between $H_{i',j',k',a'}$ and $H_{9,10}$ show that the wider rim is facing the other end. Strong correlations between $H_{c',d,c}$ and $H_{6,6'}$ suggest that, on average, the narrow rim of the **T6*** is positioned in the middle of the dye backbone, with one end of the polymethine bridge exposed to the outer environment (**Fig. S9**). This is also supported by the correlation between H_a and H_7, and between Me_i and H_8.

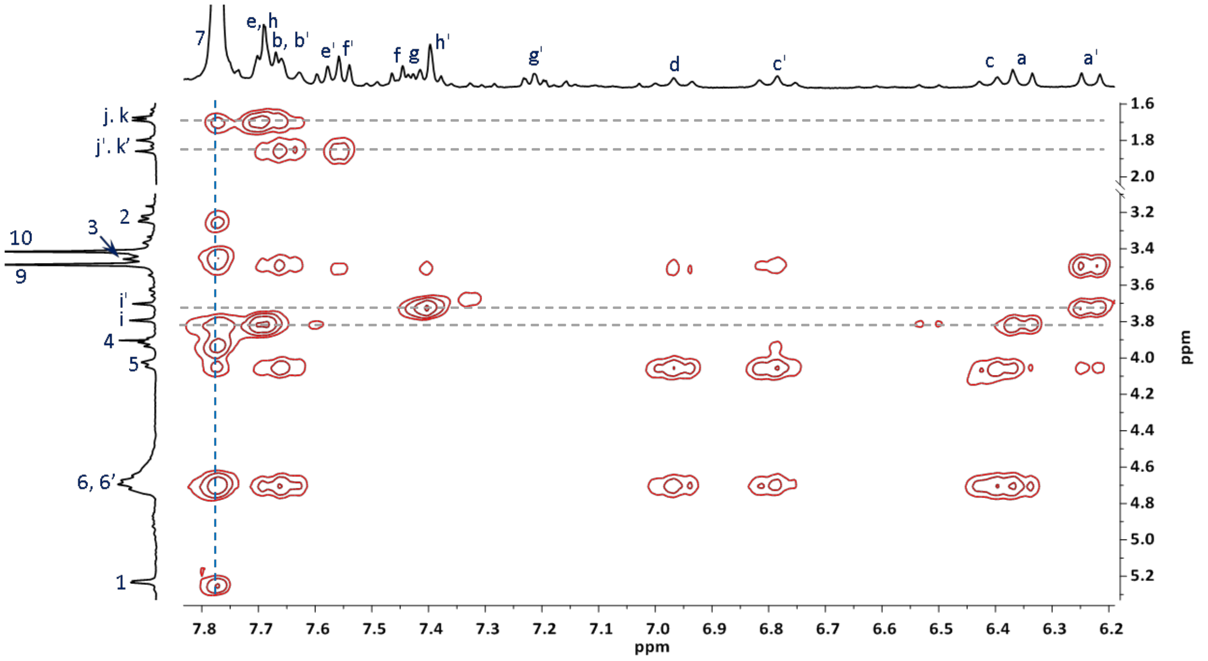

Fig. S8 Region of the 2D $^1H/^1H$ NOESY spectrum (d_6-DMSO, 400 MHz) of **Cy7⊂T6*** showing the correlations between the dye backbone and the functionalized cyclodextrin (**T6***). The correlations between $Me_{i,j,k,i',j',k'}$ and the dye backbone are also shown, and marked by gray dashed lines. The correlations between pyridyl proton H_7 and **T6*** are marked on a blue dashed line.

The predicted position of **T6*** on the dye backbone is consistent with the geometry of the cyclodextrin, as it would avoid the steric clashes between the narrow rim and the stopper. Some similar correlations can also be seen on the NOESY spectrum for rotaxane **4**, although much weaker signals are observed in this case. This could mean that the introduction of the pyridyl groups on the cyclodextrin might increase the steric bulk of the narrow rim and make the position of **T6*** presented in **Fig. S9** more favorable, compared to the case where the cyclodextrin species is situated in the middle of the dye backbone.

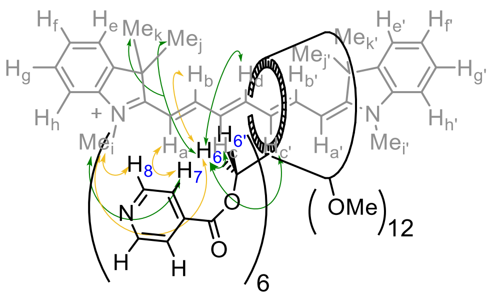

Fig. S9 Simplified structure of **Cy7⊂T6*** showing the position of **T6*** (black) on the dye backbone (gray), as suggested by the NOESY correlations (**Table S3**). The arrows show some important NOESY correlations to confirm the position of **T6*** (green = strong and yellow = weak correlation).

A red shift is observed by comparing the UV-vis-NIR absorption spectra of **Cy7⊂T6*** with the **Cy7** free dye (**Fig. S10**).

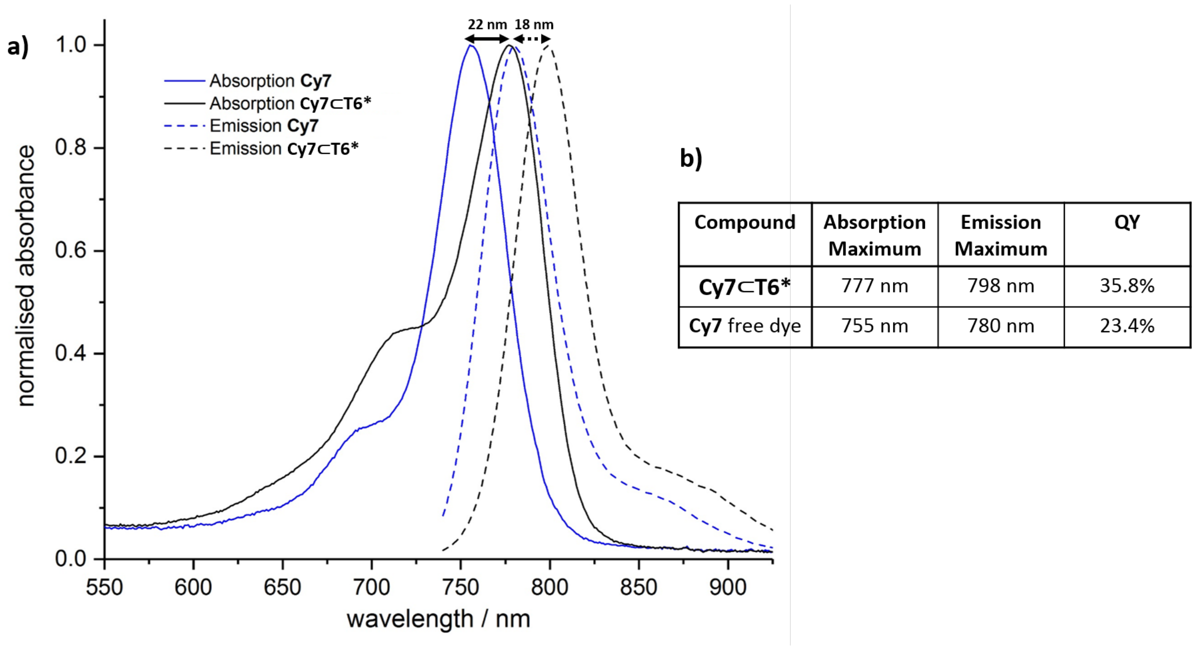

b)

Compound	Absorption Maximum	Emission Maximum	QY
**Cy7⊂T6***	777 nm	798 nm	35.8%
**Cy7** free dye	755 nm	780 nm	23.4%

Fig. S10 a) Uv-vis-NIR absorption (solid line) and emission (dashed line) of **Cy7⊂T6*** (black) and the **Cy7** free dye (blue) at 298 K in CH_2Cl_2. The spectra are normalized to compare the wavelength difference between the maxima, and **b**) λ_{max} and fluorescence quantum yields (QY, Φ_f) of each compound.

Detailed Characterization of Target Compound Cy7⊂*c*-P6·T6*

The NMR and MS characterization of the target compound are discussed in detail here; see **Section 5** for the full set of spectra.

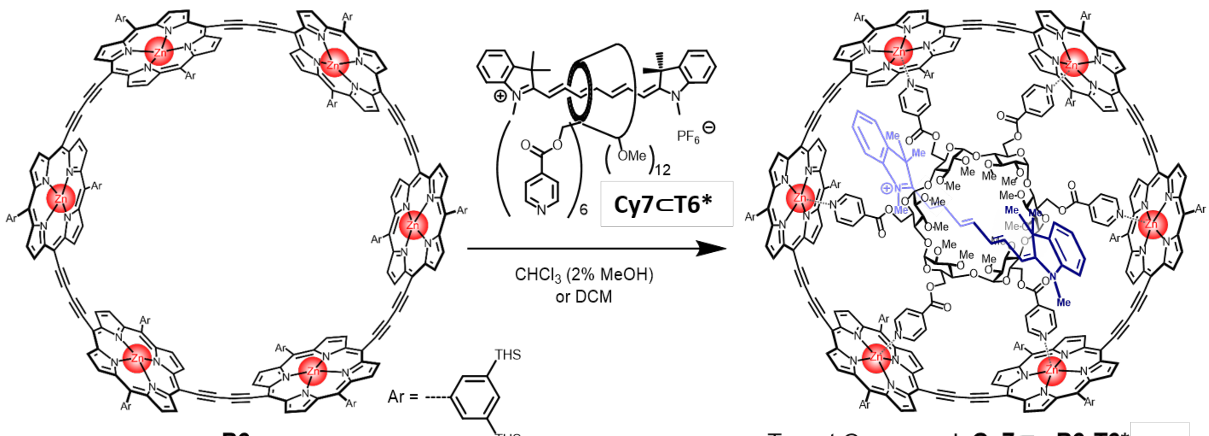

c*-P6** Target Compound, **Cy7⊂*c*-P6·T6

Scheme S4 Synthesis of the target compound **Cy7⊂*c*-P6·T6*** from **Cy7⊂T6*** and the porphyrin nanoring ***c*-P6** with THS solubilizing groups.

The target compound **Cy7⊂*c*-P6·T6*** is soluble in organic solvents. From the ^{1}H-NMR spectrum (**Fig. S11**), eight doublet β-porphyrin protons (H_{por}) have different chemical shifts, demonstrating that **Cy7⊂T6*** is bound to the ***c*-P6** ring. The protons on **T6*** are shielded by the aromatic ring current of the ***c*-P6**. NOESY correlations between the *Ar/por* signals and proton 8 (template α-proton) provide more evidence for template binding (**Fig. S16**). Proton 10 (cylodextrin methyl ether) also shows a correlation to the *ortho*-aryl protons.

All proton chemical shifts were fully assigned by interpretation of COSY and NOESY spectra (**Figs S12** and **S13**, **Table S4**). Comparing to the ^{1}H NMR spectra of **Cy7⊂T6***, most protons on the **Cy7** dye and **T6*** unit of **Cy7⊂*c*-P6·T6*** appear at the expected chemical shifts. The COSY correlation between H_5 and $H_{6,6'}$ of **Cy7⊂*c*-P6·T6*** cannot be seen (**Fig. S12b**), possibly due to the dihedral angle of the protons approaching 90°, where the magnitude of the $^{3}J_{H,H}$ coupling constant is nearly zero as predicted by the Karplus equation. This observation is not seen in the case of **Cy7⊂T6***, and suggests that binding to the ***c*-P6** ring might affect the dihedral angle of between these protons. Despite the lack of the information from the COSY spectrum, $H_{5,6,6'}$ could be assigned by the strong NOE correlations (**Fig. S13b**). The other **Cy7** and **T6*** protons of **Cy7⊂*c*-P6·T6*** show COSY and NOESY correlations as expected.

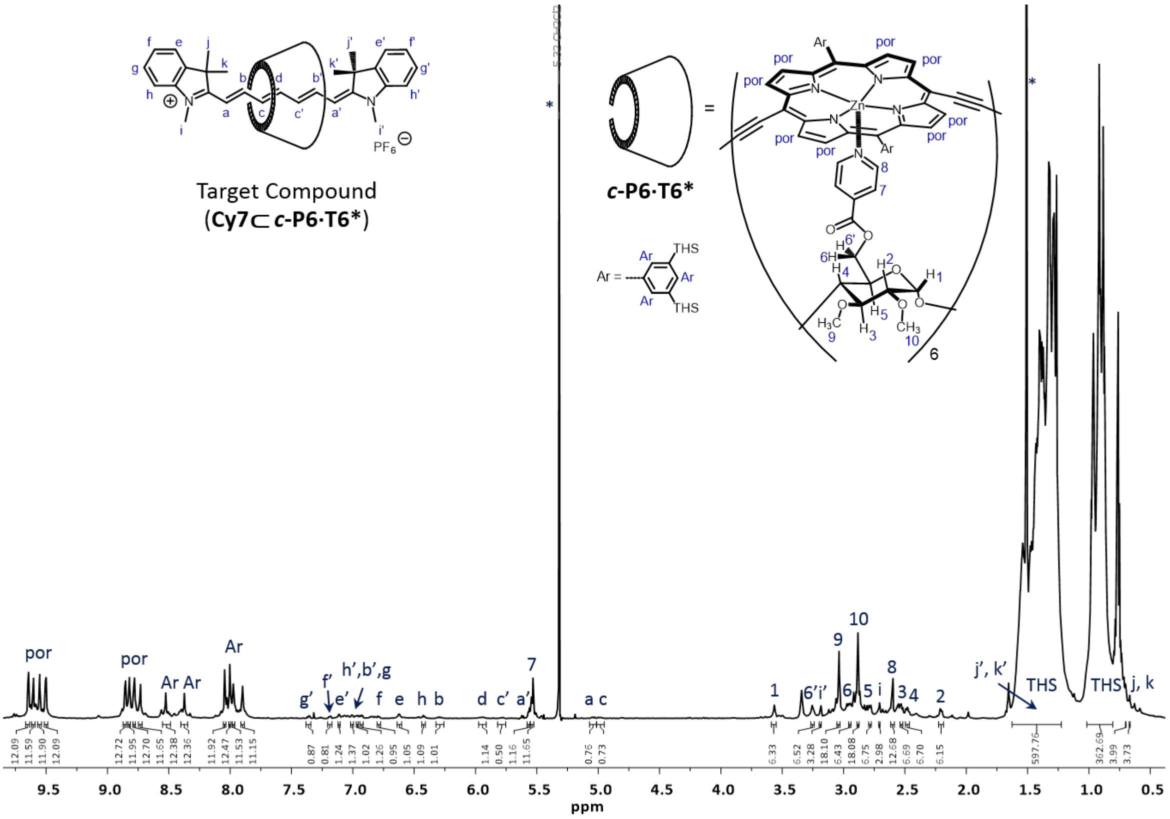

Fig. S11 ^{1}H-NMR spectrum (CD_2Cl_2, 700 MHz) of the target compound **Cy7⊂*c*-P6·T6***. The pyridyl proton signals are labeled in blue, and the solvent peaks ($CDHCl_2$ and water) is labeled with '*'.

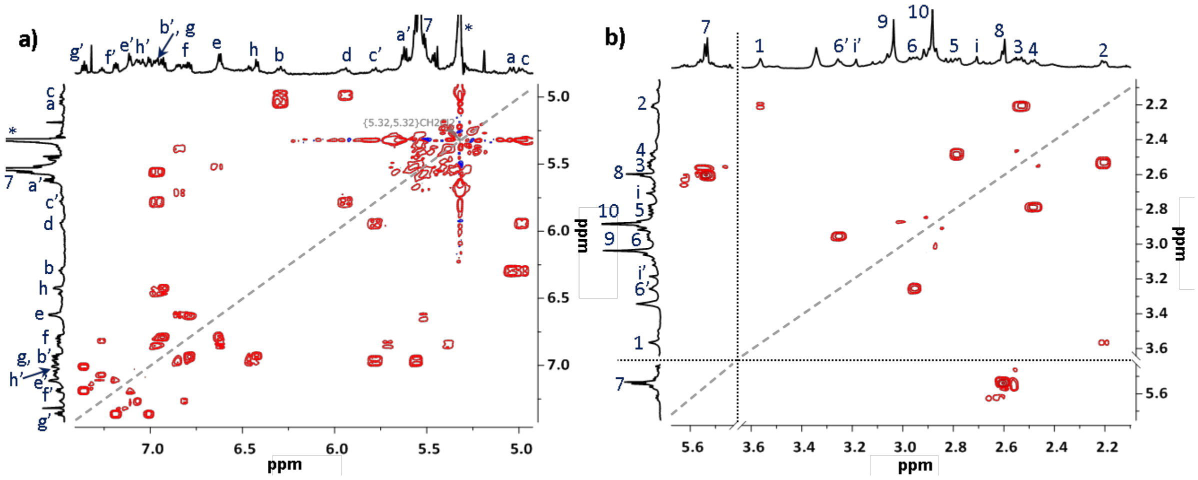

Fig. S12 Regions of the 2D $^{1}H/^{1}H$ COSY spectrum (CD_2Cl_2, 700 MHz) of the target compound **Cy7⊂*c*-P6·T6*** showing the correlations between **a**) the **Cy7** dye protons, and **b**) the protons on **T6*** (the spectra are cut between δ = 3.65 – 5.45 to allow H_7 to be observed on the same diagram). All protons are labeled according to **Fig. S11**, and the solvent residue ($CDHCl_2$) is marked with '*'. The diagonal peaks have been suppressed for simplification.

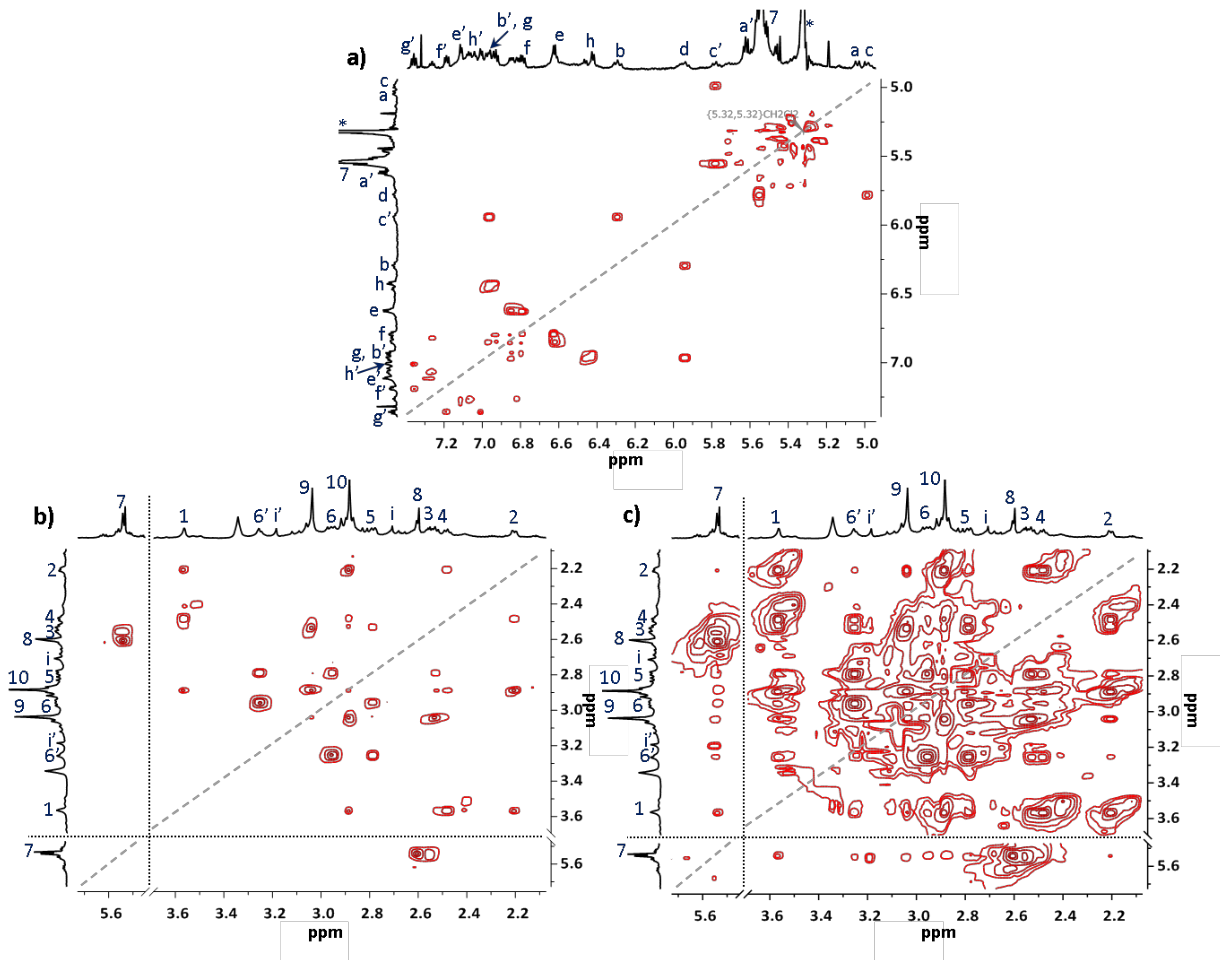

Fig. S13 Regions of the 2D $^1H/^1H$ NOESY spectrum (CD_2Cl_2, 700 MHz) of the target compound **Cy7⊂*c*-P6·T6*** showing the correlations between **a**) the **Cy7** dye protons, **b**) the protons on **T6*** (lower intensity), and **c**) the protons on **T6*** (same as **a**). For **b** and **c**, the spectra have been cut between *δ* = 3.65 – 5.45 to allow H_7 to be observed on the same diagram. All protons are labeled according to **Fig. S11**, and the solvent residue ($CDHCl_2$) is marked with '*'. The diagonal peaks have been suppressed for simplification.

NOE correlations between the dye backbone and the functionalized cyclodextrin (**T6***) are characteristic of the rotaxane structure (**Figs S12** and **S13**, **Table S4**). The correlations between $H_{j,k}$ and $H_{6,6'}$ confirm that the narrow rim of **T6*** is at one end of the backbone, and the correlations between $H_{i',j',k'}$ and $H_{9,10}$ confirm that the wider rim is facing the other end. However, unlike **Cy7⊂T6*** (**Table S3**), no correlation between $H_{7,8}$ and $H_{i,j,k}$ is observed. Surprisingly, the correlations between H_7 and $H_{i',j',k'}$ show that the pyridyl groups are closer to the other end, where the wider rim is facing. Other strong correlations between H_7 and the cyclodextrin protons, including $H_{9,10}$ on the other rim, also indicate the direction in which H_7 is pointing, and support conformation **A** rather than conformer **B**.

COSY (upper triangle) / NOESY (lower triangle)

H	1	2	3	4	5	6, 6′	7	8	9	10	a	b	c	d	a'	b'	c'	i	j, k	i'	j', k′
1	\	green	gray	gray	gray	gray	gray	gray	gray	gray	gray	gray	gray	gray	gray	gray	gray	gray	gray	gray	gray
2	green	\	green	gray	gray	gray	gray	gray	gray	gray	gray	gray	gray	gray	gray	gray	gray	gray	gray	gray	gray
3	gray	gray	\	green	gray	gray	gray	gray	gray	gray	gray	gray	gray	gray	gray	gray	gray	gray	gray	gray	gray
4	green	green	gray	\	green	gray	gray	gray	gray	gray	gray	gray	gray	gray	gray	gray	gray	gray	gray	gray	gray
5	yellow	yellow	green	yellow	\	gray	gray	gray	gray	gray	gray	gray	gray	gray	gray	gray	gray	gray	gray	gray	gray
6, 6′	yellow	yellow	gray	yellow	green	\	gray	gray	gray	gray	gray	gray	gray	gray	gray	gray	gray	gray	gray	gray	gray
7	green	green	gray	green	yellow	green	\	green	gray	gray	gray	gray	gray	gray	gray	gray	gray	gray	gray	gray	gray
8	gray	gray	gray	gray	gray	gray	green	\	gray	gray	gray	gray	gray	gray	gray	gray	gray	gray	gray	gray	gray
9	yellow	yellow	green	gray	yellow	yellow	green	gray	\	gray	gray	gray	gray	gray	gray	gray	gray	gray	gray	gray	gray
10	green	green	yellow	green	gray	gray	green	f (gray)	green	\	gray	gray	gray	gray	gray	gray	gray	gray	gray	gray	gray
a	gray	gray	gray	gray	green	green	gray	gray	gray	gray	\	green	gray	gray	gray	gray	gray	gray	gray	gray	gray
b	gray	gray	gray	yellow	green	green	gray	gray	gray	gray	gray	\	green	gray	gray	gray	gray	gray	gray	gray	gray
c	gray	gray	yellow	yellow	green	green	gray	gray	gray	gray	green	gray	\	green	gray	gray	gray	gray	gray	gray	gray
d	gray	gray	green	yellow	green	green	gray	gray	yellow	gray	gray	green	gray	\	gray	gray	green	gray	gray	gray	gray
a'	gray	gray	gray	gray	gray	gray	gray	gray	green	gray	gray	gray	gray	gray	\	green	gray	gray	gray	gray	gray
b'	gray	gray	green	gray	green	yellow	gray	gray	green	gray	gray	gray	gray	green	gray	\	green	gray	gray	gray	gray
c'	gray	gray	green	yellow	green	green	gray	gray	green	gray	gray	gray	green	gray	green	gray	\	gray	gray	gray	gray
i	gray	gray	gray	gray	gray	gray	gray	gray	gray	gray	green	gray	yellow	gray	gray	gray	gray	\	gray	gray	gray
j, k	gray	gray	gray	gray	gray	green	gray	gray	gray	gray	gray	green	gray	gray	gray	gray	gray	gray	\	gray	gray
i'	gray	gray	gray	gray	gray	gray	green	gray	yellow	gray	gray	gray	gray	gray	green	gray	yellow	gray	gray	\	gray
j', k′	gray	gray	gray	gray	gray	gray	yellow	gray	green	yellow	gray	gray	gray	gray	gray	green	gray	gray	gray	gray	\

Table S4 Summary of the COSY and NOESY correlations (CD_2Cl_2, 700 MHz) between protons of **Cy7⊂*c*-P6·T6*** (green = strong, yellow = weak, and gray = no correlation). The **T6*** protons are labeled in numbers, and the **Cy7** dye protons are labeled alphabetically according to **Fig. S11**. The aromatic protons of the dye and the ***c*-P6** protons were ignored.

The protons inside the cyclodextrin cavity ($H_{3,\ 5}$) show many NOE correlations to the **Cy7** bridge, and surprisingly an outside proton (H_4) also shows weak NOE correlations to $H_{b,c,d,c'}$. (**Fig. S15**, **Table S4**). The correlations between the protons (H_6 and H_9) on both rims of **T6***, and the **Cy7** bridge are observed, suggesting that the cyclodextrin is positioned in the middle of the dye backbone, supporting conformer **A**. However, the data from the ^{1}H-NMR spectra are not enough to support which conformer is more likely. As mentioned earlier, it is possible that the two conformers are exchangeable at room temperature, since the energy difference between the two is predicted to be small.

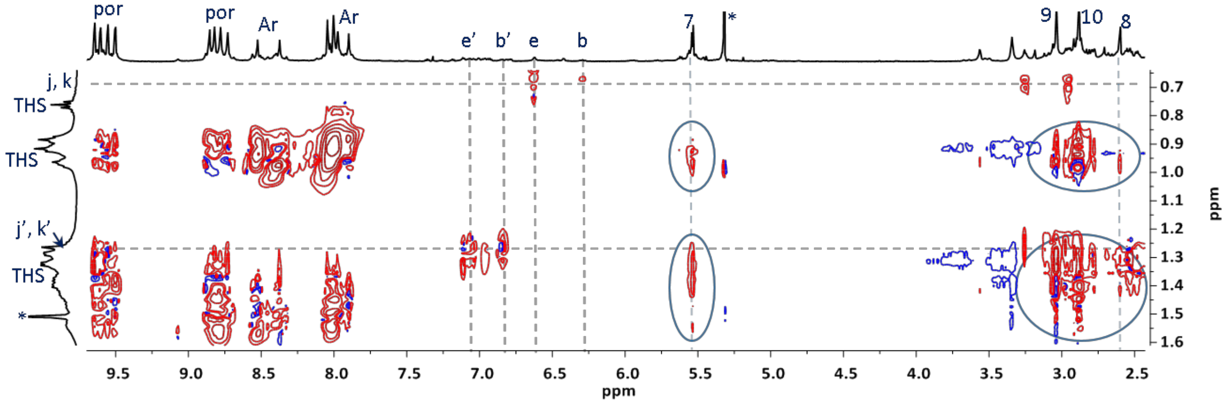

Fig. S14 Region of the 2D ^{1}H/^{1}H NOESY spectrum (CD_2Cl_2, 700 MHz) of **Cy7⊂*c*-P6·T6*** showing the correlations of the THS protons (H_{THS}) with other protons. The correlations between H_{THS} and **T6*** protons are marked in the blue regions. The positions of H_j, H_k, $H_{j'}$ and $H_{k'}$ are marked with the horizontal dashed lines, and the positions of $H_{e'}$, $H_{b'}$, H_e and H_b are marked with the vertical dashed lines. All protons are labeled according to **Fig. S11**, and the solvent residue ($CDHCl_2$) is marked with '*'.

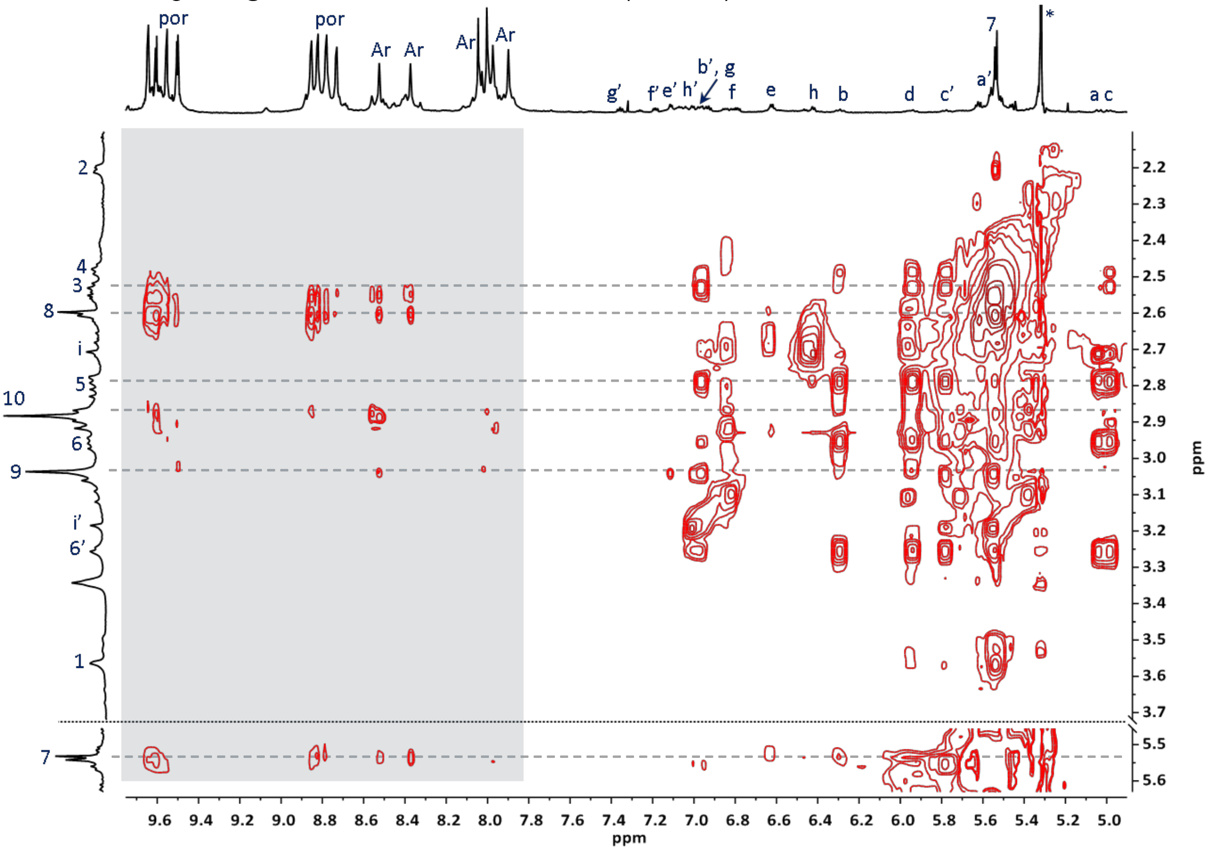

Fig. S15 Region of the 2D ^{1}H/^{1}H NOESY spectrum (CD_2Cl_2, 700 MHz) of **Cy7⊂*c*-P6·T6*** showing the correlations of the **T6*** protons (vertical axis) with other protons (horizontal axis). The correlations between $H_{THS,Ar}$ and **T6*** protons are highlighted in blue. Some **T6*** protons are marked with gray dashed lines. All protons are labeled according to **Fig. S11**, and the solvent residue ($CDHCl_2$) is marked with '*'.

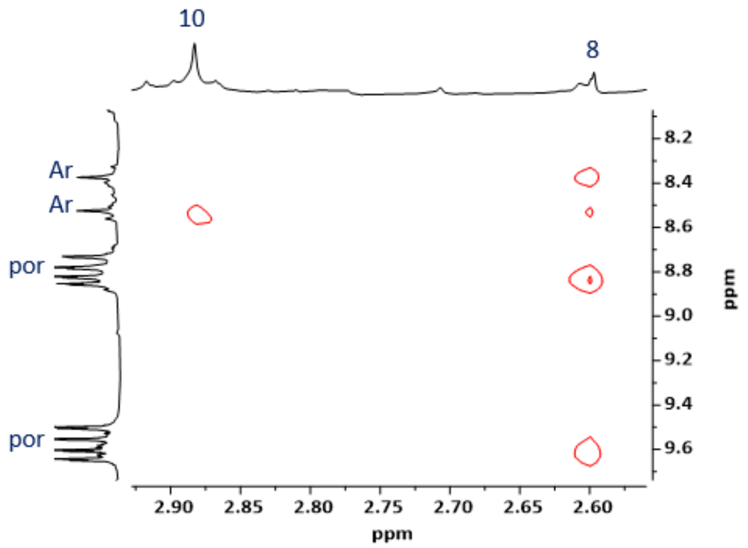

Fig. S16 Region of the 2D $^1H/^1H$ NOESY spectrum (CD_2Cl_2, 700 MHz) of **Cy7⊂*c*-P6·T6*** showing proof of template complexation. The correlations between H_{10} (cyclodextrin methoxy)/$\mathbf{H_8}$ (pyridyl α) protons and the porphyrin/solubilizing aryl protons of the nanoring highlight complexation of the supramolecular structure.

MALDI-TOF mass spectra of **Cy7⊂*c*-P6·T6*** are consistent with the proposed structure. The peak due to the molecular ion of **Cy7⊂*c*-P6·T6*** was detected at low laser power (*m*/*z* 12,391.2, **Fig. S33**). Decomplexation was observed during laser desorption, and the signal due to the empty ***c*-P6** was also detected. The relative ***c*-P6** to **Cy⊂*c*-P6·T6*** peak heights increased with the laser power, and only the ***c*-P6** signal was observed at high laser power (**Fig. S34**).

5 Spectra of New Compounds

This section presents the UV, NMR and MS spectra of the rotaxane **4** (**Figs S17–S23**), the rotaxane template **Cy7⊂T6*** (**Figs S24–S29**) and the target compound **Cy7⊂*c*-P6·T6*** (**Figs S30–S36**).

UV-vis-NIR Spectra of 4 and Cy7⊂T6*

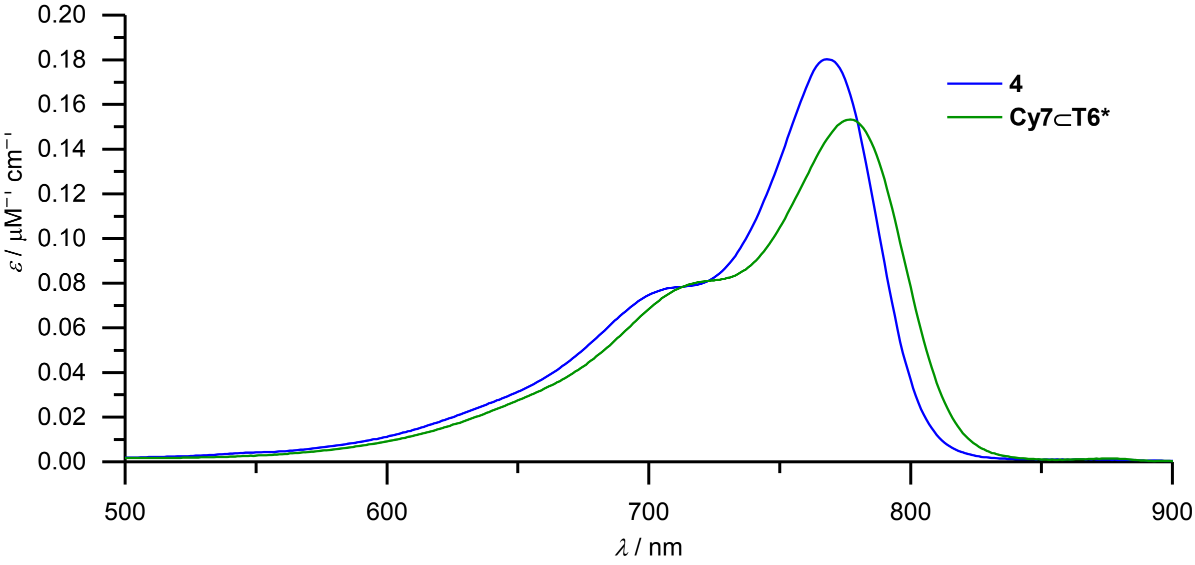

Fig. S17 UV-vis-NIR spectra of cyanines **4** (blue) and **Cy7⊂T6*** (green) (MeOH, 25 °C).

Rotaxane 4 NMR and MS

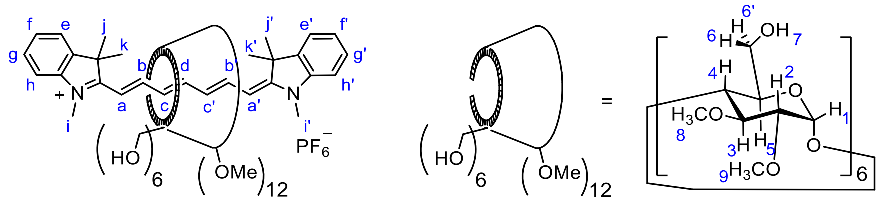

Fig. S18 Structure of the rotaxane **4**.

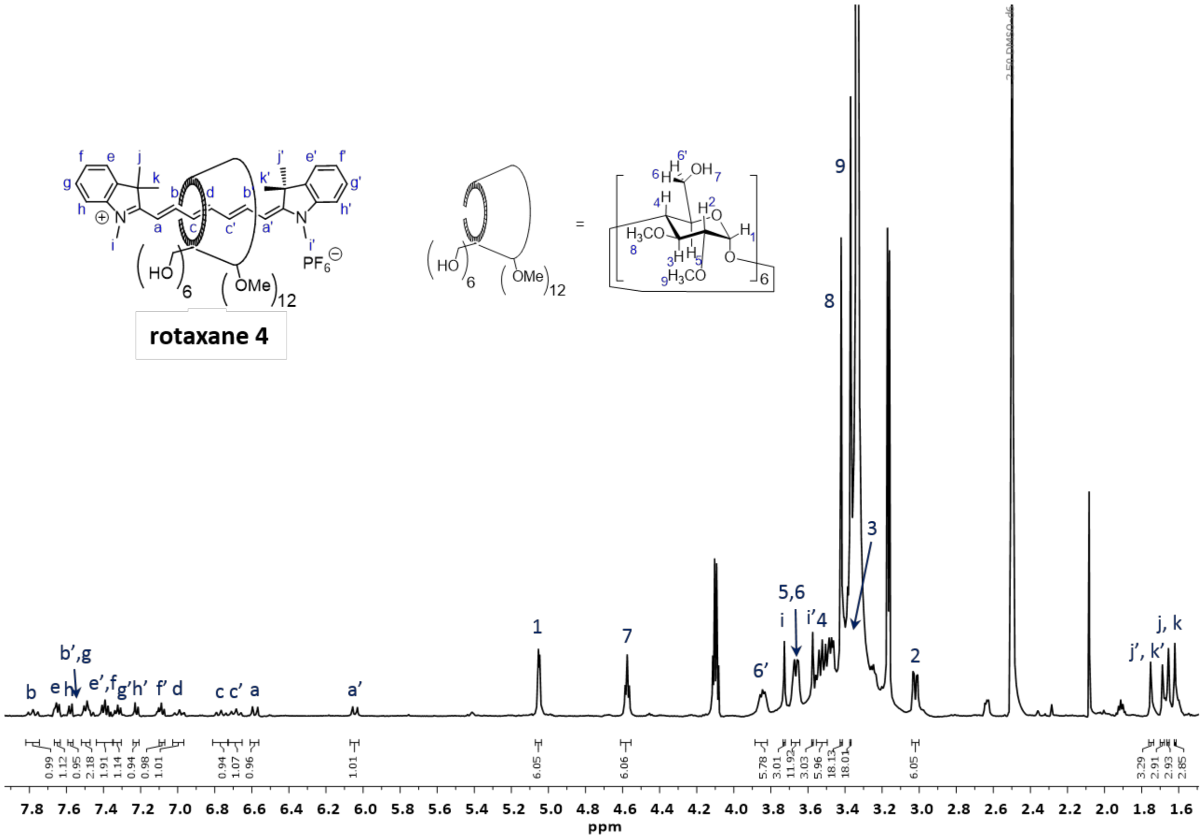

Fig. S19 ^{1}H NMR spectrum (500 MHz, *d*$_6$-DMSO) of the rotaxane **4**. Some solvent impurities are present (MeOH, water, acetone).

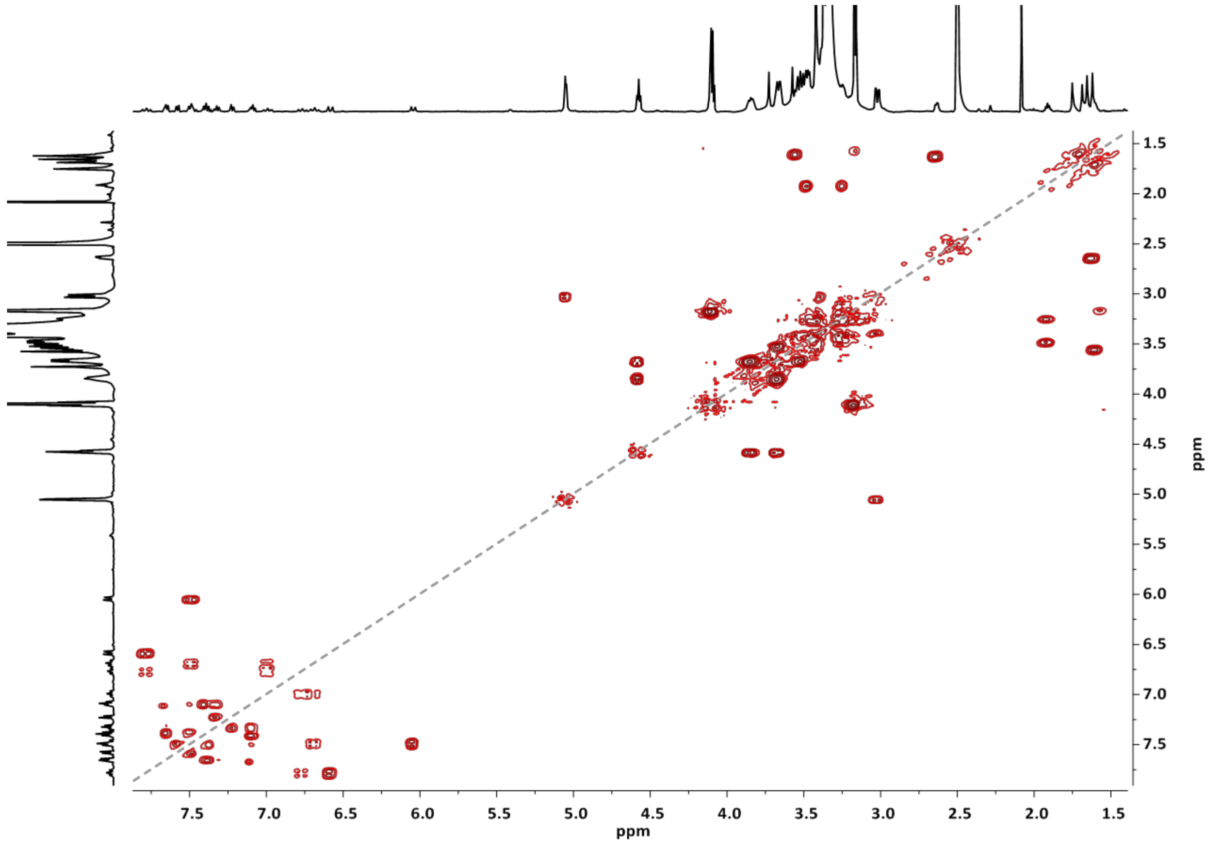

Fig. S20 2D $^{1}H/^{1}H$ COSY spectrum (500 MHz, *d*$_6$-DMSO) of the rotaxane **4**. Some solvent impurities are present (MeOH, water, acetone). The diagonal peaks have been suppressed for simplification.

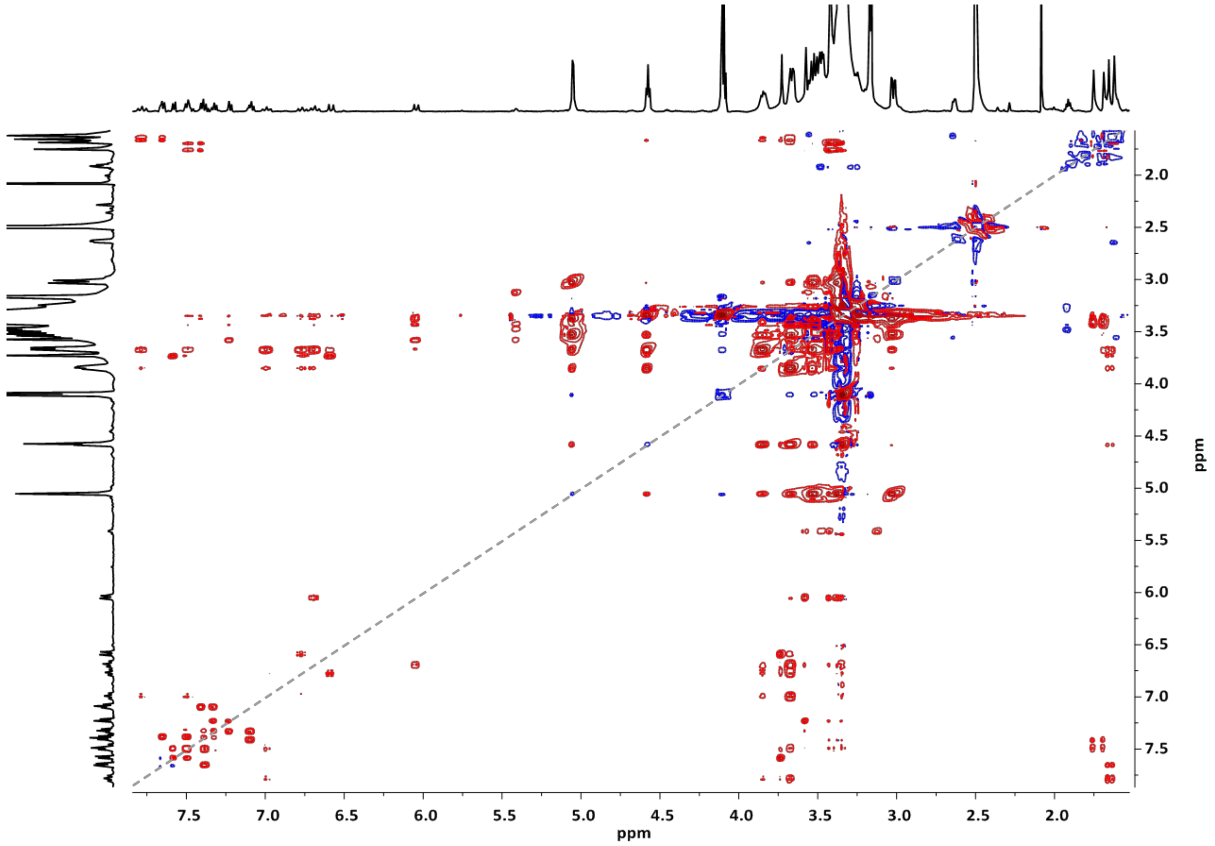

Fig. S21 2D $^1H/^1H$ NOESY spectrum (500 MHz, *d*$_6$-DMSO) of the rotaxane **4**. Some solvent impurities are present (MeOH, water, acetone). The diagonal peaks have been suppressed for simplification.

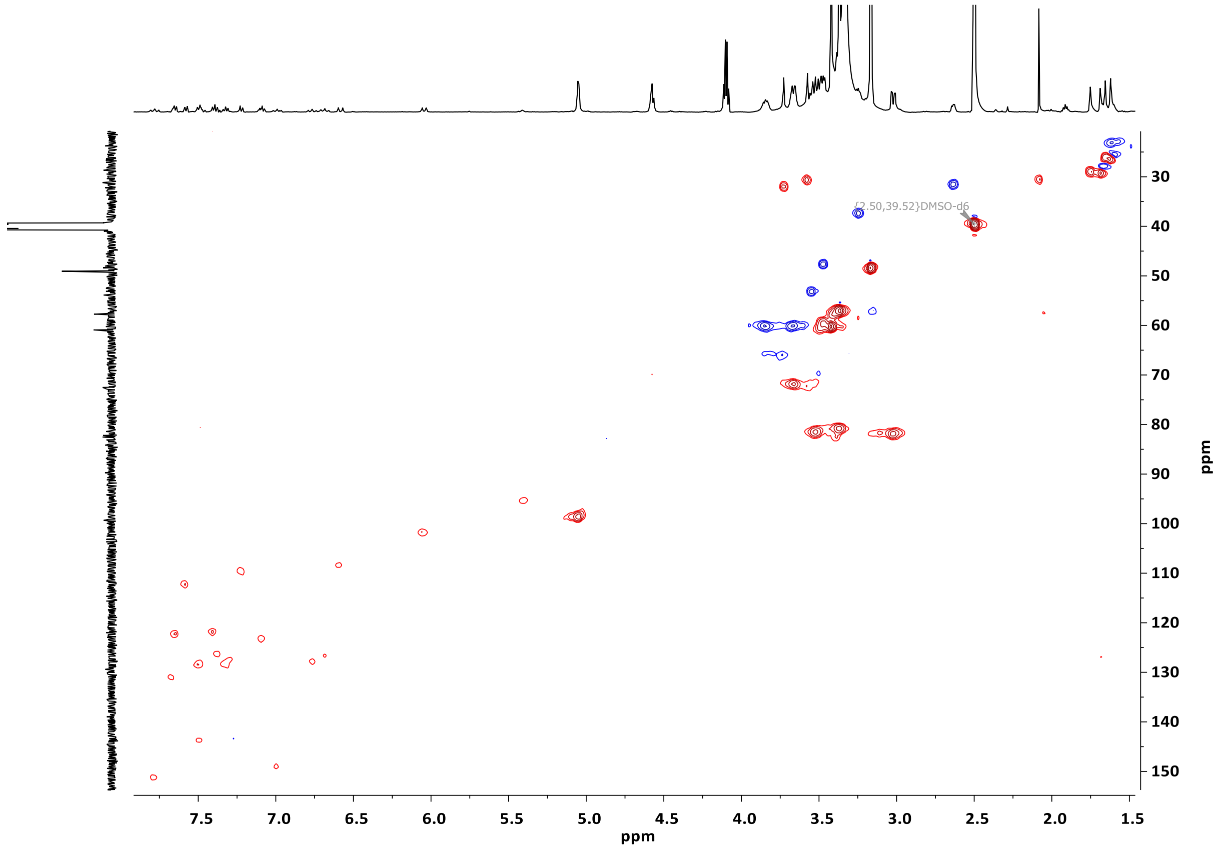

Fig. S22 2D $^1H/^{13}C$ HSQC spectrum (500 MHz, *d*$_6$-DMSO) of the rotaxane **4**. Some solvent impurities are present (MeOH, water, acetone).

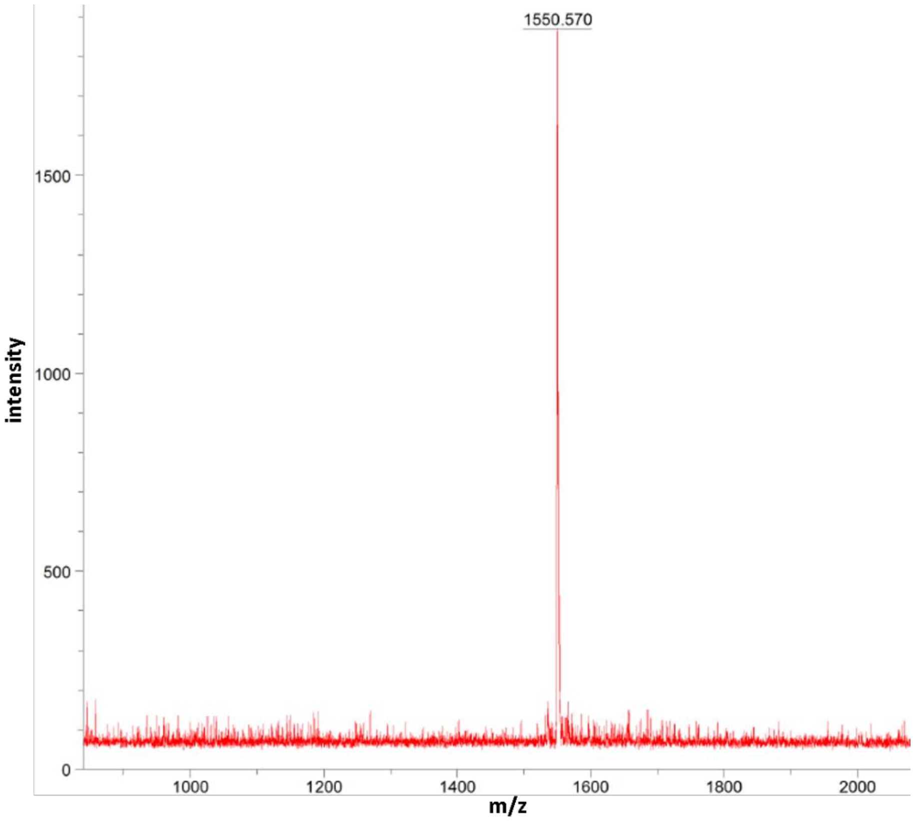

Fig. S23 MALDI-TOF mass spectrum of the rotaxane **4**. MS: *m*/*z* 1550.6 ([M]$^+$, $C_{77}H_{117}N_2O_{30}$ requires 1550.7).

Rotaxane Template Cy7⊂T6* NMR and MS

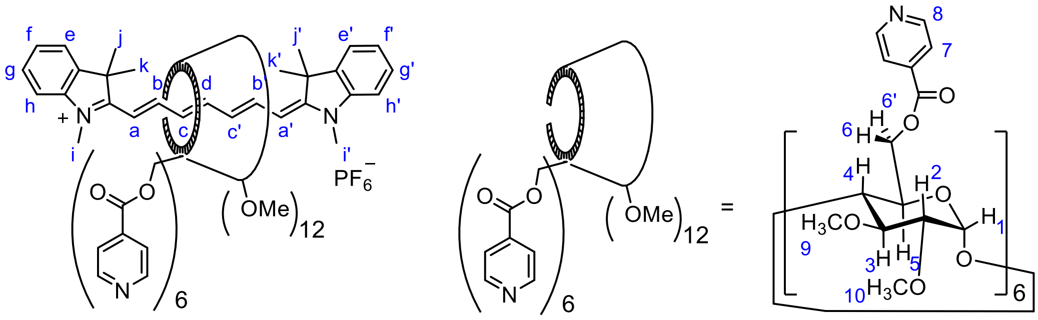

Fig. S24 Structure of **Cy7⊂T6***.

Note: The 1D ^{1}H-NMR spectrum shown for reference in the 2D spectra plotted in **Figs S26** and **S27** is that in **Fig. S25**, which contains pyridine impurity. The pyridine was removed before acquiring these 2D spectra (**Figs S26** and **S27**), but in this case the proton spectrum was broad, probably due to water impurity. The sharper 1D spectrum (**Fig. S25**) was used for clarity.

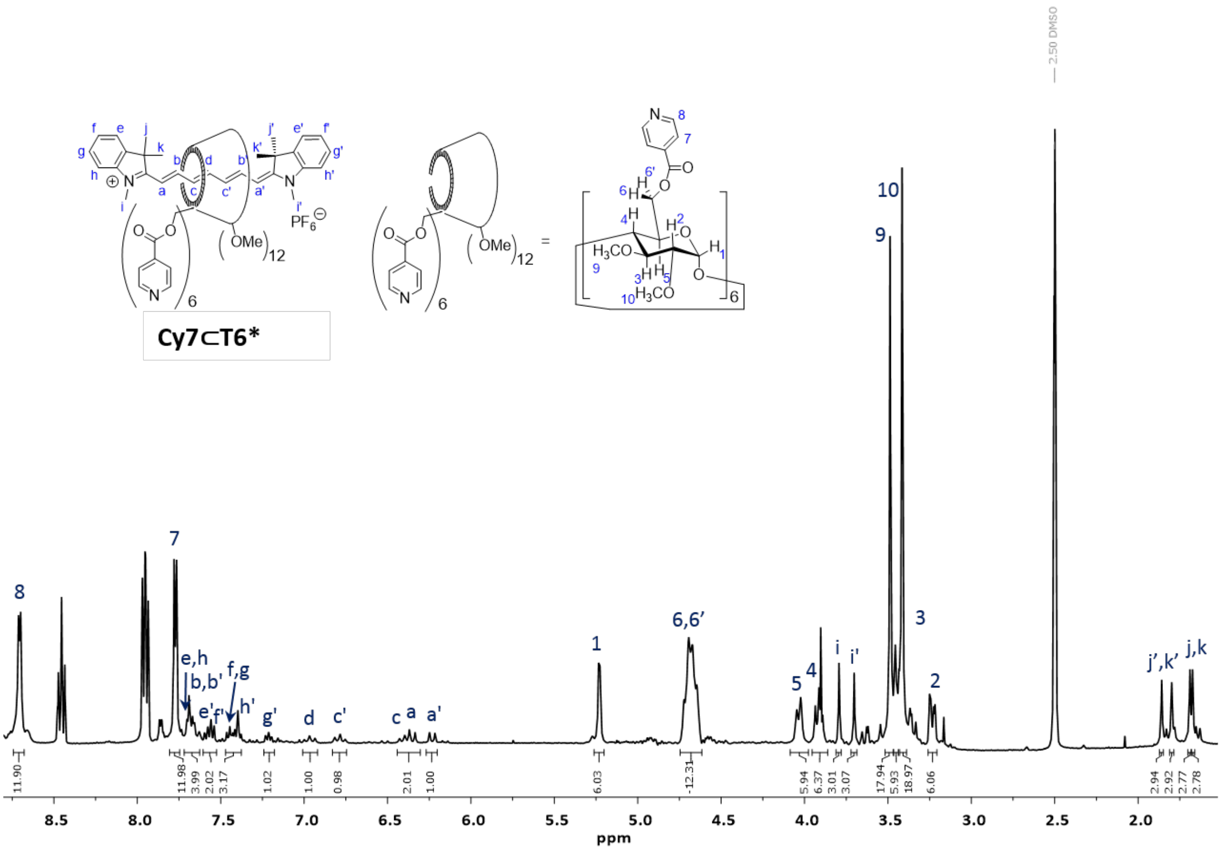

Fig. S25 ^{1}H NMR spectrum (400 MHz, d_6-DMSO) of **Cy7⊂T6***. Some solvent impurities are present (pyridine).

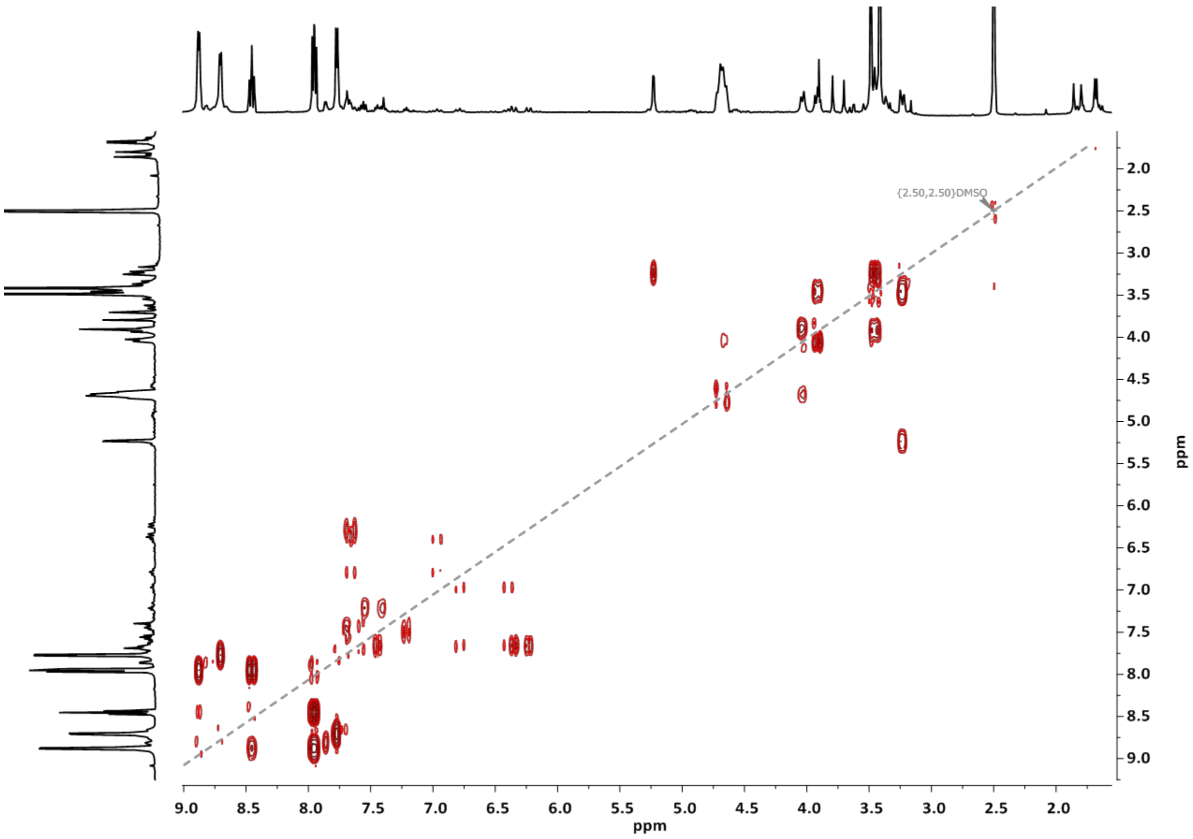

Fig. S26 2D $^{1}H/^{1}H$ COSY spectrum (400 MHz, *d*$_6$-DMSO) of **Cy7⊂T6***. Some solvent impurities are present (pyridine). The diagonal peaks have been suppressed for simplification.

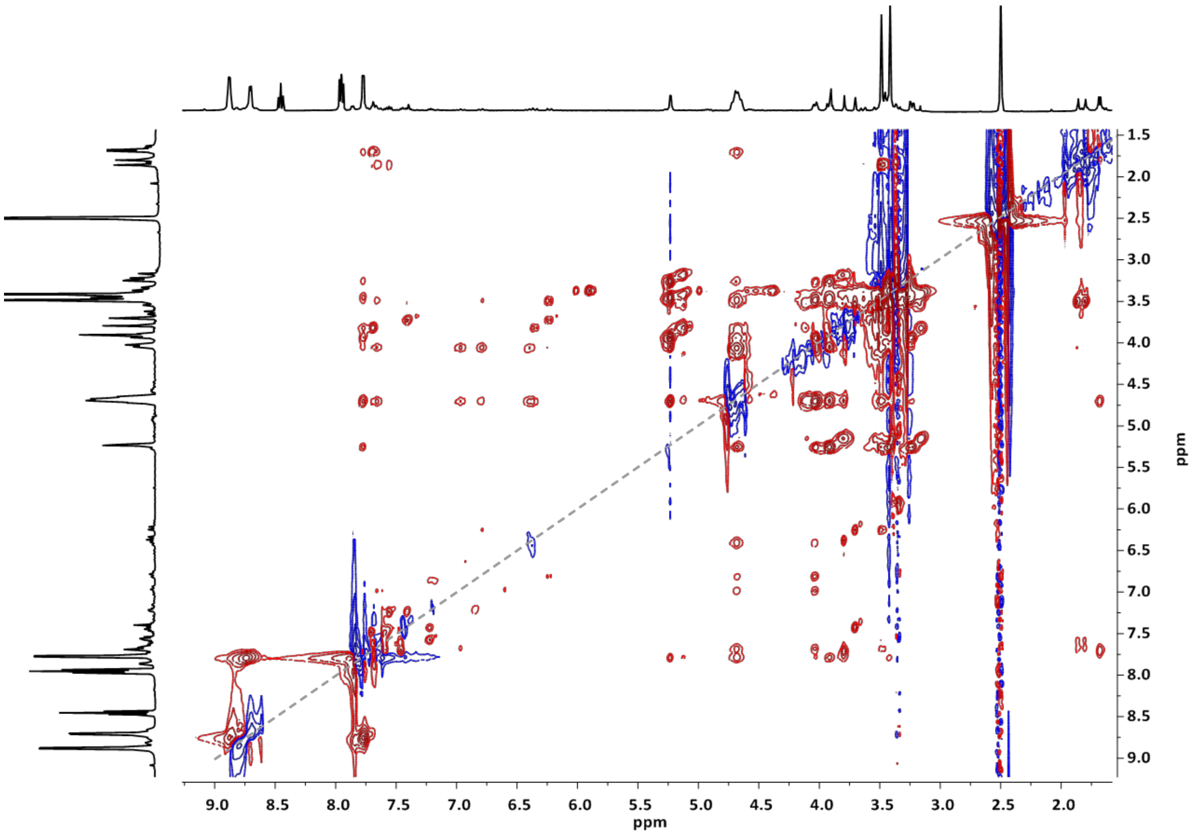

Fig. S27 2D $^{1}H/^{1}H$ NOESY spectrum (400 MHz, *d*$_6$-DMSO) of **Cy7⊂T6***. 1D spectra contain pyridine impurity peaks. The diagonal peaks have been suppressed for simplification.

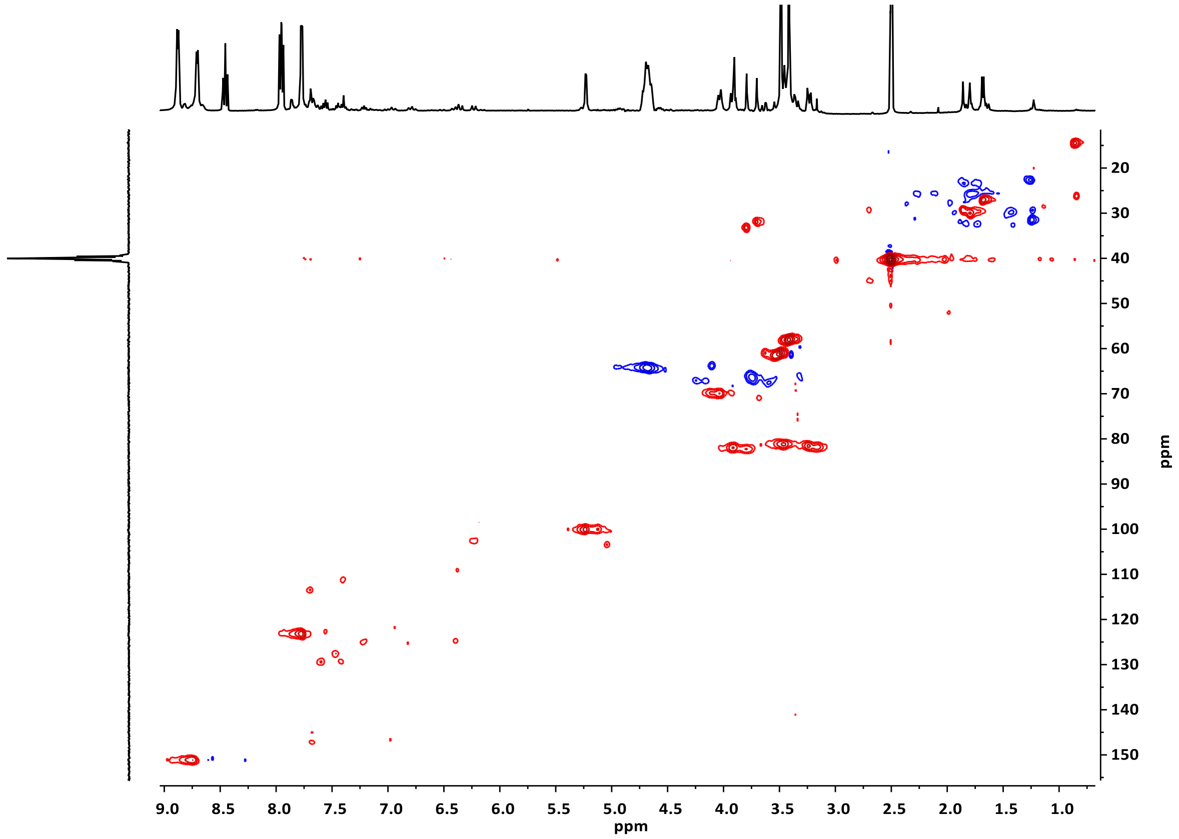

Fig. S28 2D $^{1}H/^{13}C$ HSQC spectrum (400 MHz, *d*$_6$-DMSO) of **Cy7⊂T6***. 1D ^{1}H-NMR contains pyridine impurity peaks.

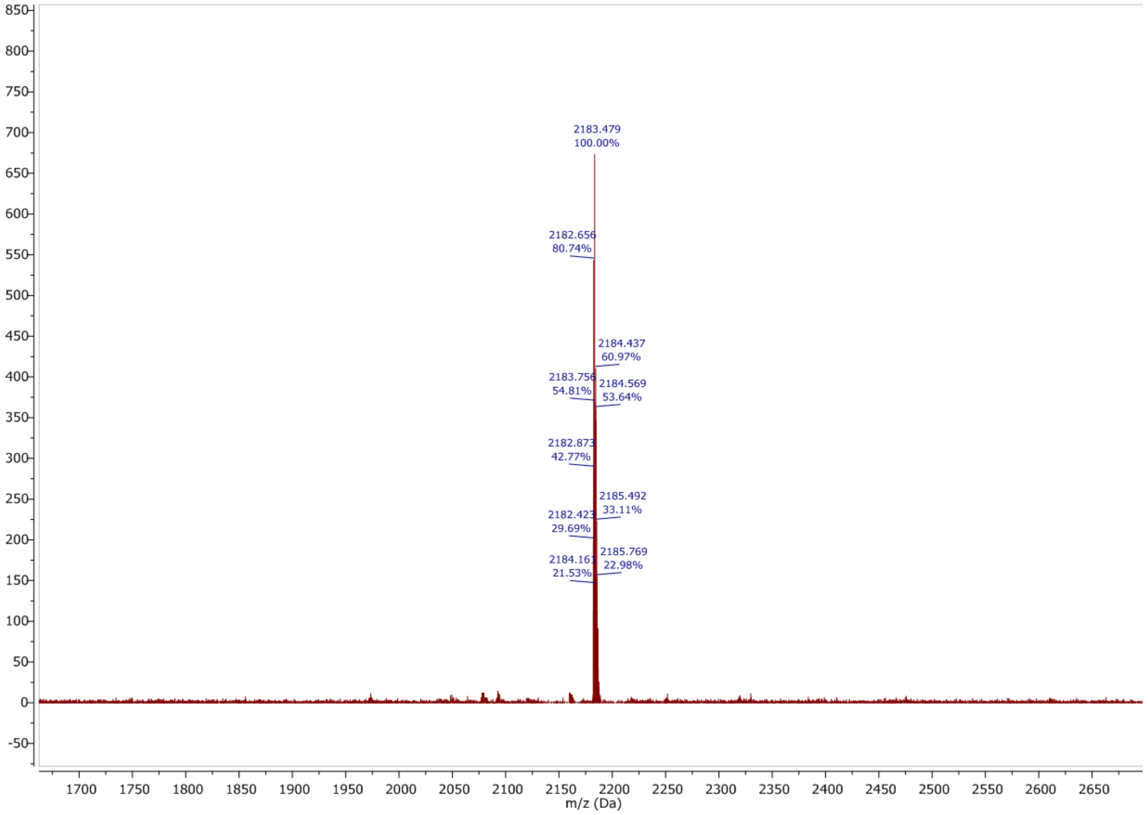

Fig. S29 ESI mass spectrum of **Cy7⊂T6***. *m*/*z* 2183.5 ($[M]^+$, $C_{113}H_{135}N_8O_{36}$ requires 2181.3).

Target compound Cy7⊂*c*-P6·T6*

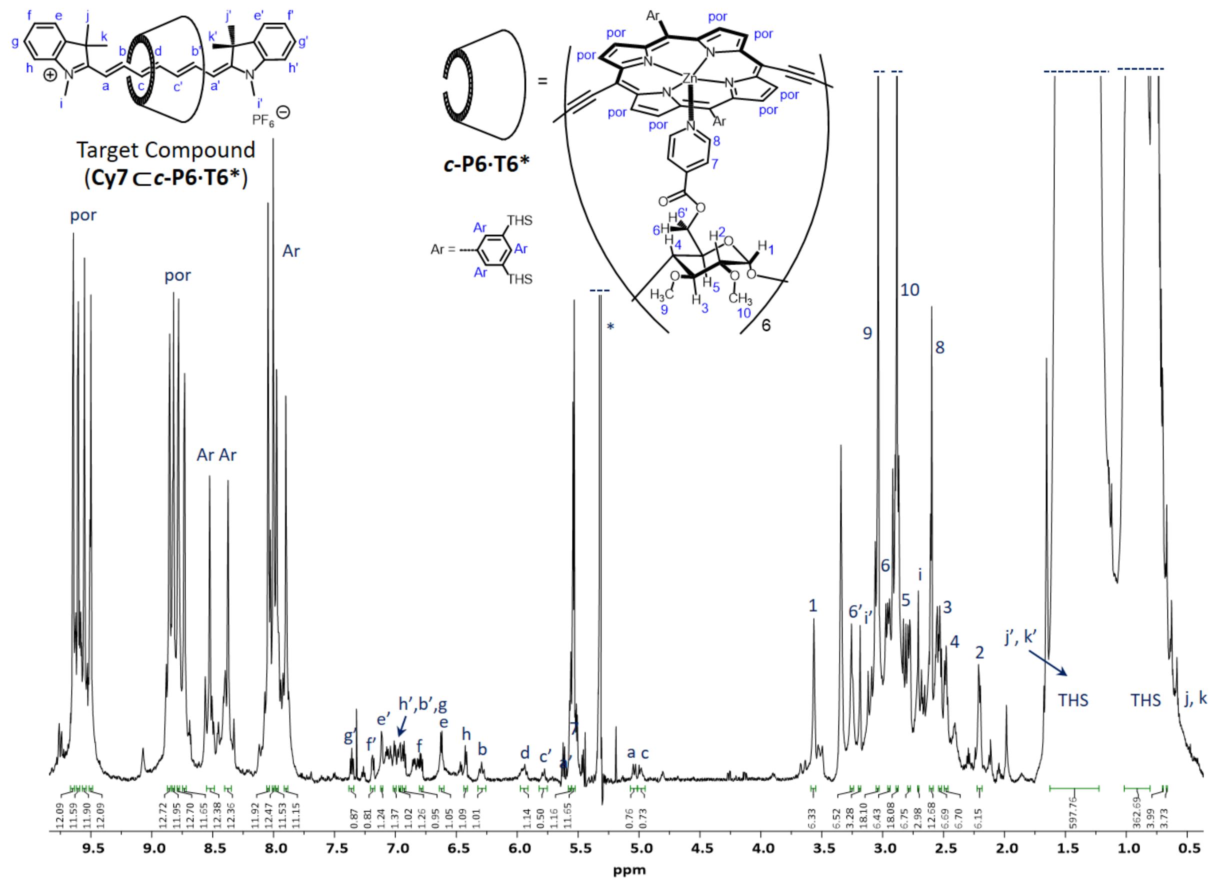

Fig. S30 ^{1}H-NMR spectrum (CD_2Cl_2, 700 MHz) of **Cy7⊂*c*-P6·T6***. The pyridyl proton signals are labeled in blue, and the solvent peaks ($CDHCl_2$ and water) is labeled with '*'.

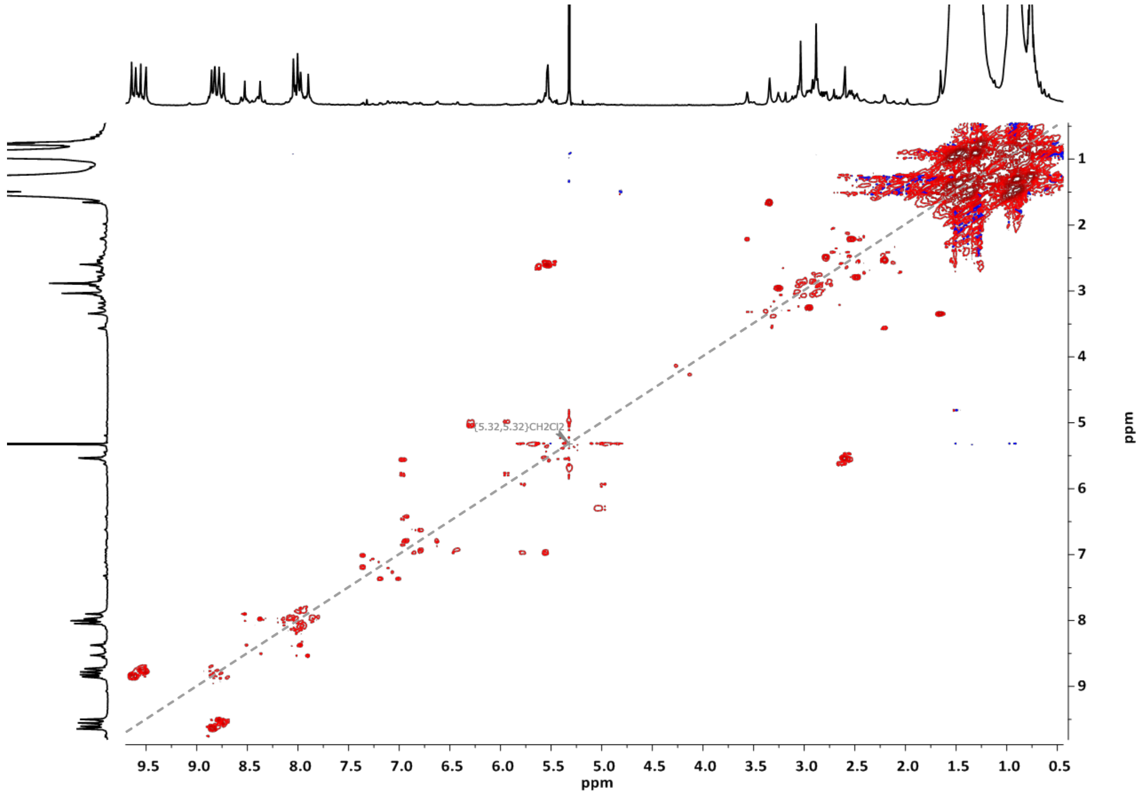

Fig. S31 2D ^{1}H/^{1}H COSY spectrum (700 MHz, CD_2Cl_2) of **Cy7⊂*c*-P6·T6***. Some solvent impurities are present ($CDHCl_2$, water). The diagonal peaks have been suppressed for simplification.

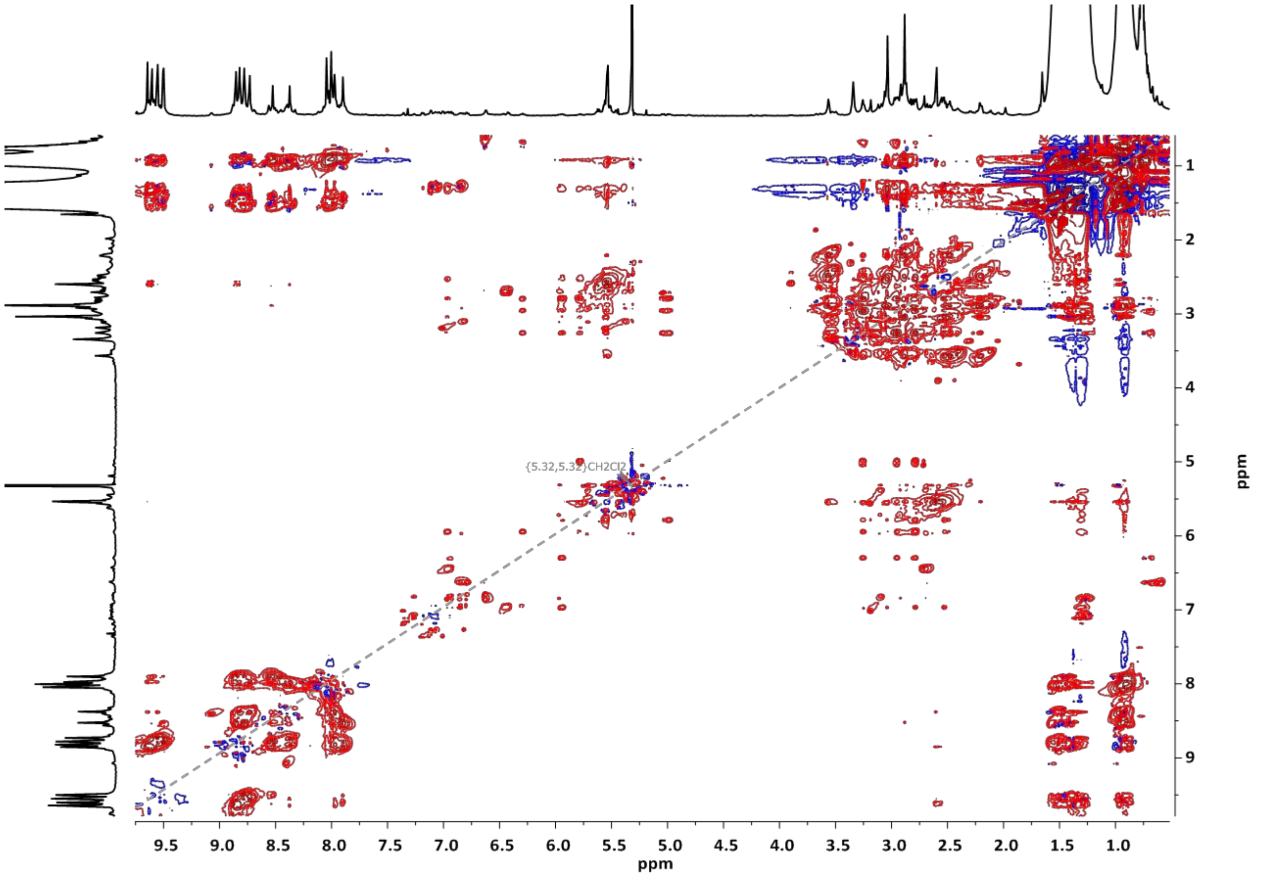

Fig. S32 2D $^{1}H/^{1}H$ NOESY spectrum (700 MHz, CD_2Cl_2) of **Cy7⊂*c*-P6·T6***. Some solvent impurities are present ($CDHCl_2$, water). The diagonal peaks have been suppressed for simplification.

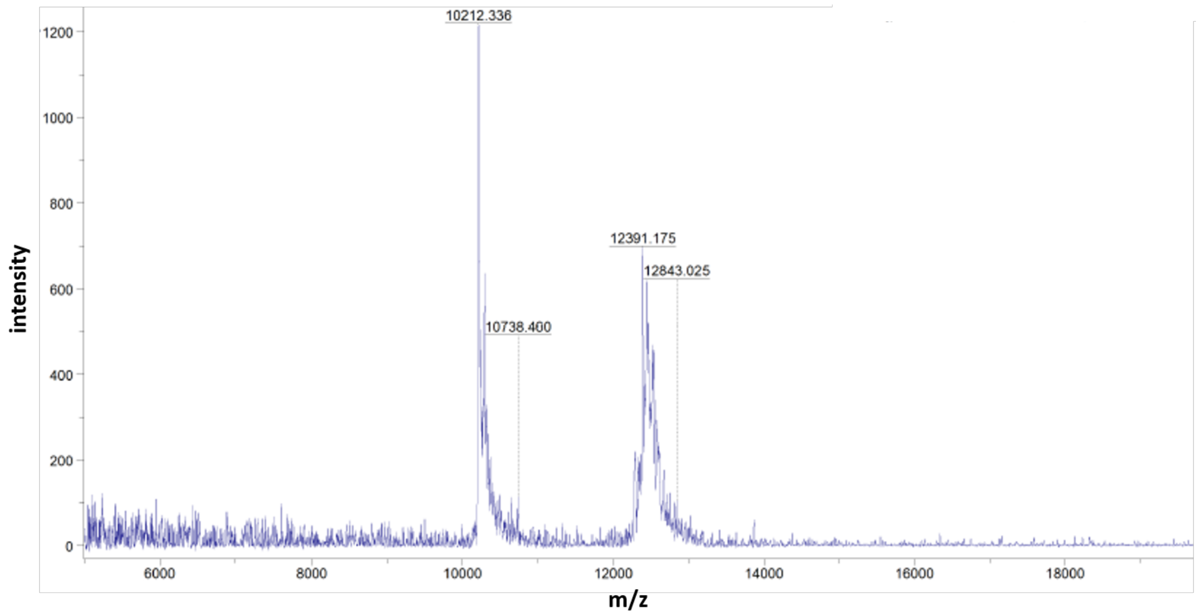

Fig. S33 MALDI-TOF mass spectrum of **Cy7⊂*c*-P6·T6*** at low laser power, *m*/*z* 12,391.2 ($[M]^+$, $C_{761}H_{1155}N_{32}O_{36}Si_{24}Zn_6$ requires 12,394.9).

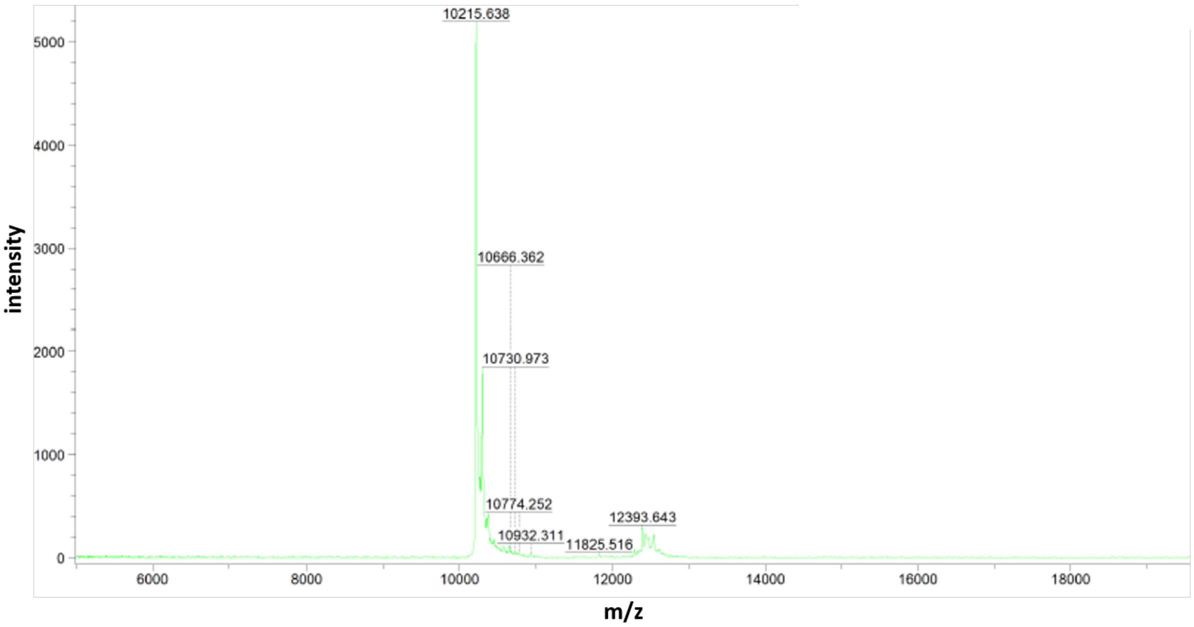

Fig. S34 MALDI-TOF mass spectrum of **Cy7⊂*c*-P6·T6*** at high laser power showing the decomplexation to generate ***c*-P6** as the major observed peak ([M-**T6***]$^+$, $C_{648}H_{1020}N_{24}Si_{24}Zn_6$ requires 10213.7).

6 Photophysical Properties

All absorption and fluorescence spectra were recorded in CH_2Cl_2 at 25 °C. Coordinating solvents such as THF would not be suitable because they reduce the stability of zinc porphyrin template complexes.

Fluorescence quantum yields

The fluorescence quantum yields (ϕ) listed in **Table S5** were measured using linear butadiyne-linked porphyrin hexamer (***l*-P6**) as a reference.[2]

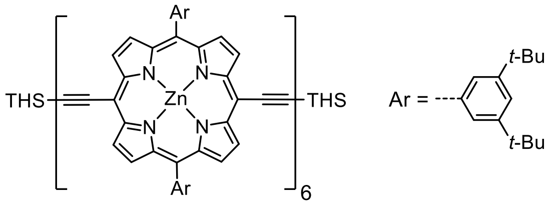

***l*-P6**

The reported quantum yield (ϕ_r) of ***l*-P6** of 28% (toluene, 1% pyridine) was further verified using an integrating sphere. The following formula was used for the calculation of the relative sample quantum yields (ϕ_s):

$$\phi_s = \phi_r \cdot \frac{1 - 10^{-A_r}}{1 - 10^{-A_s}} \cdot \frac{n_s^2}{n_r^2} \cdot \frac{\int I_s(\nu)d\nu}{\int I_r(\nu)d\nu}$$

where A_i is the optical density at the excitation wavelength, n_i the refractive index of the solvent, and $I_i(\nu)d\nu$ the integrated spectral fluorescence photon flux which was approximated by the integrated blank and dark-count corrected signals of the emission (in wave-numbers, ν).

The emission spectra were collected on the visible and NIR detectors. The spectra were stitched before integration of the peaks and calculation of the quantum yields. The low quantum yield and red-shifted emission of most of the reported compounds prevented accurate measurement of the absolute quantum yields using an integrating sphere. Several accumulated

spectra were necessary to obtain emission profiles with acceptable signal to noise. All fluorescence samples were prepared with optical densities under 0.1 under ambient conditions.

Absorption, Fluorescence and Excitation Spectra

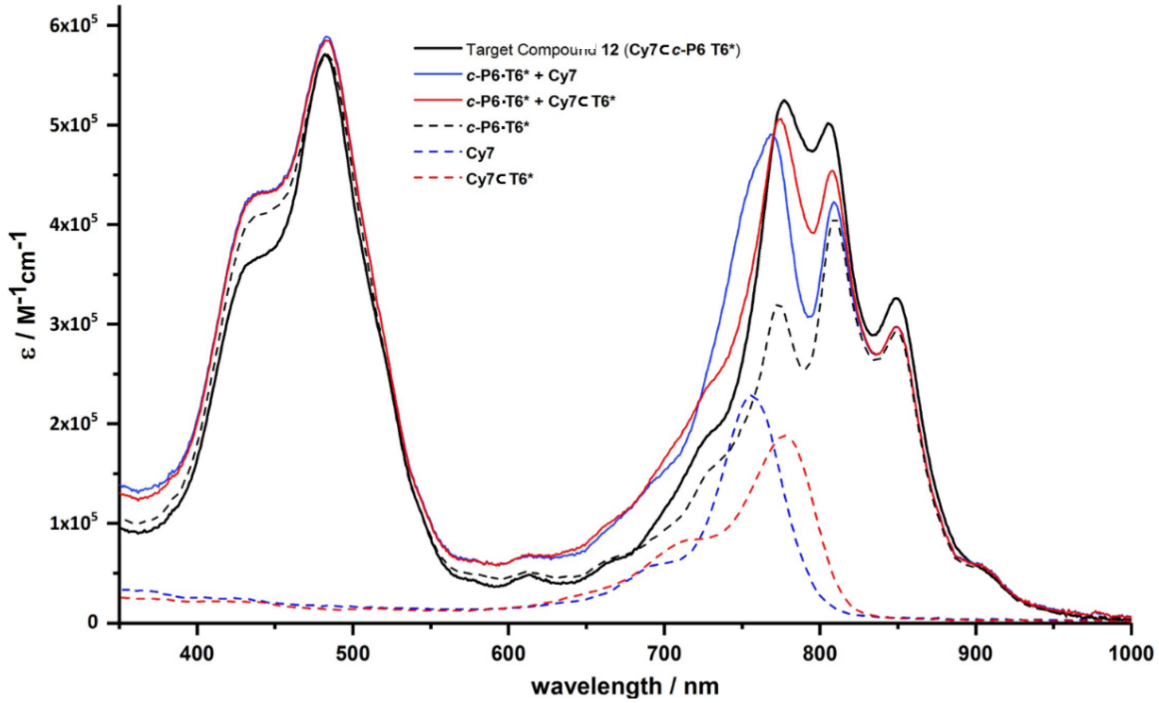

Fig. S35 Absorption spectra of the target compound **Cy7⊂*c*-P6·T6*** (black), ***c*-P6·T6*** (black dashed line), **Cy7** free dye (blue dashed line) and **Cy7⊂T6*** (red dashed line), and the addition of absorption spectra of ***c*P6·T6* + Cy7** (blue) and ***c*-P6·T6*** + **Cy7⊂T6*** (red), in CH_2Cl_2 at 25 °C.

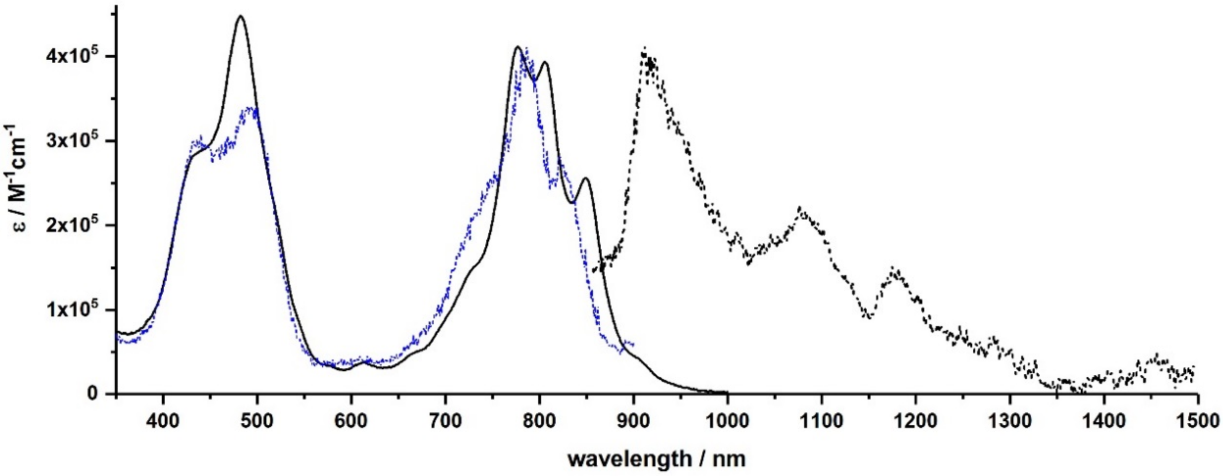

Fig. S36 Absorption (black), normalized emission (black dashed line) and normalized excitation (blue dashed line) spectra of the target compound **Cy7⊂*c*-P6·T6***, measured in CH_2Cl_2 at 25 °C. The excitation wavelength for the emission spectrum is 847 nm, and the detection wavelength for excitation spectrum is 910 nm. The apparent dip in the emission spectrum at 1150 nm is due to absorption by the solvent.

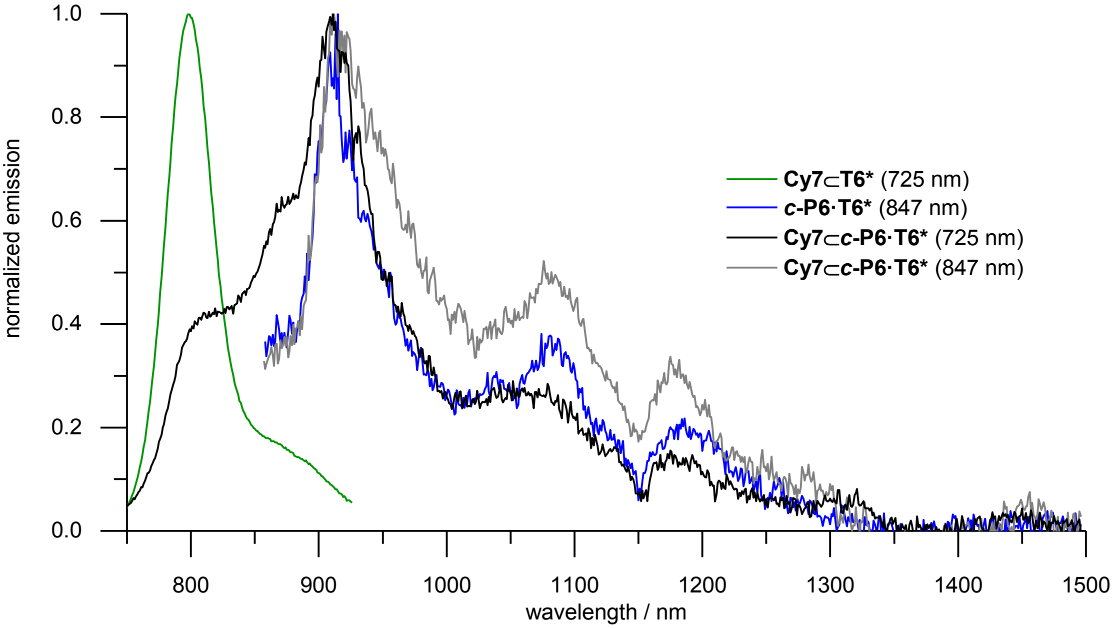

Fig. S37 Normalized emission spectra of ***c*-P6·T6*** (blue), **Cy7⊂T6*** (green) and the target compound **Cy7⊂*c*-P6·T6*** at the excitation wavelength of 725 nm (black) and 847 nm (gray), measured in CH_2Cl_2 at 25 °C. The apparent dip in the spectra at 1150 nm is due to absorption by the solvent.

compound	absorption maxima (nm) (absorption coefficient, M^{-1} cm^{-1})	quantum yield ϕ (%)
target complex **Cy7⊂*c*-P6·T6***	482 (5.71 × 10^5), 777 (5.25 × 10^5), 805 (5.01 × 10^5), 849 (3.26 × 10^5)	0.6
c*-P6·T6	483 (5.71 × 10^5), 773 (3.2 × 10^5), 809 (4.07 × 10^5), 850 (2.92 × 10^5)	0.7
empty ring ***c*-P6**	463 (5.54 × 10^5), 750 (2.70× 10^5)	5.0
rotaxane template **Cy7⊂T6***	777 (1.88 × 10^5)	36
free dye **Cy7**	755 (2.29 × 10^5)	23

Table S5 Absorption maxima, molar absorption coefficients and fluorescence quantum yields of the target compound **Cy7⊂*c*-P6·T6*** and its components, measured in CH_2Cl_2 at 25 °C. The quantum yields are calculated with respect to a linear porphyrin hexamer (***l*-P6**, quantum yield 28.0%, see **Section 6**).[2]

Fluorescence lifetime of rotaxane template Cy7⊂T6*

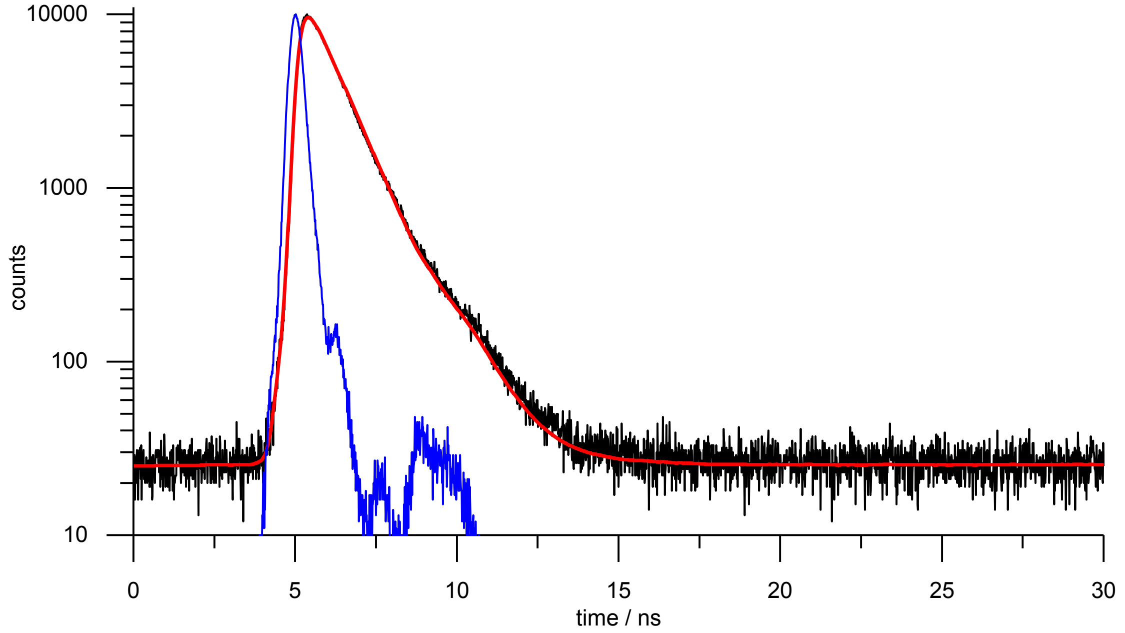

Fig. S38 Fluorescence decay (black), the instrument response function (blue) and mono-exponential reconvolution fit (red) for the fluorescence lifetime of **Cy7⊂T6*** giving a fluorescence lifetime of 0.967 ± 0.001 ns (CH_2Cl_2, 25 °C, λ_{exc} 473.4 nm, λ_{em} 800 nm). This fluorescence lifetime is similar to those reported previously for HITC dye.[8]

7 Theoretical model

Background

The goal of this section is to propose a theoretical model that can explain the changes in the absorption spectrum and the photophysics of the composite nanoring-dye system **Cy7⊂*c*-P6·T6*** as a result of the proximity of its individual components, i.e. the porphyrin nanoring and the dye, as a synthetic model of the structurally similar LH1-RC core complex of purple bacteria.[9] The first step towards a microscopic description of the photophysics of the LH1-like nanoring (***c*-P6·T6***) and the central RC-like dye (**Cy7⊂T6***) is to develop a model of the nanoring's absorption spectrum which is able to reproduce its main features. Namely, we would like to reproduce the three-peak structure of the Q-band observed in numerous experiments, and assign each peak to the corresponding excited state.

We find that in the literature there is no consensus on the microscopic origin of these peaks. Simple excitonic models are used to predict qualitatively the strong suppression of the lowest transition due to circular symmetry,[6] but their scope seems to be limited when trying to predict the energy and oscillator strength of higher lying transitions. *Ab initio* electronic structure methods such as TD-DFT or ZINDO are able to account for the energies of Q- and B-bands, but, given the large number of atoms present in the nanoring, they are computationally expensive for absorption spectrum calculations. Moreover, they are typically used on optimized geometries, which makes capturing the conformational freedom of these systems a difficult task. For this reason, TD-DFT calculations of transition energies and oscillator strengths of the Q-band yield results that are qualitatively similar to the estimates coming from simple excitonic models, predicting a forbidden low-energy transition S_1 and two degenerate bright excited states S_2 and S_3, thus failing to capture the complex three-peak structure.[6,7]

Various hypotheses regarding the nature and the origin of the three peaks have been formulated. One hypothesis was that the three peaks may be interpreted as the Franck-Condon progression of a vibrational mode of frequency 605 cm^{-1}.[7] However, no such mode was observed in porphyrin monomers and, more importantly, the highly complex emission spectra of the nanoring are not compatible with a Franck-Condon progression associated to a single electronic transition.[6] Alternatively, it was hypothesized that the three peaks could come from vibronic mixing of the bright S_2 and S_3 transitions with some known vibrational modes of the porphyrin,[7] e.g. the metal-pyrrole breathing mode at 380 cm^{-1} or a higher frequency stretching mode at 1340 cm^{-1}.[10,11] Recent two-dimensional electronic spectroscopy (2DES) experiments[12] partially confirmed this hypothesis, demonstrating vibronic mixing between the middle absorption peak of the Q-band (S_3) and the 380 cm^{-1} mode. However, the same experiment also showed that this vibronic feature plays only a minor role in determining the structure of the Q-band absorption, showing up as a shoulder around 12000 cm^{-1}. Additionally, analysis of the rephasing 2DES oscillation maps showed that the three strongest peaks in the absorption spectrum of the nanoring all originate from purely electronic transitions. Despite its success in explaining the 2DES data, the theoretical model does not clarify the microscopic origin of the observed electronic transitions, which thus remains uncertain.

Geometry and Hamiltonian of the nanoring

Our goal is to explain the origin of the three peaks in the Q-band absorption in terms of an excitonic model. Excitonic models are typically used to describe excited states of chromophore

aggregates, where resonant energy transfer occurs (possibly both coherently and incoherently) without charge carriers being transferred from one chromophore to the other.[13] Small butadiyne-linked porphyrin nanorings do not fall into this category, since they present full π-conjugation across the whole aggregate. Nonetheless, excitonic models were already successfully employed in the description of optical transitions of conjugated systems.[14] Moreover, as mentioned above, they are able to predict the same selection rules that are also deduced from *ab initio* calculations for only a small fraction of the computational cost, offering at the same time more flexibility to explore the effects of conformational disorder and vibronic effects. Even in the absence of input parameters from quantum chemical calculations (e.g. couplings, transition dipole moments and optical transition energies of monomers), they can be benchmarked against experimentally obtained spectroscopic data.

Each of the Q-band of porphyrins consists of two transitions, typically referred to as Q_x and Q_y, with perpendicular dipole moments $\mathbf{d}_x$ and $\mathbf{d}_y$. Their respective transition energies ε_x and ε_y generally depend on the groups attached to the *meso* positions of the porphyrin macrocycle. If we denote the corresponding excited states as $|\mathrm{Q}_x^n\rangle$ and $|\mathrm{Q}_y^n\rangle$ (where $n = 1, \ldots, N$ labels different porphyrins in the nanoring), we can write the following tight-binding Hamiltonian

$$\begin{aligned} H_{\text{ring}} = & \sum_{n=1}^{N} \varepsilon_x |\mathrm{Q}_x^n\rangle\langle \mathrm{Q}_x^n| + \sum_{n=1}^{N} v_{xx} \big(|\mathrm{Q}_x^n\rangle\langle \mathrm{Q}_x^{n+1}| + |\mathrm{Q}_x^{n+1}\rangle\langle \mathrm{Q}_x^n| \big) \\ & + \sum_{n=1}^{N} \varepsilon_y |\mathrm{Q}_y^n\rangle\langle \mathrm{Q}_y^n| + \sum_{n=1}^{N} v_{xx} \big(|\mathrm{Q}_y^n\rangle\langle \mathrm{Q}_y^{n+1}| + |\mathrm{Q}_y^{n+1}\rangle\langle \mathrm{Q}_y^n| \big) \\ & + \sum_{n=1}^{N} v_{xy} \big(|\mathrm{Q}_y^n\rangle\langle \mathrm{Q}_x^{n+1}| + |\mathrm{Q}_x^n\rangle\langle \mathrm{Q}_y^{n+1}| + \text{H.c.} \big), \end{aligned} \tag{S1}$$

which includes nearest-neighbor interactions between the Q_x bands (v_{xx}), Q_y bands (v_{yy}) and Q_x-Q_y mixing (v_{xy}). In the following, we focus on the case $N = 6$ to address the six porphyrins in ***c*-P6·T6***. The model can be refined by allowing for non-uniform couplings and site energies, e.g. if the nanoring exhibits conformational disorder or some degree of dimerization. Indeed, the latter seems to be present in templated nanorings: As it can be observed from the crystal structure of ***c*-P6·T6**, porphyrin macrocycles exhibit a slight out-of-plane alternating tilt of their *meso* carbons at positions 5 and 15 of about $\phi \approx 10^\circ$.[7] Thus, in our model we allow the transition dipoles $\mathbf{d}_x$ along the nanoring to have alternating angles $\pm\phi$ out of the nanoring plane. Consequently, the dipoles $\mathbf{d}_y$ exhibit alternating out-of-plane angle $90^\circ \pm \phi$. This geometry is schematically represented in Fig. S39. Another feature evident from the structure of ***c*-P6·T6***, is a tilt of the Q_y transition dipole moments away from the direction perpendicular to the nanoring's plane towards the center of the complex, shown as θ in Fig. S39.

Disorder and dephasing

As already mentioned, excitonic models give us more flexibility when exploring how different sources of disorder influence optical properties. We can include inhomogeneous broadening of the spectrum adding to the Hamiltonian H_{ring} of Eq. (S1) a stochastic part δH_{ring} that averages to zero. Absorption spectra are then computed for each realization of δH_{ring}, thus taking the average over the ensemble of realizations yields the inhomogeneously broadened spectrum. Since all the poprhyrins that make up a ring are immersed in the same environment, we expect that they will experience the same fluctuations. Therefore, we consider a global shift of the energies ε_x and ε_y, which we assume to be normally distributed around zero with standard

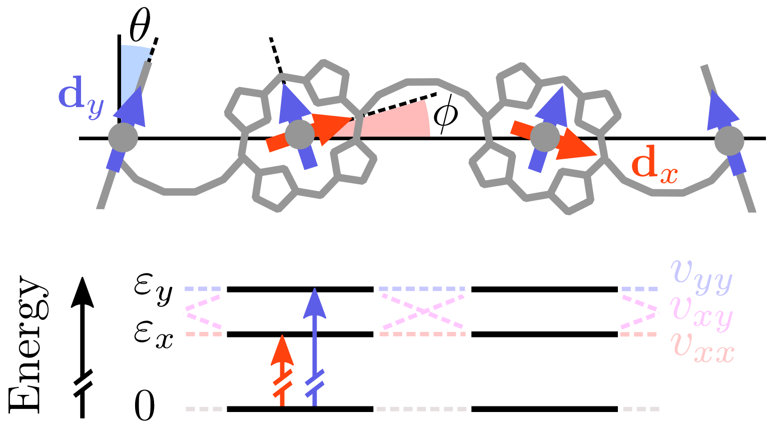

Fig. S39 Geometry of the porphyrin Q-band transition dipole moments on a section of the nanoring (sideview) and schematics of the associated transition energies. Interactions and mixing between monomeric bands are represented as colored dashed lines. The energies ε_x and ε_y represent the energy gap associated with the Q_x and Q_y transitions of a single porphyrin unit. In our model, these individual transitions interact through the coupling constants v_{xx}, v_{xy} and v_{yy}, thus giving rise to delocalized exciton bands spreading across the nanoring.

deviation $\sigma^{x,y}_{\text{global}}$. Nanorings can also experience a stochastic energy shifts due to the interaction between individual porphyrin subunits and the template, which can be mimicked by considering uncorrelated fluctuations of the energies ε_x and ε_y. Also these are assumed to be normally distributed, with standard deviation $\sigma^{x,y}_{\text{local}}$. The stochastic part of the ring Hamiltonian can thus be written as

$$\delta H_{\text{ring}} = \sum_{n=1}^{N} \left[(\delta\varepsilon_x + \delta\varepsilon_x^n)|Q_x^n\rangle\langle Q_x^n| + (\delta\varepsilon_y + \delta\varepsilon_y^n)|Q_y^n\rangle\langle Q_y^n| \right]. \quad \text{(S2)}$$

In general, the local noise source also contributes to homogeneous broadening. We assume that the Q_x and Q_y transitions undergo dephasing with the same rate γ, originating from the interaction of the electrons of the porphyrin with the same set of environmental vibrations.

In order to determine the absorption spectrum of the nanoring, we diagonalize the Hamiltonian $H_{\text{ring}} + \delta H_{\text{ring}}$, finding its eigenstates $|\psi_k\rangle$ and eigenenergies E_k. Each state contributes to the spectrum with an amplitude given by the square of its transition dipole moment $\mathbf{d}_k = \sum_n (\langle Q_x^n|\psi_k\rangle \mathbf{d}_x^n + \langle Q_y^n|\psi_k\rangle \mathbf{d}_y^n)$. The optical dephasing rate of state $|\psi\rangle_k$ can be calculated as $\gamma_k = \text{IPR}_k^{-1}\gamma$, where $\text{IPR}_k = 1/\sum_n (|\langle Q_x^n|\psi_k\rangle|^2 + |\langle Q_y^n|\psi_k\rangle|^2)^2$ is the inverse participation ratio, quantifying the delocalization of a state over the nanoring.[15] Homogeneous broadening in absorption spectra calculations can be accounted for by dressing each exciton state with a Lorentzian function with full width at half maximum γ_k. This procedure is repeated for each realization of the stochastic part of the Hamiltonian δH_{ring} and the ensemble average yields the absorption spectrum.

Nanoring absorption spectrum

The experimental molar extinction coefficient of the nanoring ***c*-P6·T6*** is shown in Fig. S40 (black line) along with the theoretical result (purple line). Our model reproduces well the main features of the spectrum with the parameters summarized in Table S6. The lowest-energy transition at 910 nm, which is responsible for most of the fluorescence from the nanoring, is strongly forbidden, while the lowest-energy peak in the absorption is composed by two distinct and almost degenerate transitions. This is consistent with previous calculations[6,7] and 2DES

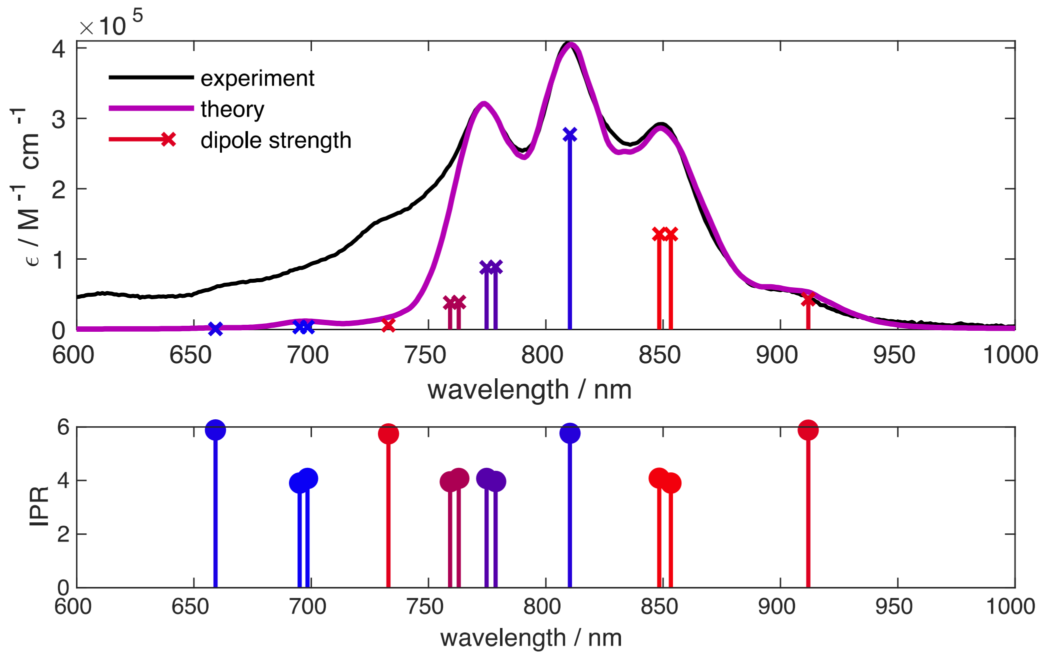

Fig. S40 Top: Absorption spectrum of the templated nanoring. The experimental spectrum is shown in black, the theoretical one in purple. Average wavelengths and oscillator strengths of the ring excitons are shown as sticks. Excitons shown in red (blue) have a prominent Q_x (Q_y) character. Bottom: Average IPR of the Q-band excitations, ordered according to their average wavelength. All averages are obtained from 10^4 stochastic realizations. Same color coding as above.

data.[12] Our model allows us to access the nature of the excited states of the nanoring. In particular, we determine which states exhibit a predominant Q_x (Q_y) character, as represented by the color of the vertical sticks in Fig. S40. We can also check that excitons delocalize over most of the nanoring, as shown by the inverse participation ratio (IPR) in Fig. S40 (bottom).

Dye absorption spectrum

We now consider the rotaxane-encapsulated dye **Cy7⊂T6***. In Fig. S41 we compare the experimental spectrum (black solid line) to the one obtained with Kubo's lineshape theory (green dotted line)[16] (see caption for details). The agreement is excellent, however, we would like to describe the dye using the same theory that we used for the nanoring, keeping the model as simple as possible. We therefore neglect the sideband, and consider only the zero phonon line (green solid line), which enters our model as an additional state $|\mathrm{Cy7}\rangle$ at an energy of $\varepsilon_{\mathrm{Cy7}} = 12850$ cm^{-1}. The transition dipole moment associated to this state $\mathbf{d}_{\mathrm{Cy7}}$ is oriented perpendicularly to the plane of the nanoring and has a magnitude of 10.25 D. The spectrum of the zero phonon line can be obtained by dressing this sharp level with a Lorentzian homogeneous lineshape with full width at half maximum $\gamma_{\mathrm{Cy7}} = 50$ cm^{-1} and a Gaussian inhomogeneous broadening with standard deviation $\sigma_{\mathrm{Cy7}} = 300$ cm^{-1}.

Nanoring-dye interaction

In our target molecular complex, **Cy7⊂*c*-P6·T6***, the dye is placed at the center of the nanoring. In principle, the close distance between the dye and the porphyrins in the nanoring allows for a

Table S6: Parameters of the excitonic model used in Fig. S40.

	Value	Parameter description
ε_x	12460 cm^{-1}	Q_x transition energy
ε_y	13600 cm^{-1}	Q_y transition energy
v_{xx}	-660 cm^{-1}	Q_x-Q_x coupling
v_{yy}	-710 cm^{-1}	Q_y-Q_y coupling
v_{xy}	220 cm^{-1}	Q_x-Q_y coupling
d_x	4.80 D	Q_x transition dipole
d_y	6.96 D	Q_y transition dipole
γ	350 cm^{-1}	homogeneous linewidth (monomer)
$\sigma^{x,y}_{\text{global}}$	220, 200 cm^{-1}	global inhomogeneous broadening
$\sigma^{x,y}_{\text{local}}$	100 cm^{-1}	local inhomogeneous broadening
ϕ	8°	Q_x dipole tilt away from the plane
θ	41°	Q_y dipole tilt towards the center

coherent exchange of excitations described by an Hamiltonian of the form

$$H_{\text{dye-ring}} = \sum_{n=1}^{N} \left(v_x^n |\text{Cy7}\rangle\langle \text{Q}_x^n| + v_y^n |\text{Cy7}\rangle\langle \text{Q}_y^n| + \text{H.c.} \right)$$
$$= \sum_k V_k |\text{Cy7}\rangle\langle \psi_k| + \text{H.c.}, \quad \text{(S3)}$$

where $V_k = \sum_n (\langle \text{Q}_x^n|\psi_k\rangle v_x^n + \langle \text{Q}_y^n|\psi_k\rangle v_y^n)$. The simplest option is to estimate of the couplings between individual porphyrins and the central dye as arising from interaction between point-dipoles. For the Q_x transitions, we have

$$v_x^n = \frac{C}{R^3} \left[\mathbf{d}_x^n \cdot \mathbf{d}_{\text{Cy7}} - 3(\mathbf{d}_x^n \cdot \mathbf{r}_n)(\mathbf{d}_{\text{Cy7}} \cdot \mathbf{r}_n) \right] \quad \text{(S4)}$$

with analogous expressions for the Q_y transitions, where $\mathbf{r}_n$ are the unit vectors pointing from the center of the nanoring to the position of the porphyrins. The radius of the nanoring is fixed to $R = 1.17$ nm by our molecular mechanics simulations, whereas the constant C is tuned to achieve the best agreement with the absorption spectrum of **Cy7⊂*c*-P6·T6*** in the spectral region overlapping with the main peak of the isolated dye. Simple extensions to this model can be achieved by taking into account the finite size of these dipoles, which is informed by their molecular structure.

The coherent coupling (S3), in general, can lead to coherent delocalization of the electronic excitations between dye and nanoring. These excitations can be obtained by diagonalizing the total Hamiltonian $H = H_{\text{ring}} + \delta H_{\text{ring}} + H_{\text{dye-ring}} + H_{\text{dye}}$, where $H_{\text{dye}} = \varepsilon_{\text{Cy7}}|\text{Cy7}\rangle\langle\text{Cy7}|$. Thus, one obtains eigenstates $|\Psi_\alpha\rangle = a_\alpha|\text{Cy7}\rangle + \sum_k b_{\alpha,k}|\psi_k\rangle$ and eigenenergies $\mathscr{E}_\alpha$. These states can be used to calculate the absorption spectrum of the interactiong nanoring-dye system. To do so, one needs to determine their transition dipole moments $\mathbf{D}_\alpha = a_\alpha \mathbf{d}_{\text{Cy7}} + \sum_k b_{\alpha,k}\mathbf{d}_k$ and their optical dephasing rate $\Gamma_\alpha = |a_\alpha|^4 \gamma_{\text{Cy7}} + \sum_k |b_{\alpha,k}|^4 \gamma_k$ and follow the same procedure described above to obtain the absorption spectrum of the nanoring.

In the case in which the coherent couplings V_k are much smaller than the relative dephasing rates of the dye and nanoring $\gamma_{\text{Cy7}} + \gamma_k$, one can use multi-chromophoric Förster resonant

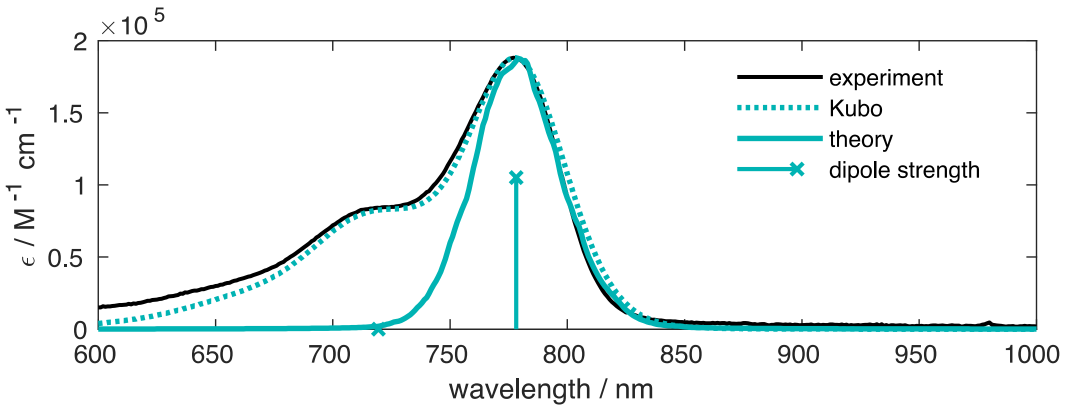

Fig. S41 Absorption spectrum of **Cy7⊂T6***. The experimental spectrum (black solid line) can be fitted with Kubo's lineshape theory (green dotted line) assuming that the molecular vibrations are described by a single anti-symmetrized Lorentzian spectral density with central frequency 1280 cm^{-1}, reorganization energy 720 cm^{-1} and width 350 cm^{-1}, inhomogeneously broadened with a standard deviations of 280 cm^{-1}. Alternatively, we can just describe the zero phonon line (green solid line) with a Lorentzian lineshape with full width at half maximum $\gamma_{\text{Cy7}} = 50$ cm^{-1}, experiencing inhomogeneous broadening with standard deviation $\sigma_{\text{Cy7}} = 300$ cm^{-1} and dipole strength $d_{\text{Cy7}} = 10.25$ D. The theoretical results are obtained after averaging over 10^4 ensemble realizations.

energy transfer theory (MC-FRET)[17] in order to estimate the incoherent transfer rate between the dye and the nanoring. One, thus, obtains the rates

$$K_{\text{Cy7}\to\psi_k} = \frac{2|V_k|^2}{\hbar} \frac{\gamma_{\text{Cy7}} + \gamma_k}{(\varepsilon_{\text{Cy7}} - E_k)^2 + (\gamma_{\text{Cy7}} + \gamma_k)^2}. \tag{S5}$$

Absorption spectrum of the target complex

Having set up a model of the nanoring ***c*-P6·T6*** and of the dye **Cy7⊂T6*** that can explain the essential features of their absorption spectra and that allows to calculate their excitonic interactions, we can proceed to compute the absorption spectrum of the target molecular structure **Cy7⊂*c*-P6·T6***.

In order to see clearly the effects of the dye-nanoring coupling, we deliberately choose a large value of the constant C in eq. (S4) giving rise to couplings $|V_k|$ peaking at 50 cm^{-1}. This leads to the absorption spectrum shown in Fig. S42 (top). We see that the most evident effect of this interaction is a dipole redistribution from the 810 nm band of the nanoring to the dye, which is resonant to the 760 nm band. This dipole redistribution is evidently too large when compared to experimental data, therefore we tune the interaction down by a factor of two, so that the largest couplings are now around 25 cm^{-1}. The result is shown in Fig. S42 (bottom). The absorption spectrum still shows some dipole redistribution between the same bands, but now is closer to the experimental result. In particular, the 760 nm band now has a larger absorption cross section with respect to the non-interacting dye-nanoring system (dotted line). The same feature is observed when comparing the experimental spectra of **Cy7⊂*c*-P6·T6*** and **Cy7⊂T6*** + ***c*-P6·T6***. However, our model cannot fully account for the changes observed for the bands at 810 nm and 850 nm. Theoretically, the small redistribution of absorption amplitudes between dye and nanoring implies a coherent redirection of excitations from the nanoring to the central dye. The redistribution is nevertheless smaller than other changes observed after the inclusion of the dye, which are not captured by our theory, restraining an unambiguous experimental confirmation of this feature as originating from coherent ring-dye dynamics.

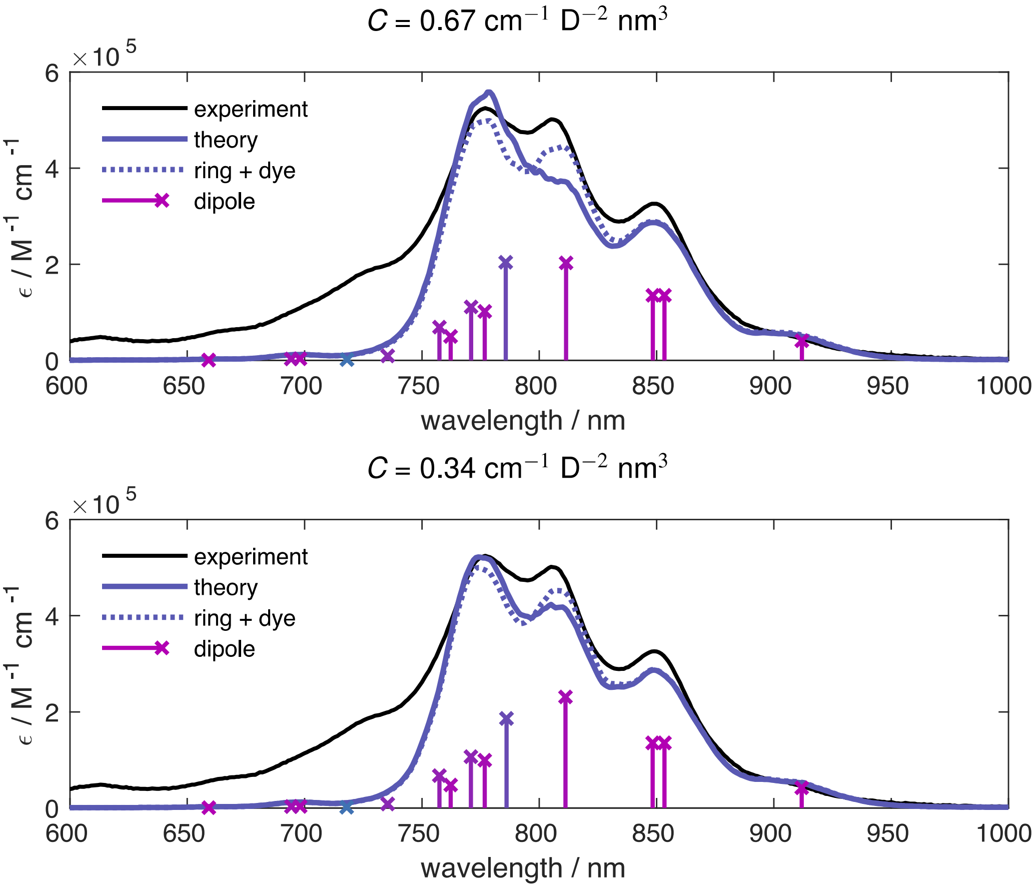

Fig. S42 Absorption spectrum of the interacting nanoring-dye system. The experimental spectrum is shown in black, whereas the theoretical one in blue. The sum of the theoretical spectra of dye and nanoring is also shown for reference (dotted blue line). Average wavelengths and oscillator strengths of the excitons of the interacting system are shown as sticks. Excitons shown in purple (green) have a prominent nanoring (dye) character. The dye-nanoring interactions are computed using the point-dipole approximation for both the dye and the porphyrins. The results are obtained as averages over 10^4 realizations of static disorder. Top: $C = 0.67$ cm^{-1}D^{-2} nm^3. Bottom: $C = 0.34$ cm^{-1}D^{-2} nm^3.

The dipole redistribution can be better quantified by comparing the dipole strength on the dye when the dye-nanoring interaction is present with its value $|\mathbf{d}_{\text{Cy7}}|^2$ with no interactions. The total dipole strength residing on the dye can be quantified as $\sum_\alpha |\mathbf{D}_\alpha|^2 |\langle \text{Cy7}|\Psi_\alpha\rangle|^2$, i.e. as a sum of the dipole strengths of the states $|\Psi_\alpha\rangle$, weighted by the probability of that state being on the dye. The dipole redistribution from the nanoring to the dye, as a fraction f of the total dipole strength, can thus be defined as

$$f_{\text{dye}} = \frac{\sum_\alpha |\mathbf{D}_\alpha|^2 |\langle \text{Cy7}|\Psi_\alpha\rangle|^2 - |\mathbf{d}_{\text{Cy7}}|^2}{\sum_\alpha |\mathbf{D}_\alpha|^2}, \tag{S6}$$

and, analogously for the nanoring,

$$f_{\text{ring}} = \frac{\sum_\alpha |\mathbf{D}_\alpha|^2 \sum_k |\langle \psi_k|\Psi_\alpha\rangle|^2 - \sum_k |\mathbf{d}_k|^2}{\sum_\alpha |\mathbf{D}_\alpha|^2}. \tag{S7}$$

The conservation of the total dipole strength imposes that $f_{\text{dye}} + f_{\text{ring}} = 0$, which can be easily checked. Fig. S43 shows a clear redistribution of dipole strength from the nanoring to the dye, which hints to an increased absorption on the dye due to the coherent dye-ring coupling. The

effect is rather small (around 2% of the total dipole strength, or 10% of the bare dye's dipole strength), but nonetheless clearly present and robust to disorder.

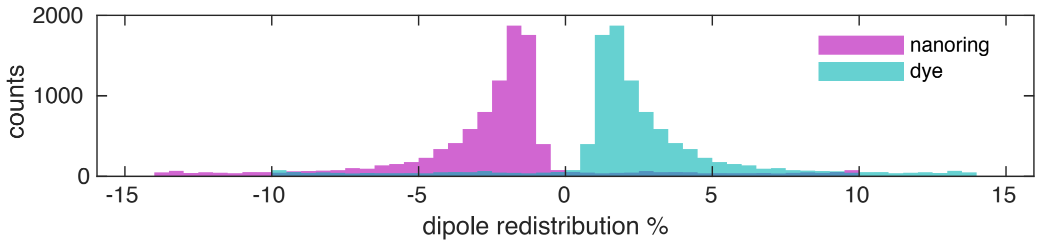

Fig. S43 Fraction of the total dipole strength that is redistributed to the dye f_{dye} (green) or to the nanoring f_{ring} (purple) due to their coherent coupling. The histograms are obtained for 10^4 realizations of static disorder. The fact that they are symmetric around zero reflects the conservation of the total dipole strength.

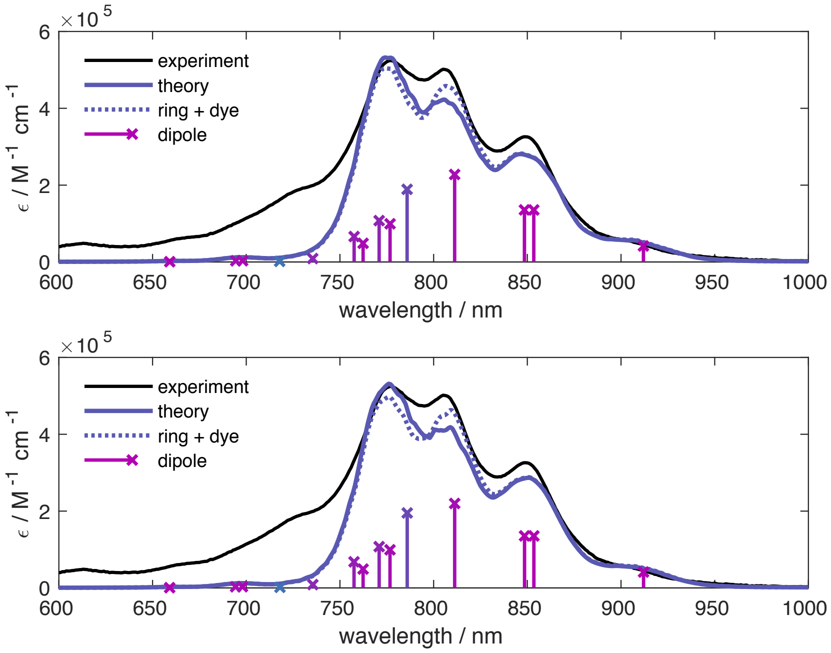

Fig. S44 Absorption spectrum of the interacting nanoring-dye system, using the extended dipole approach for the dye-ring coupling. Top: the dye is treated as an extended dipole of length 1.4 nm, interacting with point-like porphyrin dipoles. Bottom: both dye and porphyrins are treated as extended dipole. The length of both Q_x and Q_y dipoles is 0.6 nm.

Very similar results are obtained when considering the finite size of the transition dipoles. In the top panel of Fig. S44, we show the absorption spectrum of the dye-nanoring system when the Q_x and Q_y dipoles of the porphyrins are still point-like, but the dipole of the dye is taken to have a length of 1.4 nm. By taking also the porphyrin dipoles as extended with a length of 0.6 nm, as shown in the bottom panel, the main features of the spectrum remain unchanged. In both cases, the maximum couplings $|V_k|$ are close to the ones arising from point-dipole interactions. This similarity enables our use of the point-dipole approximation for the calculations presented in Fig.4 in the main text.

The presence of dipole redistribution between the dye and the 810 nm band can be confirmed by plotting the absolute value of the nanoring-dye couplings $|V_k|$ as a function of the wavelength of the nanoring excitons $\lambda_k = hc/E_k$ (Fig. S45, blue circles). This can be easily

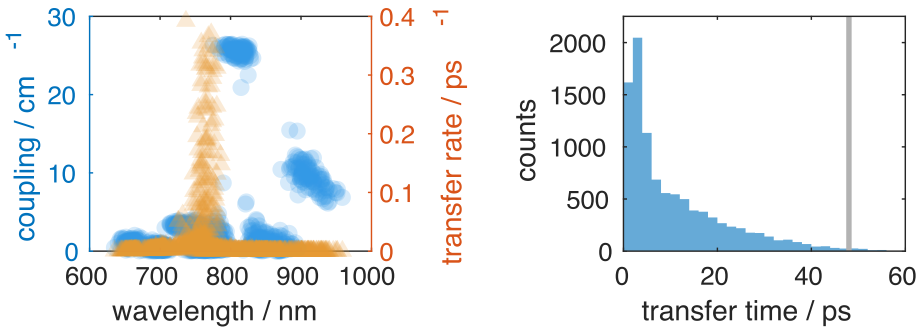

Fig. S45 Left: Coupling $|V_k|$ (blue circles) and incoherent transfer rate $K_{\text{Cy7}\to\psi_k}$ (orange triangles) between the dye and the nanoring, plotted as a function of the wavelength of the state on the nanoring. Results are shown for 100 realizations of static disorder. Right: Distribution of the total energy transfer time τ from the dye to the nanoring obtained for 10^4 realizations of static disorder. 99% of the realizations lie below the vertical gray line.

understood recalling that the 810 nm band has a strong Q_y character: the transition dipoles of the nanoring excitons in this spectral region are all aligned parallel to the transition dipole moment of the dye.

Absorption measurements are sensitive to the dynamics of the electronic excitations on a very short timescale, typically shorter than a couple of hundred femtoseconds. Although we see an effect of the coherent dye-nanoring interaction in the absorption spectrum, the energy transfer between the two is likely to be mostly incoherent, given the fact that the coupling (≈ 25 cm^{-1}) is much smaller than the dephasing rates ($\gtrsim 100$ cm^{-1}). We compute the dye-nanoring transfer rate $K_{\text{Cy7}\to\psi_k}$ for nanoring excitons at different wavelengths $\lambda_k = hc/E_k$, and also the total dye-nanoring transfer time τ by integrating over λ_k, i.e. $\tau = 1/\sum_k K_{\text{Cy7}\to\psi_k}$. From Fig. S45 we see that the fastest transfer occurs on a timescale of about 3 ps and it is achieved by the 760 nm band on the nanoring, which is resonant to the zero phonon line of the dye. This shows that, in this case, the resonance condition is more important than having strong coupling for fast energy transfer. Another interesting observation is that very few realizations show slow transfer rates. From the distribution of the transfer time τ one can see that 99% of the stochastic realizations lead to transfer times shorter than roughly 50 ps. This timescale is about one order of magnitude shorter than typical fluorescence times from organic dyes. After initialization on the central dye, therefore, the excitation is transferred to the nanoring before it can fluoresce. On the nanoring, the excitation can undergo vibrational relaxation and emit at later times from the lowest excited state at 910 nm. This explains the extremely small fluorescence quantum yield of the dye when incapsulated in the nanoring.

8 References

1 G. R. Fulmer, A. J. M. Miller, N. H. Sherden, H. E. Gottlieb, A. Nudelman, B. M. Stoltz, J. E. Bercaw, K. I. Goldberg, *Organometallics* **2010**, *29*, 2176–2179.

2 C. K. Yong, P. Parkinson, D. V. Kondratuk, W. H. Chen, A. Stannard, A. Summerfield, J. K. Sprafke, M. C. O'Sullivan, P. H. Beton, H. L. Anderson, L. M. Herz, *Chem. Sci.* **2015**, *6*, 181–189.

3 M. V. Rekharsky, Y. Inoue, *Chem. Rev.* **1998**, *98*, 1875–1918.

4 K. Takeo, K. Uemura, H. Mitoh, *J. Carbohydr. Chem.* **1988**, *7*, 293–308.

5 L. A. Babadzhanova, N. V. Kirij, Y. L. Yagupolskii, W. Tyrra, D. Naumann, *Tetrahedron* **2005**, *61*, 1813–1819.

6 M. Hoffmann, J. Kärnbratt, M. H. Chang, L. M. Herz, B. Albinsson, H. L. Anderson, *Angew. Chem. Int. Ed.* **2008**, *47*, 4993–4996.

7 J. K. Sprafke, D. V. Kondratuk, M. Wykes, A. L. Thompson, M. Hoffmann, R. Drevinskas, W. H. Chen, C. K. Yong, J. Kärnbratt, J. E. Bullock, M. Malfois, M. R. Wasielewski, B. Albinsson, L. M. Herz, D. Zigmantas, D. Beljonne, H. L. Anderson, *J. Am. Chem. Soc.* **2011**, *133*, 17262–17273.

8 (a) J. Ivri, Z. Burshtein, E. Miron, *App. Optics* **1991**, *30*, 2484–2488; (b) M. Y. Berezin, K. Guo, W. Akers, R. E. Northdurft, J. P. Culver, B. Teng, O. Vasalatiy, K. Barbacow, A. Gandjbakhche, G. L. Griffiths, S. Achilefu, *Biophys. J.* **2011**, *100*, 2063–2072.

9 F. Caycedo-Soler, C. A. Schroeder, C. Autenrieth, A. Pick, R. Ghosh, S. F. Huelga, M. B. Plenio, *J. Phys. Chem. Lett.* **2017**, *8*, 6015–6021.

10 F. V. A. Camargo, H. L. Anderson, S. R. Meech, I. A. Heisler, *J. Phys. Chem. A* **2015**, *119*, 95–101.

11 F. V. A. Camargo, C. R. Hall, H. L. Anderson, S. R. Meech, I. A. Heisler, *Struct. Dyn.* **2016**, *3*, 023608.

12 V. Butkus, J. Alster, E. Bašinskaite, R. Augulis, P. Neuhaus, L. Valkunas, H. L. Anderson, D. Abramavicius, D. Zigmantas, *J. Phys. Chem. Lett.* **2017**, *8*, 2344–2349.

13 V. May, O. Kühn, Charge and Energy Transfer Dynamics in Molecular Systems (WILEY-VCH Verlag GmbH & Co. KGaA, Weinheim, 2004).

14 M. Bednarz, P. Reineker, E. Mena-Osteritz, P. Bäuerle, *J. Lumin.* **2004**, *110*, 225–231.

15 F. Caycedo-Soler, J. Lim, S. Oviedo-Casado, N. F. van Hulst, S. F. Huelga, M. B. Plenio, *J. Phys. Chem. Lett.* **2018**, *9*, 3446–3453.

16 R. Kubo, *Adv. Chem. Phys.* **1969**, *16*, 101–127.

17 S. Jang, M. D. Newton, R. J. Silbey, *Phys. Rev. Lett.* **2004**, *92*, 218301.
